# Prenatal Health Care Outcomes Before and During the COVID-19 Pandemic Among Pregnant Individuals and Their Newborns in an Integrated US Health System

**DOI:** 10.1001/jamanetworkopen.2023.24011

**Published:** 2023-07-18

**Authors:** Assiamira Ferrara, Mara Greenberg, Yeyi Zhu, Lyndsay A. Avalos, Amanda Ngo, Jun Shan, Monique M. Hedderson, Charles P. Quesenberry

**Affiliations:** 1 Division of Research, Kaiser Permanente Northern California, Oakland; 2 Obstetrics and Gynecology, Kaiser Permanente Northern California, Oakland; 3 Regional Perinatal Service Center, Kaiser Permanente Northern California, Santa Clara; 4 Department of Health Systems Science, Kaiser Permanente Bernard J. Tyson School of Medicine, Pasadena, California

## Abstract

**Importance:**

The COVID-19 pandemic accelerated the use of telemedicine. However, data on the integration of telemedicine in prenatal health care and health outcomes are sparse.

**Objective:**

To evaluate a multimodal model of in-office and telemedicine prenatal health care implemented during the COVID-19 pandemic and its association with maternal and newborn health outcomes.

**Design, Setting, and Participants:**

This cohort study of pregnant individuals using longitudinal electronic health record data was conducted at Kaiser Permanente Northern California, an integrated health care system serving a population of 4.5 million people. Individuals who delivered a live birth or stillbirth between July 1, 2018, and October 21, 2021, were included in the study. Data were analyzed from January 2022 to May 2023.

**Exposure:**

Exposure levels to the multimodal prenatal health care model were separated into 3 intervals: unexposed (T1, birth delivery between July 1, 2018, and February 29, 2020), partially exposed (T2, birth delivery between March 1, 2020, and December 5, 2020), and fully exposed (T3, birth delivery between December 6, 2020, and October 31, 2021).

**Main Outcomes and Measures:**

Primary outcomes included rates of preeclampsia and eclampsia, severe maternal morbidity, cesarean delivery, preterm birth, and neonatal intensive care unit (NICU) admission. The distributions of demographic and clinical characteristics, care processes, and health outcomes for birth deliveries within each of the 3 intervals of interest were assessed with standardized mean differences calculated for between-interval contrasts. Interrupted time series analyses were used to examine changes in rates of perinatal outcomes and its association with the multimodal prenatal health care model. Secondary outcomes included gestational hypertension, gestational diabetes, depression, venous thromboembolism, newborn Apgar score, transient tachypnea, and birth weight.

**Results:**

The cohort included 151 464 individuals (mean [SD] age, 31.3 [5.3] years) who delivered a live birth or stillbirth. The mean (SD) number of total prenatal visits was similar in T1 (9.41 [4.75] visits), T2 (9.17 [4.50] visits), and T3 (9.15 [4.66] visits), whereas the proportion of telemedicine visits increased from 11.1% (79 214 visits) in T1 to 20.9% (66 726 visits) in T2 and 21.3% (79 518 visits) in T3. NICU admission rates were 9.2% (7014 admissions) in T1, 8.3% (2905 admissions) in T2, and 8.6% (3615 admissions) in T3. Interrupted time series analysis showed no change in NICU admission risk during T1 (change per 4-week interval, −0.22%; 95% CI, –0.53% to 0.09%), a decrease in risk during T2 (change per 4-week interval, −0.91%; 95% CI, –1.77% to −0.03%), and an increase in risk during T3 (change per 4-week interval, 1.75%; 95% CI, 0.49% to 3.02%). There were no clinically relevant changes between T1, T2, and T3 in the rates of risk of preeclampsia and eclampsia (change per 4-week interval, 0.76% [95% CI, 0.39% to 1.14%] for T1; −0.19% [95% CI, –1.19% to 0.81%] for T2; and −0.80% [95% CI, –2.13% to 0.55%] for T3), severe maternal morbidity (change per 4-week interval , 0.12% [95% CI, 0.40% to 0.63%] for T1; −0.39% [95% CI, –1.00% to 1.80%] for T2; and 0.99% [95% CI, –0.88% to 2.90%] for T3), cesarean delivery (change per 4-week interval, 0.06% [95% CI, –0.11% to 0.23%] for T1; −0.03% [95% CI, –0.49% to 0.44%] for T2; and −0.05% [95% CI, –0.68% to 0.59%] for T3), preterm birth (change per 4-week interval, 0.23% [95% CI, –0.11% to 0.57%] for T1; −0.37% [95% CI, –1.29% to 0.55%] for T2; and −0.15% [95% CI, –1.41% to 1.13%] for T3), or secondary outcomes.

**Conclusions and Relevance:**

These findings suggest that a multimodal prenatal health care model combining in-office and telemedicine visits performed adequately compared with in-office only prenatal health care, supporting its continued use after the pandemic.

## Key Points

**Question:**

Did prenatal health care delivered by a multimodal model of in-office and telemedicine visits implemented during the COVID-19 pandemic result in equal maternal and newborn health outcomes compared with prepandemic in-office health care?

**Findings:**

In this cohort study of 151 464 pregnant individuals, after implementation of a multimodal prenatal health care model with telemedicine and in-office visits during the pandemic, there were no changes in rates of preeclampsia and eclampsia, severe maternal morbidity, cesarean delivery, and preterm birth compared with the prepandemic rates; however, there was an increase in the rate of neonatal intensive care unit admissions during the second pandemic period.

**Meaning:**

These findings suggest that a multimodal prenatal health care model combining in-office and telemedicine visits performed adequately compared with in-office only prenatal health care, supporting its continued use after the pandemic.

## Teaser

This cohort study compares a multimodal model of in-office and telemedicine prenatal health care model implemented during the COVID-19 pandemic with prepandemic in-office only health care and its association with maternal and newborn health outcomes.

## Introduction

Across the US, the COVID-19 pandemic has been an unprecedented challenge for the health care of pregnant individuals, a population in need of frequent contacts with the health care system. The COVID-19 pandemic has also led to a rapid adoption of telemedicine,^1^ offering a unique opportunity to reevaluate and reorganize prenatal health care delivery. Telemedicine may become an important modality integrated into prenatal health care beyond the pandemic since it can reach patients in rural areas and address other barriers to health care, such as lack of transportation and limited time due to work or childcare.^2^ However, data on the integration of telemedicine in prenatal health care and prenatal health outcomes are sparse.

To reduce unnecessary exposure of pregnant individuals to COVID-19, Kaiser Permanente Northern California (KPNC) implemented telemedicine in prenatal health care. Taking advantage of the pandemic-related change constituted by the implementation of a multimodal prenatal health care model of in-office and telemedicine, we investigated the association of this multimodal model with maternal and newborn health outcomes.

We hypothesized that the delivery of prenatal health care by a multimodal model of telemedicine and in-office visits during the pandemic was equally effective regarding health outcomes as standard in-office health care before the pandemic. We also explored whether the outcomes of the multimodal health care model were similar among individuals of different races and ethnicities, those with a disadvantaged socioeconomic background, with a preferred language other than English, or those living in a rural area.

## Methods

This cohort study was approved by the KPNC institutional review board, which waived the requirement for participant informed consent because the study used only electronic data and the large sample size did not make it feasible to obtain authorization from each individual. This study follows the Strengthening the Reporting of Observational Studies in Epidemiology (STROBE) reporting guideline.^3^ This data-only project was conducted at KPNC, an integrated health care delivery system, using longitudinal data from electronic health records (EHRs). KPNC serves 4.5 million individuals (including approximately 60 000 pregnant individuals per year), accounting for approximately 40% of the population residing in the served geographic area (including both urban and rural areas), and is representative of the underlying population regarding race and ethnicity, education level, and obesity rates, differing only slightly from the underlying population in regard to income.^4,5,6^

Using EHR data, we identified all pregnant individuals who delivered a live birth or stillbirth between July 1, 2018, and October 21, 2021. We searched EHRs for all encounter types (eg, in-office or telemedicine) and identified diagnoses and procedures according to the *International Statistical Classification of Diseases and Related Health Problems, Tenth Revision (ICD-10)* codes (eTable 1 in Supplement 1), laboratory tests and results, and medications from 2 years before the last menstrual period through pregnancy.

Prior to March 13, 2020, KPNC followed a prenatal health care delivery schedule as described by the American College of Obstetricians and Gynecologists,^7^ with all standard visits and screenings taking place in person. As of March 13, 2020, changes in prenatal health care were implemented. For patients at low risk (ie, patients younger than 40 years) and patients with specific moderate risk conditions including gestational diabetes (GD) receiving diet therapy only, chronic hypertension without medication management, prior cesarean delivery, and other conditions per clinician discretion, the following 4 standard in-office prenatal health care visits were converted to telemedicine visits (either by video or by telephone according to the patient’s preference): 10- to 12-week visit (after completion of the first trimester office physical examination and ultrasonography examination), 22-week visit, 28-week visit, and 32-week visit.

To reflect the levels that a pregnancy was exposed to the multimodal prenatal health care model related to the COVID-19 pandemic, dates of birth delivery were categorized in 3 time intervals: T1, unexposed (July 1, 2018, to February 29, 2020); T2, partially exposed (March 1, 2020, to December 5, 2020); and T3, fully exposed (December 6, 2020, to October 31, 2021). Individuals with birth delivery in T2 did not experience the multimodal prenatal health care model for the entire duration of the pregnancy; the fraction of a pregnancy exposed to the multimodal health care model increased from 0% to 100% with each additional day during this time interval. In contrast, individuals with birth delivery in T3 experienced the multimodal health care model for the entire duration of the pregnancy.

Primary outcomes included rates of preeclampsia and eclampsia, severe maternal morbidity, cesarean delivery, preterm birth, and neonatal intensive care unit (NICU) admission. We defined preeclampsia and eclampsia by *ICD-10* codes or meeting both of the following criteria after 20 weeks of gestation: (1) at least 2 blood pressure readings above 140/90 mm Hg on 2 occasions at least 4 hours apart or at least 2 readings above 160/110 mm Hg on 2 occasions within 1 hour and (2) new onset of proteinuria, thrombocytopenia, or pulmonary edema.^8^ We used the Centers for Disease Control and Prevention Callaghan criteria^9,10,11^ to define the presence of severe maternal morbidity, which comprised 21 conditions occurring at any time during pregnancy or delivery.^10^ Preterm birth was defined using a previously validated,^12^ obstetric, estimate-based measure of gestational age at delivery (<37 weeks). We further classified preterm birth as spontaneous (based on hospital discharge codes for early spontaneous onset of delivery and/or premature rupture of membranes) or medically indicated (if individuals had no indication of spontaneous birth and a hospital discharge diagnosis of induced and/or cesarean delivery). We used *ICD-10* codes to define cesarean deliveries and NICU admissions.

Secondary outcomes included gestational hypertension, GD, depression, venous thromboembolism (VTE), newborn Apgar score (<7), transient tachypnea, and birth weight. We defined gestational hypertension by *ICD-10* codes, use of antihypertensive medications, or at least 2 blood pressure readings of 140/90 mm Hg or higher on 2 occasions at least 4 hours apart after 20 weeks of gestation, and no diagnosis of chronic hypertension.^8^ We defined GD according to the Carpenter and Coustan glucose thresholds,^13^ by diagnosis or use of antidiabetic medications among individuals who did not have pregestational diabetes.^14^ *ICD-10* codes were used to define prenatal depression, VTE, and transient tachypnea. Prenatal depression severity was defined according to Patient Health Questionnaire screening scores (moderate to severe depression score ≥10).^15^ We searched EHRs for 5-minute Apgar scores. We defined being small for gestational age (SGA) and large for gestational age (LGA) as gestational age–specific and sex-specific birth weight less than the 10th percentile and greater than the 90th percentile, respectively.^16^

Age, self-reported race and ethnicity, residential addresses, and preferred language were captured in the outpatient setting or at hospital admission. Parity, smoking, prepregnancy height and weight, and health outcomes were obtained from EHRs, where data are collected by health care clinicians. Race was defined as American Indian or Alaska Native, Asian or Pacific Islander, Black, multiracial, or White. All patients who self-identified as Hispanic ethnicity, regardless of race, were categorized as Hispanic. Race and ethnicity were included because of known associations with perinatal outcomes.^17^ Residential addresses were used to calculate the neighborhood deprivation index (NDI), a composite index where higher values indicate more disadvantaged neighborhood characteristics,^18^ and for identifying whether an individual lived in an urban or rural area^19^ (eAppendix in Supplement 1). According to the World Health Organization recommendations on specific body mass index (BMI, calculated as weight in kilograms divided by height in meters squared) cutoffs for individuals of different races and ethnicities,^20^ we categorized individuals as underweight, normal weight, overweight, and obese.

### Statistical Analysis

The distributions of demographic and clinical characteristics, care processes, and health outcomes for birth deliveries within each of the 3 intervals of interest were assessed with standardized mean differences (SMDs) calculated for between-interval contrasts. Analyses examined changes in rates of perinatal outcomes in relation to the multimodal prenatal health care model implemented during the COVID-19 pandemic using an interrupted time series (ITS) design.^21^ Log binomial regression was used to examine each binary outcome in relation to date of birth delivery (week), providing point and interval estimates of relative percentage change in outcome probability associated with change in time, scaled to a 4-week change for the purpose of interpretability of small effect sizes. Poisson regression was used for point and interval estimation of relative percentage change in means for outcomes characterized as counts in relation to a 4-week change in birth delivery date. Analyses were performed with and without adjustment for age, race and ethnicity, prepregnancy BMI, and NDI.

In accordance with the ITS method,^21,22^ prenatal outcomes before and during the pandemic were modeled using piecewise linear segmented regression, allowing a change in slope associated with time at 2 inflection points: March 1, 2020 (initiation of the multimodal health care model), and December 6, 2020 (date when individuals were fully exposed to the multimodal health care model). Given the nature of exposure to the multimodal health care model, with the proportion of a pregnancy with a postpandemic birth delivery date that was exposed to the change gradually increasing from 0% to 100% during T2 (March 1, 2020, to December 6, 2020), we hypothesized and allowed for a change in slope associated with calendar time with no immediate or abrupt outcome level change at each of the 2 inflection points. Formal tests of statistical significance for differences in slopes between the 3 time intervals were calculated (2-sided Wald test). *P* < .05 was considered statistically significant. ITS analyses were also performed after stratifying individuals by various sociodemographic factors including race and ethnicity, NDI quartiles, living in an urban or rural area, whether English was the preferred language, and whether they had a low-risk pregnancy. We also presented plots of weekly proportions of birth deliveries with each binary outcome of interest and means among deliveries in each week for variables characterized as counts, with an overlaid plot of estimated values from a segmented linear regression with no covariate adjustment as a visual aid in interpretation of time trends (see eAppendix in Supplement 1 for more details on the statistical analyses). We used multiple imputation^23,24^ for variables with missing data such as age (0.01%), race and ethnicity (2.30%), NDI (0.15%), prepregnancy BMI (3.36%), smoking (0.89%), GD (4.30%), cesarean delivery (5.15%), Apgar score (4.74%), NICU admission (4.74%), and birth weight for gestational age categories (4.76%) (eAppendix and eTable 1 in Supplement 1). Analyses were conducted using SAS/STAT statistical software version 9.4 (SAS Institute Inc) from January 2022 to May 2023.

## Results

Between March 1, 2018, and October 31, 2021, there were 151 464 individuals (mean [SD] age, 31.3 [5.3] years) who delivered a singleton live birth or stillbirth. The cohort included 557 (0.4%) American Indian or Alaska Native individuals, 39 114 (25.8%) Asian or Pacific Islander individuals, 10 013 (6.6%) Black individuals, 42 245 (27.9%) Hispanic individuals, 3932 (2.6%) multiracial individuals, 52 129 (34.4%) White individuals, and 3474 (2.3%) individuals whose race or ethnicity was missing or unknown. Of the 151 464 individuals, 75 836 (50.1%) were unexposed to the multimodal prenatal health care model (T1), 34 799 (23.0%) were partially exposed (T2), and 40 829 (26.9%) were fully exposed (T3). There were negligible differences in the sociodemographic and clinical characteristics between individuals who were partially exposed or fully exposed compared with those who were unexposed (Table 1).

**Table 1.** Characteristics of Individuals Who Delivered a Singleton Live Birth or Stillbirth by Exposure to Implementation of a Multimodal Prenatal Health Care Model During the COVID-19 Pandemic at Kaiser Permanente Northern California, 2018-2021

Characteristic	Patients, No. (%) (N = 151 464)			Standardized mean difference	
	Unexposed (T1) (n = 75 836)	Partially exposed (T2) (n = 34 799)	Fully exposed (T3) (n = 40 829)	T2 vs T1	T3 vs T1
Age, y					
mean (SD)	31.23 (5.3)	31.40 (5.2)	31.49 (5.2)	0.032	0.049
<25	8559 (11.3)	3640 (10.5)	4107 (10.1)	−0.027	−0.040
25-29	18 653 (24.6)	8261 (23.7)	9613 (23.5)	−0.020	−0.025
30-34	27 520 (36.3)	12 854 (36.9)	15 278 (37.4)	0.013	0.023
35-54	21 100 (27.8)	10 042 (28.9)	11 828 (29.0)	0.023	0.025
Missing	4 (<.01)	2 (<.01)	3 (<.01)	NA	NA
Race and ethnicity					
American Indian and Alaska Native	282 (0.4)	119 (0.3)	156 (0.4)	−0.005	0.002
Asian and Pacific Islander	19 799 (26.1)	8947 (25.7)	10 368 (25.4)	−0.009	−0.016
Black	4978 (6.6)	2306 (6.7)	2729 (6.7)	0.003	0.005
Hispanic	20 761 (27.4)	9826 (28.2)	11 658 (28.6)	0.019	0.026
Multiracial	2019 (2.6)	883 (2.6)	1030 (2.6)	−0.008	−0.009
White	26 388 (34.8)	11 866 (34.1)	13 875 (34.0)	−0.015	−0.017
Missing	1609 (2.1)	852 (2.4)	1013 (2.5)	0.022	0.024
Neighborhood Deprivation Index, quartile					
1	20 986 (27.7)	9945 (28.6)	11 526 (28.2)	0.020	0.012
2	17 808 (23.5)	8332 (23.9)	9849 (24.1)	0.011	0.015
3	18 829 (24.8)	8115 (23.3)	9682 (23.7)	−0.035	−0.026
4	18 135 (23.9)	8367 (24.0)	9665 (23.7)	0.003	−0.006
Missing	78 (0.1)	40 (0.1)	107 (0.3)	0.004	0.037
Residing in a neighborhood with mean education level <12 grades	13.38 (10.3)	13.50 (10.4)	13.38 (10.3)	0.011	0.000
Parity					
0	32 895 (43.4)	15 089 (43.4)	17 411 (42.6)	0.000	−0.015
1	26 260 (34.6)	12 021 (34.5)	14 215 (34.8)	−0.002	0.004
≥2	16 120 (21.3)	7329 (21.1)	8578 (21.0)	−0.005	−0.006
Missing	561 (0.7)	360 (1.0)	625 (1.5)	0.031	0.075
Pregestational diabetes	793 (1.0)	465 (1.3)	512 (1.3)	0.027	0.020
Chronic hypertension	3961 (5.2)	1943 (5.6)	2534 (6.2)	0.016	0.042
Prepregnancy body mass index^a^					
Underweight	6344 (8.4)	2823 (8.1)	3179 (7.8)	−0.009	−0.021
Normal weight	26 049 (34.3)	11 523 (33.1)	13 269 (32.5)	−0.026	−0.039
Overweight	20 950 (27.6)	9672 (27.8)	11 548 (28.3)	0.004	0.015
Obese	20 250 (26.7)	9751 (28.0)	11 745 (28.8)	0.030	0.046
Missing	2243 (3.0)	1030 (3.0)	1088 (2.7)	0.000	−0.018
Singleton birth	74 635 (98.4)	34 290 (98.5)	40 285 (98.7)	0.010	0.021
Infant sex					
Female	34 898 (46.0)	16 169 (46.5)	18 840 (46.1)	0.009	0.003
Male	36 814 (48.5)	16 880 (48.5)	19 941 (48.8)	−0.001	0.006
Missing	4124 (5.4)	1750 (5.0)	2048 (5.0)	−0.018	−0.019
Gestational age at delivery, mean (SD), wk	38.67 (2.14)	38.65 (2.10)	38.58 (2.17)	−0.009	−0.039
<22	146 (0.2)	67 (0.2)	91 (0.2)	0.000	0.007
22-31	945 (1.2)	385 (1.1)	543 (1.3)	−0.013	0.007
32-33	619 (0.8)	303 (0.9)	335 (0.8)	0.006	0.000
34-36	4345 (5.7)	2043 (5.9)	2362 (5.8)	0.006	0.002
≥37	69 781 (92.0)	32 001 (92.0)	37 498 (91.8)	−0.002	−0.006
Smoking during pregnancy					
Never	73 684 (97.2)	33 970 (97.6)	40 128 (98.3)	0.029	0.075
Ever	1420 (1.9)	533 (1.5)	386 (0.9)	−0.026	−0.079
Missing	732 (1.0)	296 (0.9)	315 (0.8)	−0.012	−0.021
Medicaid enrollment	8082 (10.7)	3746 (10.8)	4575 (11.2)	0.003	0.018
Pregnancy delivered at Kaiser Permanente Northern California hospital	71 916 (94.8)	33 113 (95.2)	38 891 (95.3)	0.015	0.019

Abbreviations: NA, not available; T1, birth delivery between July 1, 2018, and February 29, 2020; T2, birth delivery between March 1, 2020, and December 5, 2020; T3, birth delivery between December 6, 2020, and October 31, 2021.

^a^ Prepregnancy body mass index (calculated as weight in kilograms divided by height in meters squared) was categorized according to the World Health Organization recommendations on specific body mass index cutoffs for individuals of different races and ethnicities: non-Asian women (underweight, <18.5; normal weight, 18.5-24.9; overweight, 25.0-29.9; obese, 30.0); and Asian women (underweight, <18.5; normal weight, 18.5-22.9; overweight, 23.0-27.4; and obese, ≥27.5).^20^

### Prenatal Visits and Care Process

**Figure 1.**
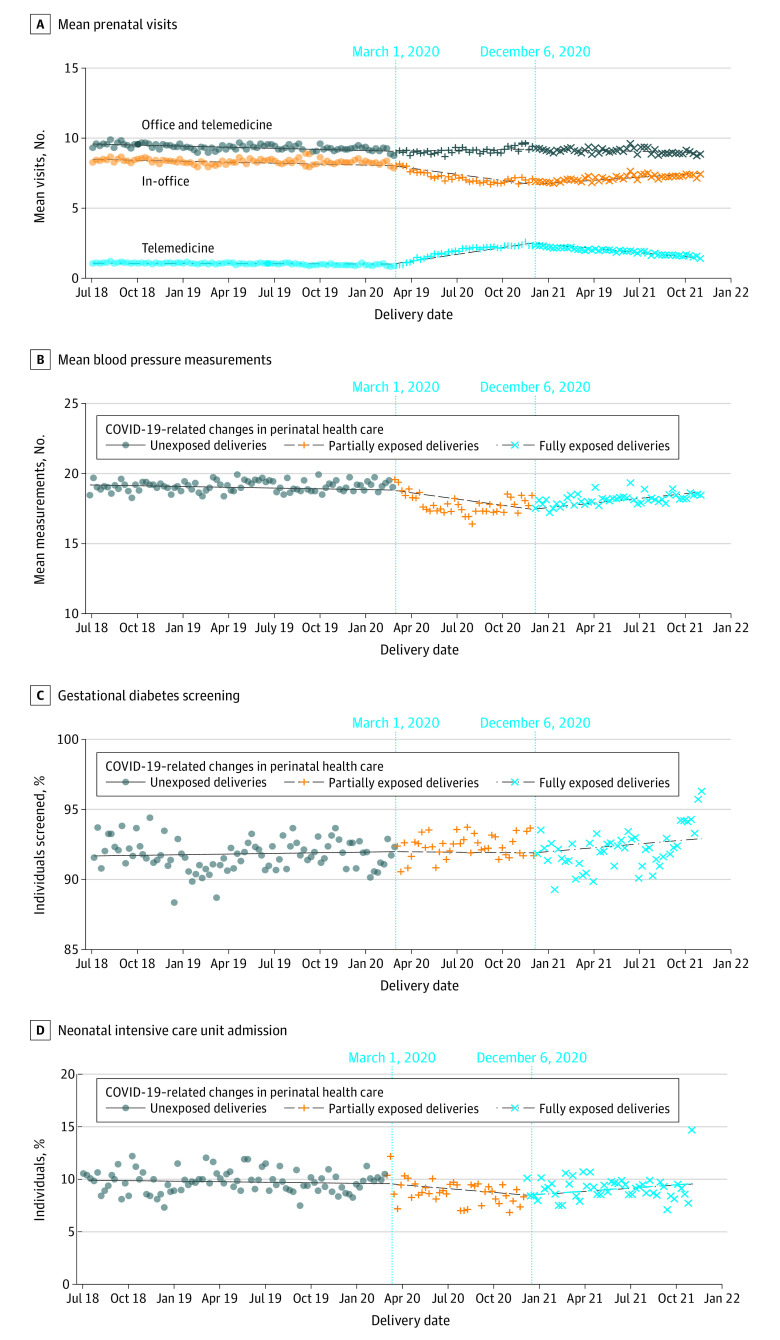
Association of Health Care Outcomes With Exposure to a Multimodal Prenatal Health Care Model During the COVID-19 Pandemic The figure shows mean prenatal visits per individual per week of birth delivery (A), mean blood pressure measurements per individual per week of birth delivery (B), the percentage of individuals screened for gestational diabetes per week of birth delivery (C), and rates of neonatal intensive care unit admission (D) among 151 464 individuals. Vertical lines indicate periods of exposure to the multimodal health care model. In the first period, individuals with birth delivery between July 1, 2018, and February 29, 2020, were unexposed to the model; in the second period, individuals with birth delivery between March 1, 2020, and December 5, 2020, were partially exposed to the model; and in the third period, individuals with birth delivery between December 6, 2020, and October 31, 2021, were fully exposed to the model.

Pregnant individuals had a similar mean (SD) number of prenatal health care visits across the prepandemic era (T1, 9.41 [4.75] visits) and the pandemic era (T2, 9.17 [4.50] visits; SMD T2 vs T1, −0.052; T3, 9.15 [4.46] visits; SMD T3 vs T1, −0.056). However, the mean (SD) number of prenatal visits by telemedicine per individual increased across the 3 intervals (T1, 1.04 [1.29] telemedicine visits; T2, 1.92 [1.87] telemedicine visits; SMD T2 vs T1, 0.543; T3, 1.95 [1.87] visits; SMD T3 vs T1, 0.563) resulting in an increase of proportion of prenatal visits by telemedicine across the 3 intervals (T1, 11.1% [79 214 visits]; T2, 20.9% [66 726 visits]; SMD T2 vs T1, 0.618; T3, 21.3% [79 518 visits]; SMD T3 vs T1, 0.649) (Table 2). The majority of telemedicine visits were performed by telephone and both the mean number of telephone visits and video conferencing visits increased across the 3 intervals (Table 2). ITS analysis showed that there was no change in the mean number of telemedicine visits per individual during T1 (change per 4-week interval, −0.05%; 95% CI, −0.14% to 0.04%) followed by an increase during T2 (change per 4-week interval, 9.75%; 95% CI, 9.49% to 10.00%) and a decrease during T3 (change per 4-week interval, −13.10%; 95% CI, −13.40% to −12.90%) (Figure 1 and Table 3).

**Table 2.** Prenatal Visits, Care Processes, and Health Outcomes of Individuals Who Delivered a Singleton Live Birth or Stillbirth by Exposure to a Multimodal Prenatal Health Care Model During the COVID-19 Pandemic at Kaiser Permanente Northern California, 2018-2021

Visit, process, or care outcome	Patients, No. (%) (N = 151 464)			Standardized mean difference	
	Unexposed (T1) (n = 75 836)	Partially exposed (T2) (n = 34 799)	Fully exposed (T3) (n = 40 829)	T2 vs T1	T3 vs T1
Prenatal visits per individual, mean (SD)					
Total	9.41 (4.8)	9.17 (4.5)	9.15 (4.5)	−0.052	−0.056
In-office	8.36 (4.2)	7.25 (3.6)	7.20 (3.5)	−0.285	−0.299
Telemedicine					
Any	1.04 (1.3)	1.92 (1.9)	1.95 (1.9)	0.543	0.563
Video	0.01 (0.1)	0.39 (0.8)	0.75 (1.1)	0.657	0.954
Telephone	1.04 (1.3)	1.53 (1.7)	1.19 (1.5)	0.332	0.114
Prenatal telemedicine visits/total visits	79 214 (11.1)	66 726 (20.9)	79 518 (21.3)	0.618	0.649
Prenatal in-office visits/total visits	634 280 (88.9)	252 258 (79.1)	294 147 (78.7)	−0.618	−0.649
Prenatal care process					
Blood pressure measurements per individual, mean (SD)	18.50 (11.4)	17.22 (11.0)	17.58 (11.3)	−0.114	−0.081
Gestational diabetes screening^a^	69 612 (91.8)	32 153 (92.4)	37 624 (92.2)	0.022	0.013
Depression screening^b^	66 379 (87.5)	29 906 (85.9)	35 787 (87.7)	−0.047	0.004
Primary prenatal maternal outcomes					
Preeclampsia and eclampsia	4982 (6.6)	2547 (7.3)	3056 (7.5)	0.029	0.036
Severe maternal morbidity^c^	2714 (3.6)	1312 (3.8)	1720 (4.2)	0.010	0.033
Cesarean delivery					
Yes	18 568 (24.5)	8911 (25.6)	10 377 (25.4)	0.026	0.022
No	53 227 (70.2)	24 151 (69.4)	28 436 (69.6)	−0.017	−0.012
Missing	4041 (5.3)	1737 (5.0)	2016 (4.9)	−0.015	−0.018
Preterm birth	6055 (8.0)	2798 (8.0)	3331 (8.2)	0.002	0.006
Spontaneous	3321 (4.4)	1427 (4.1)	1705 (4.2)	−0.014	−0.01
Medically indicated	2734 (3.6)	1371 (3.9)	1626 (4.0)	0.018	0.020
Primary newborn outcome: neonatal intensive care unit admission					
Yes	7014 (9.2)	2905 (8.3)	3516 (8.6)	−0.032	−0.022
No	64 699 (85.3)	30 144 (86.6)	35 265 (86.4)	0.038	0.03
Missing	4123 (5.4)	1750 (5.0)	2048 (5.0)	−0.018	−0.019
Secondary prenatal maternal outcomes					
Gestational hypertension	6140 (8.1)	3200 (9.2)	4164 (10.2)	0.039	0.073
Gestational diabetes					
Yes	7566 (10.0)	3280 (9.4)	3814 (9.3)	−0.019	−0.022
No	64 804 (85.5)	30 036 (86.3)	35 254 (86.3)	0.025	0.026
Missing	3466 (4.6)	1483 (4.3)	1761 (4.3)	−0.015	−0.012
Diagnosed depression	13 615 (18.0)	7205 (20.7)	9463 (23.2)	0.070	0.130
Moderate to severe depression^d^	7487 (9.9)	3106 (8.9)	3778 (9.3)	−0.032	−0.021
Venous thromboembolism	270 (0.4)	119 (0.3)	136 (0.3)	−0.002	−0.004
Secondary newborn outcomes					
Transient tachypnea	270 (0.4)	102 (0.3)	222 (0.5)	−0.011	0.028
5-min Apgar score <7					
Yes	851 (1.1)	368 (1.1)	531 (1.3)	−0.006	0.016
No	70 850 (93.4)	32 678 (93.9)	38 234 (93.6)	0.020	0.009
Missing	4135 (5.5)	1753 (5.0)	2064 (5.1)	−0.019	−0.018
Birth weight small for gestational age					
Yes	6997 (9.2)	3273 (9.4)	3698 (9.1)	0.006	−0.006
No	64 692 (85.3)	29 759 (85.5)	35 064 (85.9)	0.006	0.016
Missing	4147 (5.5)	1767 (5.1)	2067 (5.1)	−0.017	−0.018
Birth weight large for gestational age					
Yes	8447 (11.1)	3898 (11.2)	4680 (11.5)	0.002	0.010
No	63 242 (83.4)	29 134 (83.7)	34 082 (83.5)	0.009	0.002
Missing	4147 (5.5)	1767 (5.1)	2067 (5.1)	−0.017	−0.018

Abbreviations: T1, birth delivery between July 1, 2018, and February 29, 2020; T2, birth delivery between March 1, 2020, and December 5, 2020; T3, birth delivery between December 6, 2020, and October 31, 2021.

^a^ Screening was done by 50-g, 1-hour oral glucose challenge.

^b^ Screening was done with the Patient Health Questionnaire–9.

^c^ Severe maternal morbidity includes at least 1 of the following morbidities occurring at any time during pregnancy: acute myocardial infarction, aneurysm, acute kidney failure, acute respiratory distress syndrome, amniotic fluid embolism, cardiac arrest or ventricular fibrillation, conversion of cardiac rhythm, disseminated intravascular coagulation, eclampsia, heart failure or arrest during surgery or procedure, puerperal cerebrovascular disorders, pulmonary edema or acute heart failure, severe anesthesia complications, sepsis, shock, sickle cell disease with crisis, air and thrombotic embolism, blood products transfusion, hysterectomy, temporary tracheostomy, or ventilation.^9,10,11^

^d^ Defined as a Patient Health Questionnaire–9 screening score greater than or equal to 10.^15^

**Table 3.** Interrupted Time-Series Analysis for Association of Prenatal Visits, Care Processes, and Health Outcomes With Exposure to Implementation of a Multimodal Prenatal Health Care Model Among Individuals Who Delivered a Singleton Live Birth or Stillbirth at Kaiser Permanente Northern California, 2018-2021^a^

Visit, care process, or health outcome	Exposure to multimodal prenatal health care model, change per 4 wk, % (95% CI)^b^						*P* value^c^		
	Unexposed (T1)		Partially exposed (T2)		Fully exposed (T3)		T2 vs T1	T3 vs T1	T3 vs T2
	Unadjusted	Adjusted^d^	Unadjusted	Adjusted^d^	Unadjusted	Adjusted^d^			
Prenatal care visits per individual									
Total	−0.27 (−0.30 to −0.24)	−0.28 (−0.31 to −0.25)	0.45 (0.36 to 0.54)	0.48 (0.39 to 0.57)	−0.45 (−0.58 to −0.33)	−0.52 (−0.65 to −0.40)	<.001	<.001	<.001
In-office	−0.25 (−0.29 to −0.22)	−0.26 (−0.30 to −0.23)	−1.58 (−1.68 to −1.48)	−1.55 (−1.65 to −1.45)	2.75 (2.61 to 2.90)	2.69 (2.55 to 2.84)	<.001	<.001	<.001
Telemedicine									
Any	−0.01 (−0.11 to 0.08)	−0.05 (−0.14 to 0.04)	9.67 (9.42 to 9.93)	9.75 (9.49 to 10.00)	−13.00 (−13.20 to −12.70)	−13.10 (−13.40 to −12.90)	<.001	<.001	<.001
Video	16.29 (15.20 to 17.42)	16.18 (15.10 to 17.31)	18.90 (17.31 to 20.50)	19.16 (17.56 to 20.76)	−32.00 (−32.50 to −31.50)	−32.2 (−32.7 to −31.7)	<.001	<.001	<.001
Telephone	0.10 (0.01 to 0.20)	0.08 (−0.02 to 0.17)	4.42 (4.16 to 4.68)	4.45 (4.19 to 4.71)	−8.86 (−9.17 to −8.56)	−8.98 (−9.28 to −8.67)	<.001	<.001	<.001
Prenatal care process									
Blood pressure measurements per individual	−0.07 (−0.10 to −0.05)	−0.10 (−0.13 to −0.08)	−0.71 (−0.78 to −0.65)	−0.68 (−0.74 to −0.61)	1.39 (1.30 to 1.48)	1.32 (1.23 to 1.41)	<.001	<.001	<.001
Gestational diabetes screening^e^	0.01 (−0.02 to 0.04)	0.01 (−0.01 to 0.04)	−0.02 (−0.10 to 0.06)	−0.03 (−0.10 to 0.05)	0.07 (−0.04 to 0.19)	0.06 (−0.05 to 0.17)	.16	.27	.07
Depression screening^f^	−0.02 (−0.05 to 0.02)	−0.01 (−0.05 to 0.02)	−0.26 (−0.36 to −0.15)	−0.27 (−0.37 to −0.16)	0.77 (0.63 to 0.92)	0.78 (0.64 to 0.93)	<.001	<.001	<.001
Primary prenatal maternal outcomes									
Preeclampsia and eclampsia	0.80 (0.43 to 1.18)	0.76 (0.39 to 1.14)	−0.24 (−1.24 to 0.76)	−0.19 (−1.19 to 0.81)	−0.75 (−2.08 to 0.60)	−0.80 (−2.13 to 0.55)	.01	.002	.32
Severe maternal morbidity^g^	0.15 (−0.36 to 0.67)	0.12 (−0.40 to 0.63)	0.33 (−1.06 to 1.74)	0.39 (−1.00 to 1.80)	1.08 (−0.80 to 2.99)	0.99 (−0.88 to 2.90)	.61	.21	.48
Cesarean delivery	0.15 (−0.02 to 0.32)	0.06 (−0.11 to 0.23)	−0.06 (−0.53 to 0.41)	−0.03 (−0.49 to 0.44)	0.06 (−0.59 to 0.71)	−0.05 (−0.68 to 0.59)	.62	.65	.94
Preterm birth									
Any	0.29 (−0.05 to 0.63)	0.23 (−0.11 to 0.57)	−0.46 (−1.37 to 0.47)	−0.37 (−1.29 to 0.55)	0.00 (−1.27 to 1.29)	−0.15 (−1.41 to 1.13)	.09	.43	.70
Spontaneous	−0.16 (−0.62 to 0.31)	−0.18 (−0.65 to 0.28)	−0.37 (−1.66 to 0.93)	−0.33 (−1.62 to 0.97)	0.46 (−1.35 to 2.32)	0.40 (−1.42 to 2.25)	.76	.39	.36
Medically indicated	0.84 (0.33 to 1.36)	0.73 (0.22 to 1.24)	−0.63 (−1.99 to 0.75)	−0.50 (−1.86 to 0.88)	−0.46 (−2.31 to 1.43)	−0.69 (−2.53 to 1.18)	.02	.04	.81
Primary newborn outcome: neonatal intensive care unit admission	−0.17 (−0.48 to 0.15)	−0.22 (−0.53 to 0.09)	−0.95 (−1.82 to −0.08)	−0.91 (−1.77 to −0.03)	1.75 (0.49 to 3.02)	1.63 (0.38 to 2.89)	.04	<.001	<.001
Secondary prenatal maternal outcomes									
Gestational hypertension	0.84 (0.51 to 1.18)	0.76 (0.43 to 1.10)	0.42 (−0.46 to 1.31)	0.53 (−0.35 to 1.42)	−1.13 (−2.28 to 0.03)	−1.30 (−2.44 to −0.15)	.50	<.001	<.001
Gestational diabetes	−0.54 (−0.83 to −0.24)	−0.74 (−1.03 to −0.46)	0.68 (−0.16 to 1.53)	0.93 (0.12 to 1.76)	−0.00 (−1.16 to 1.17)	−0.47 (−1.60 to 0.68)	<.001	.51	<.01
Diagnosed depression	0.71 (0.50 to 0.92)	0.70 (0.49 to 0.91)	0.72 (0.17 to 1.28)	0.70 (0.16 to 1.25)	−0.82 (−1.53 to −0.10)	−0.89 (−1.59 to −0.19)	>.99	<.001	<.001
Moderate to severe depression^h^	0.21 (−0.09 to 0.51)	0.18 (−0.11 to 0.48)	−1.71 (−2.54 to −0.88)	−1.68 (−2.50 to −0.85)	2.53 (1.32 to 3.76)	2.53 (1.33 to 3.75)	<.001	<.001	<.001
Venous thromboembolism	−0.14 (−1.78 to 1.53)	−0.25 (−1.89 to 1.42)	−1.77 (−6.29 to 2.98)	−1.67 (−6.20 to 3.08)	4.58 (−2.05 to 11.77)	4.36 (−2.31 to 11.48)	.43	.07	.04
Secondary newborn outcomes									
Transient tachypnea	−1.27 (−2.91 to 0.40)	−1.28 (−2.92 to 0.39)	2.59 (−2.05 to 7.49)	2.56 (−2.10 to 7.44)	5.65 (−0.39 to 12.17)	5.61 (−0.48 to 12.06)	.03	.002	.28
5-min Apgar score <7	−0.50 (−1.42 to 0.42)	−0.57 (−1.48 to 0.36)	1.13 (−1.44 to 3.77)	1.20 (−1.37 to 3.85)	1.10 (−2.33 to 4.69)	0.98 (−2.46 to 4.55)	.07	.23	.89
Birth weight small for gestational age	0.08 (−0.23 to 0.39)	0.07 (−0.23 to 0.38)	0.19 (−0.67 to 1.05)	0.34 (−0.50 to 1.20)	−1.03 (−2.19 to 0.16)	−1.10 (−2.26 to 0.06)	.41	.007	.005
Birth weight large for gestational age	−0.10 (−0.38 to 0.17)	−0.19 (−0.46 to 0.09)	0.35 (−0.42 to 1.13)	0.39 (−0.37 to 1.16)	−0.21 (−1.26 to 0.85)	−0.35 (−1.39 to 0.69)	.05	.67	.11

Abbreviations: T1, birth delivery between July 1, 2018, and February 29, 2020; T2, birth delivery between March 1, 2020, and December 5, 2020; T3, birth delivery between December 6, 2020, and October 31, 2021.

^a^ Interrupted time series method for prenatal outcomes before and during the implementation of COVID-19–related changes in prenatal health care used piecewise linear segmented regression, allowing a change in slope associated with time at 2 inflection points: March 1, 2020 (initiation of changes in prenatal health care), and December 6, 2020 (date when pregnancies were fully exposed to the prenatal health care changes). Log binomial regression was used to examine each binary outcome in relation to date of birth delivery (week), providing point and interval estimates of relative percentage change in outcome probability associated with change in time, scaled to a 4-week change. Poisson regression was used for point and interval estimation of relative percentage change in means for outcomes characterized as counts in relation to a 4-week change in birth delivery date.

^b^ Individuals with delivery in T2 did not experience the multimodal prenatal health care model implemented during COVID-19 pandemic for the entire duration of the pregnancy. Individuals with delivery in T3 experienced the multimodal prenatal health care model for the entire duration of the pregnancy.

^c^ *P* values were calculated with 2-sided Wald test adjusted for age, prepregnancy body mass index, neighborhood deprivation index, and race and ethnicity.

^d^ Adjusted for age, prepregnancy body mass index, neighborhood deprivation index, and race and ethnicity.

^e^ Screening was performed by 50-g, 1-hour oral glucose challenge test.

^f^ Screening was performed with the Patient Health Questionnaire–9.

^g^ Severe maternal morbidity includes at least 1 of the following morbidities occurring at any time during pregnancy: acute myocardial infarction, aneurysm, acute kidney failure, acute respiratory distress syndrome, amniotic fluid embolism, cardiac arrest or ventricular fibrillation, conversion of cardiac rhythm, disseminated intravascular coagulation, eclampsia, heart failure or arrest during surgery or procedure, puerperal cerebrovascular disorders, pulmonary edema or acute heart failure, severe anesthesia complications, sepsis, shock, sickle cell disease with crisis, air and thrombotic embolism, blood products transfusion, hysterectomy, temporary tracheostomy, or ventilation.^9,10,11^

^h^ Defined as a Patient Health Questionnaire–9 screening score greater than or equal to 10.^15^

There were no clinically notable differences in the mean number of blood pressure measurements, rates of GD, or depression screenings across the 3 time intervals (Table 2). ITS analysis found that there were decreases in the mean number of blood pressure measurements per individual during T1 (change per 4-week interval, −0.10%; 95% CI, −0.13% to −0.08%) and T2 (change per 4-week interval, −0.68%; 95% CI, −0.74% to −0.61%) that were followed by an increase during T3 (change per 4-week interval, 1.32%; 95% CI, 1.23% to 1.41%) (Figure 1 and Table 3). The screening rate for depression was stable during T1, followed by a decrease during T2 (change per 4-week interval, −0.27%; 95% CI, −0.37% to −0.16%) and an increase during T3 (change per 4-week interval, 0.78%; 95% CI, 0.64% to 0.93%) (eFigure 1 in Supplement 1). There were no changes across the 3 intervals for the GD screenings (Table 3 and Figure 1). Similar outcomes in prenatal visits (totals and by modality) and care processes were observed across individuals of different races and ethnicities, among individuals in the highest NDI quartile, among individuals who preferred a language other than English, among those living in a rural area, and those with a low-risk pregnancy (eTables 2-13 in Supplement 1).

### Primary Health Outcomes

As shown in Table 2, rates of primary health outcomes were similar across the 3 intervals. Small changes were observed for rates of NICU admission, which decreased from T1 (7014 admissions [9.2%]) to T2 (2905 admissions [8.3%]; SMD T2 vs T1, –0.032) and increased in T3 (3516 admissions [8.6%]; SMD T3 vs T1, –0.022).

**Figure 2.**
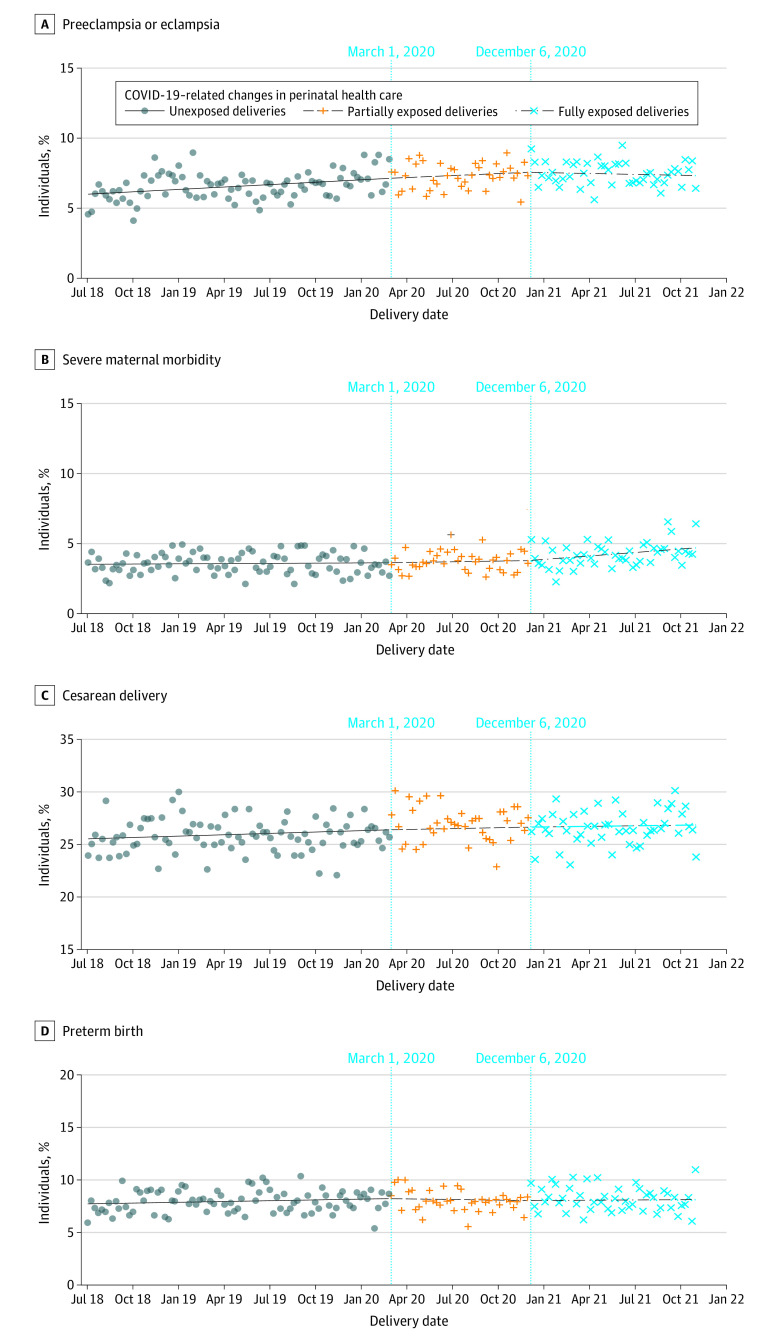
Associations of Prenatal Health Outcomes With a Multimodal Prenatal Health Care Model During the COVID-19 Pandemic The figure shows rates of preeclampsia and eclampsia (A), severe maternal morbidity (B), cesarean delivery (C), and preterm birth (D) among 151 464 individuals. Vertical lines indicate periods of exposure to the multimodal health care model. In the first period, individuals with birth delivery between July 1, 2018, and February 29, 2020, were unexposed to the model; in the second period, individuals with birth delivery between March 1, 2020, and December 5, 2020, were partially exposed to the model; and in the third period, individuals with birth delivery between December 6, 2020, and October 31, 2021, were fully exposed to the model.

ITS analysis showed no change in risk for NICU admission during T1 (change per 4-week interval,−0.22%; 95% CI, –0.53% to 0.09%), followed by a decrease in risk during T2 (change per 4-week interval, −0.91%; 95% CI, –1.77% to −0.03%), and an increase during T3 (change per 4-week interval, 1.75%; 95% CI, 0.49% to 3.02%) (Figure 1 and Table 3). ITS analysis showed no changes during T1, T2, and T3 for the risk of preeclampsia and eclampsia (change per 4-week interval, 0.76% [95% CI, 0.39% to 1.14%] for T1; −0.19% [95% CI, –1.19% to 0.81%] for T2; and −0.80% [95% CI, –2.13% to 0.55%] for T3), severe maternal morbidity (change per 4-week interval, 0.12% [95% CI, –0.40% to 0.63%] for T1; 0.39 [95% CI, –1.00% to 1.80%] for T2; and 0.99% [95% CI, –0.88% to 2.90%] for T3), cesarean delivery (change per 4-week interval, 0.06% [95% CI, –0.11% to 0.23%] for T1; −0.03% [95% CI, –0.49% to 0.44%] for T2; and −0.05%; [95% CI, –0.68% to 0.59%] for T3), and preterm birth (change per 4-week interval, 0.23% [95% CI, –0.11% to 0.57%] for T1; −0.37%; [95% CI, –1.29% to 0.55%] for T2; and −0.15% [95% CI, –1.41% to 1.13%] for T3), nor spontaneous or medically indicated preterm birth subtypes (Figure 2).

### Secondary Health Outcomes

Rates of gestational hypertension, GD, and depression slightly increased from T1 to T2 and from T2 to T3 (Table 2). ITS analysis (eFigure 2 in Supplement 1 and Table 3) showed an increase in gestational hypertension during T1 (change per 4-week interval, 0.76%; 95% CI, 0.43% to 1.10%) with no change in T2 (change per 4-week interval, 0.53%; 95% CI, –0.35% to 1.42%) followed by a decrease in risk in T3 (change per 4-week interval, −1.30%; 95% CI, –2.44% to −0.15%). For GD, there was a decrease in GD risk during T1 (change per 4-week interval, −0.74%; 95% CI, –1.03% to −0.46%), which changed toward an increase in risk during T2 (change per 4-week interval, 0.93%; 95% CI, 0.12% to 1.76%) and a leveling off during T3 (change per 4-week time interval, −0.47%; 95% CI, –1.60% to 0.68%). For depression, there was an increase in risk during T1 (change per 4-week interval, 0.70%; 95% CI, 0.49 to 0.91), which continued at similar rate in T2 (change per 4-week interval, 0.70%; 95% CI, 0.16% to 1.25%), followed by a decrease in risk during T3 (change per 4-week interval, −0.89%; 95% CI, –1.59% to −0.19%). There were no changes during the 3 intervals in the rates of risk of moderate-to-severe depression, VTE, transient tachypnea, a 5-minute Apgar score of less than 7, SGA, and LGA.

Overall, similar rates of prenatal visits and care processes and primary and secondary health outcomes were found among individuals in the highest NDI quartile, among those who preferred a language other than English, lived in a rural area, had a low-risk pregnancy, and among individuals of different races and ethnicities, with a few exceptions (eTables 2-13 in Supplement 1). Among Black individuals, there was an increase in risk of severe maternal morbidity during T1 (change per 4-week interval, 2.30%; 95% CI, 0.59% to 4.04%), followed by a decrease in T2 (change per 4-week interval, −6.50%; 95% CI, –10.70% to −2.15%) and an increase in T3 (change per 4-week interval, 8.96%; 95% CI, 2.31% to 16.03%) (eTable 9 in Supplement 1). Among Asian and Pacific Islander individuals, during T2 there was increased risk for severe maternal morbidity (change per 4-week interval, 3.22%; 95% CI, 0.53% to 5.97%) and SGA (change per 4-week interval, 1.64%; 95% CI, 0.27% to 3.03%) which were followed by a decrease in risk for severe maternal morbidity (change per 4-week interval, −2.30%; 95% CI, –5.67% to 1.19%) and SGA (change per 4-week interval, −2.38%; 95% CI, –4.19% to −0.54%) during T3; whereas for a 5-minute Apgar score of less than 7, there was a decrease in risk during T2 (change per 4-week interval, −5.65%; 95% CI, –10.70% to −0.29%) followed by an increase during T3 (change per 4-week time interval, 12.82%; 95% CI, 4.48% to 21.82%) (eTable 8 in Supplement 1).

## Discussion

In this cohort study in an integrated health care delivery system with prepaid insurance, after implementing a multimodal prenatal health care model with use of both in-office and telemedicine visits during the COVID-19 pandemic, the mean number of prenatal visits via telemedicine increased to 21.3% of the total visits per individual compared with 11.1% of total visits during the prepandemic era. There were no clinically significant differences in prenatal health care process measures such as blood pressure measurements, GD, or depression screenings across pandemic periods (T2 and T3). Most importantly, there were no clinically significant changes in most of our primary health outcomes, including preeclampsia and eclampsia, severe maternal morbidity, cesarean delivery, and preterm birth, with the exception of NICU admission rates. Although NICU admission rates were stable from the prepandemic period to the pandemic period, ITS analysis showed a decrease in risk during the first pandemic period (T2) and an increase during the second pandemic period (T3) when individuals were fully exposed to the multimodal prenatal health care model. Possible reasons for this increase in the risk of NICU admission rates require further investigation. Regarding secondary health outcomes, although the rates of gestational hypertension and depression increased from the prepandemic era through the pandemic era, ITS analysis showed that there were already increases in the risk of these conditions during the prepandemic period. Our results were consistent with a study^25^ conducted among 22 323 pregnant individuals in Australia that used ITS analysis to compare usual prepandemic health care with a hybrid prenatal health care model implemented during the COVID-19 pandemic that resulted in 50% of visits being conducted via telemedicine. The study^25^ found no differences in the weekly rates of GD, preeclampsia, preterm birth, or NICU admission rates between the 2 study periods. Our results were also consistent with 2 studies^26,27^ conducted in the US with smaller numbers (ie, 12 607 and 1894 pregnant individuals) that used a before-and-after design to compare individuals who delivered during the COVID-19 pandemic and received a hybrid health care model of in-office and telemedicine with individuals who delivered before the pandemic and received in-office prenatal visits only. Rates of preeclampsia and cesarean delivery did not differ between groups.^26,27^ However, none of these studies assessed whether performance of these hybrid health care models was similar across sociodemographic factors.

It is worth noting we observed similar results among individuals living in areas with a low NDI index, who preferred a language other than English, or were living in a rural area. Similar results were also obtained among individuals of different races and ethnicities, with a few exceptions. The most relevant differences compared with the whole cohort were those that showed an increase in the risk among those fully exposed to the multimodal health care model (ie, during T3) such as increases in the risk of severe maternal morbidity among Black individuals and increases in the risk of a 5-minute Apgar score of less than 7 among Asian and Pacific Islander individuals. These findings need further investigation to understand whether they were directly associated with the multimodal prenatal health care model or exposure to inequitable conditions within or outside of the health care system, or whether they might be chance findings in the context of the large number of regression analyses we performed.

Prenatal health care delivery is currently under evaluation and redesign as a result of the COVID-19 pandemic.^1^ Integrating the use of telemedicine in prenatal health care provides an alternative to exclusive in-office care for populations living in underserved areas or facing access barriers and has received both promising attention and some concern.^2^ Although populations living in underserved areas are also at risk for health disparities due to less access to technology and possible language barriers, as shown in the general population,^28,29,30^ we did not observe differences in the uptake of telemedicine according to sociodemographic characteristics, and qualitative assessments of telemedicine programs show that pregnant individuals and their health care clinicians in rural or urban regions of the US perceive telemedicine as a positive experience.^2,31,32,33,34^

### Strengths and Limitations

Strengths of this study are the cohort design, inclusion of a large number of pregnant individuals and newborns, the ability to ascertain prenatal outcomes using robust longitudinal clinical data, the representativeness of the study sample and its racial and ethnic diversity, and use of ITS analysis, a rigorous study design for evaluating the outcomes of natural experiments, which accounted for partial and full exposure to the multimodal prenatal health care model implemented during the COVID-19 pandemic. Another strength of the study was its ability to assess the uptake and outcomes of the multimodal prenatal health care model by several sociodemographic factors.

Limitations of this study include different levels of exposure to the multimodal prenatal health care model among individuals who delivered during the first pandemic period (T2); therefore, analyses of some outcomes were subject to possible exposure misclassification. However, analyses of outcomes during the second pandemic period (T3), when pregnancies were homogeneously and fully exposed to the multimodal prenatal health care model, were subject to no exposure misclassification. During the first pandemic period (T2), lockdown policies and reduced individual comfort levels with being in person may also have affected complete assessment of the health outcomes. We were not able to accurately define pregnancies at low-risk or with specific moderate-risk conditions, since these definitions also include clinician discretion, for which we do not have complete documentation. Furthermore, we did not have data on patients’ and health care clinicians’ satisfaction with telemedicine.

This study has several characteristics that ensure the generalizability of its results in similar health care settings given the representativeness of the KPNC pregnant population,^4,5,6^ which was also racially and geographically diverse. Although prenatal health care is covered without cost sharing under the Patient Protection and Affordable Care Act,^35^ policies for coverage of telemedicine during pregnancy have not been implemented^36,37^; therefore, our results maybe not generalizable to all health care settings.

## Conclusions

The findings of our study suggest adequate performance of a multimodal health care model including in-office and telemedicine prenatal health care during the COVID-19 pandemic compared with traditional in-office prenatal health care used before the-pandemic. Additionally, the performance was similar across several sociodemographic factors, supporting continued use of multimodal health care delivery going forward.

## Notes

Supplement 1. **eAppendix.** eMethods **eTable 1.** *ICD-10* Diagnostic Codes **eFigure 1.** Associations of Prenatal Depression Screening With a Multimodal Prenatal Healthcare Model During the COVID-19 Pandemic **eFigure 2.** Associations of Prenatal Secondary Health Outcomes With a Multimodal Prenatal Healthcare Model During the COVID-19 Pandemic **eTable 2.** Interrupted Time Series Analysis for the Trends in Prenatal Visits, Care Process, and Health Outcomes in Association With Exposure to COVID-19 Pandemic–Related Implementation of a Multimodal Prenatal Health Care Delivery Among 36 167 Individuals Who Were in the Highest Quartile of the Neighborhood Deprivation Index and Delivered a Singleton Live Birth or Stillbirth, Kaiser Permanente Northern California, 2018-2021 **eTable 3.** Interrupted Time Series Analysis for the Trends in Prenatal Visits, Care Process, and Health Outcomes in Association With Exposure to COVID-19 Pandemic–Related Implementation of a Multimodal Prenatal Health Care Delivery Among 113 602 Individuals Who Were in the First, Second or Third Quartile of the Neighborhood Deprivation Index and Delivered a Singleton Live Birth or Stillbirth, Kaiser Permanente Northern California, 2018-2021 **eTable 4.** Interrupted Time Series Analysis for the Trends in Prenatal Visits, Care Process, and Health Outcomes in Association With Exposure to COVID-19 Pandemic–Related Implementation of a Multimodal Prenatal Health Care Delivery Among 141 683 Individuals Who Preferred English Language and Delivered a Singleton Live Birth or Stillbirth, Kaiser Permanente, Northern California, 2018-2021 **eTable 5.** Interrupted Time Series Analysis for the Trends in Prenatal Visits, Care Process, and Health Outcomes in Association With Exposure to COVID-19 Pandemic–Related Implementation of a Multimodal Prenatal Health Care Delivery Among 9781 Individuals Who Did Not Prefer English Language and Delivered a Singleton Live Birth or Stillbirth, Kaiser Permanente Northern California, 2018-2021 **eTable 6.** Interrupted Time Series Analysis for the Trends in Prenatal Visits, Care Process, and Health Outcomes in Association With Exposure to COVID-19 Pandemic–Related Implementation of a Multimodal Prenatal Health Care Delivery Among 145 940 Individuals Who Lived in Urban Areas and Delivered a Singleton Live Birth or Stillbirth, Kaiser Permanente Northern California, 2018-2021 **eTable 7.** Interrupted Time Series Analysis for the Trends in Prenatal Visits, Care Process, and Health Outcomes in Association With Exposure to COVID-19 Pandemic–Related Implementation of a Multimodal Prenatal Health Care Delivery Among 5305 Individuals Who Lived in Rural Areas and Delivered a Singleton Live Birth or Stillbirth, Kaiser Permanente Northern California, 2018-2021 **eTable 8.** Interrupted Time Series Analysis for the Trends in Prenatal Visits, Care Process, and Health Outcomes in Association With Exposure to COVID-19 Pandemic–Related Implementation of a Multimodal Prenatal Health Care Delivery Among 39 114 Asian and Pacific Islander Individuals Who Delivered a Singleton Live Birth or Stillbirth, Kaiser Permanente Northern California, 2018-2021 **eTable 9.** Interrupted Time Series Analysis for the Trends in Prenatal Visits, Care Process, and Health Outcomes in Association With Exposure to COVID-19 Pandemic–Related Implementation of a Multimodal Prenatal Health Care Delivery Among 10 013 Black Individuals Who Delivered a Singleton Live Birth or Stillbirth, Kaiser Permanente Northern California, 2018-2021 **eTable 10.** Interrupted Time Series Analysis for the Trends in Prenatal Visits, Care Process, and Health Outcomes in Association With Exposure to COVID-19 Pandemic–Related Implementation of a Multimodal Prenatal Health Care Delivery Among 42 245 Hispanic Individuals Who Delivered a Singleton Live Birth or Stillbirth, Kaiser Permanente Northern California, 2018-2021 **eTable 11.** Interrupted Time Series Analysis for the Trends in Prenatal Visits, Care Process, and Health Outcomes in Association With Exposure to COVID-19 Pandemic–Related Implementation of a Multimodal Prenatal Health Care Delivery Among 52 129 White Individuals Who Delivered a Singleton Live Birth or Stillbirth, Kaiser Permanente Northern California, 2018-2021 **eTable 12.** Interrupted Time Series Analysis for the Trends in Prenatal Visits, Care Process, and Health Outcomes in Association With Exposure to COVID-19 Pandemic–Related Implementation of a Multimodal Prenatal Health Care Delivery Among 112 861 Individuals Who Had a Low-Risk Pregnancy Who Delivered a Singleton Live Birth or Stillbirth, Kaiser Permanente Northern California, 2018-2021 **eTable 13.** Interrupted Time Series Analysis for the Trends in Prenatal Visits, Care Process, and Health Outcomes in Association With Exposure to COVID-19 Pandemic–Related Implementation of a Multimodal Prenatal Health Care Delivery Among 8,603 Individuals Who Did Not Have a Low-Risk Pregnancy Who Delivered a Singleton Live Birth or Stillbirth, Kaiser Permanente Northern California, 2018-2021
eReferences

Supplement 2.
Data Sharing Statement
