# Supplementary material for: Prenatal Health Care Outcomes Before and During the COVID-19 Pandemic Among Pregnant Individuals and Their Newborns in an Integrated US Health System
Source: JAMA Netw Open. 2023 Jul 18;6(7):e2324011. doi: 10.1001/jamanetworkopen.2023.24011 (PMC10354684; doi:10.1001/jamanetworkopen.2023.24011)
Supplement: Supplement 1. — eAppendix. eMethods eTable 1. ICD-10 Diagnostic Codes eFigure 1. Associations of Prenatal Depression Screening With a Multimodal Prenatal Healthcare Model During the COVID-19 Pandemic eFigure 2. Associations of Prenatal Secondary Health Outcomes With a Multimodal Prenatal Healthcare Model During the COVID-19 Pandemic eTable 2. Interrupted Time Series Analysis for the Trends in Prenatal Visits, Care Process, and Health Outcomes in Association With Exposure to COVID-19 Pandemic–Related Implementation of a Multimodal Prenatal Health Care Delivery Among 36 167 Individuals Who Were in the Highest Quartile of the Neighborhood Deprivation Index and Delivered a Singleton Live Birth or Stillbirth, Kaiser Permanente Northern California, 2018-2021 eTable 3. Interrupted Time Series Analysis for the Trends in Prenatal Visits, Care Process, and Health Outcomes in Association With Exposure to COVID-19 Pandemic–Related Implementation of a Multimodal Prenatal Health Care Delivery Among 113 602 Individuals Who Were in the First, Second or Third Quartile of the Neighborhood Deprivation Index and Delivered a Singleton Live Birth or Stillbirth, Kaiser Permanente Northern California, 2018-2021 eTable 4. Interrupted Time Series Analysis for the Trends in Prenatal Visits, Care Process, and Health Outcomes in Association With Exposure to COVID-19 Pandemic–Related Implementation of a Multimodal Prenatal Health Care Delivery Among 141 683 Individuals Who Preferred English Language and Delivered a Singleton Live Birth or Stillbirth, Kaiser Permanente, Northern California, 2018-2021 eTable 5. Interrupted Time Series Analysis for the Trends in Prenatal Visits, Care Process, and Health Outcomes in Association With Exposure to COVID-19 Pandemic–Related Implementation of a Multimodal Prenatal Health Care Delivery Among 9781 Individuals Who Did Not Prefer English Language and Delivered a Singleton Live Birth or Stillbirth, Kaiser Permanente Northern California, 2018-2021 eTable 6. Interrupted Time S [file jamanetwopen-e2324011-s001.pdf]

Supplemental Online Content

Ferrara A, Greenberg M, Zhu Y, et al. Prenatal health care outcomes before and during the COVID-19 pandemic among pregnant individuals and their newborns in an integrated US health system. *JAMA Netw Open*. 2023;6(7):e2324011. doi:10.1001/jamanetworkopen.2023.24011

eAppendix. eMethods

eTable 1. *ICD-10* Diagnostic Codes

eFigure 1. Associations of Prenatal Depression Screening With a Multimodal Prenatal Healthcare Model During the COVID-19 Pandemic

eFigure 2. Associations of Prenatal Secondary Health Outcomes With a Multimodal Prenatal Healthcare Model During the COVID-19 Pandemic

eTable 2. Interrupted Time Series Analysis for the Trends in Prenatal Visits, Care Process, and Health Outcomes in Association With Exposure to COVID-19 Pandemic–Related Implementation of a Multimodal Prenatal Health Care Delivery Among 36 167 Individuals Who Were in the Highest Quartile of the Neighborhood Deprivation Index and Delivered a Singleton Live Birth or Stillbirth, Kaiser Permanente Northern California, 2018-2021

eTable 3. Interrupted Time Series Analysis for the Trends in Prenatal Visits, Care Process, and Health Outcomes in Association With Exposure to COVID-19 Pandemic–Related Implementation of a Multimodal Prenatal Health Care Delivery Among 113 602 Individuals Who Were in the First, Second or Third Quartile of the Neighborhood Deprivation Index and Delivered a Singleton Live Birth or Stillbirth, Kaiser Permanente Northern California, 2018-2021

eTable 4. Interrupted Time Series Analysis for the Trends in Prenatal Visits, Care Process, and Health Outcomes in Association With Exposure to COVID-19 Pandemic–Related Implementation of a Multimodal Prenatal Health Care Delivery Among 141 683 Individuals Who Preferred English Language and Delivered a Singleton Live Birth or Stillbirth, Kaiser Permanente, Northern California, 2018-2021

eTable 5. Interrupted Time Series Analysis for the Trends in Prenatal Visits, Care Process, and Health Outcomes in Association With Exposure to COVID-19 Pandemic–Related Implementation of a Multimodal Prenatal Health Care Delivery Among 9781 Individuals Who Did Not Prefer English Language and Delivered a Singleton Live Birth or Stillbirth, Kaiser Permanente Northern California, 2018-2021

eTable 6. Interrupted Time Series Analysis for the Trends in Prenatal Visits, Care Process, and Health Outcomes in Association With Exposure to COVID-19 Pandemic–Related Implementation of a Multimodal Prenatal Health Care Delivery Among 145 940 Individuals Who Lived in Urban Areas and Delivered a Singleton Live Birth or Stillbirth, Kaiser Permanente Northern California, 2018-2021

eTable 7. Interrupted Time Series Analysis for the Trends in Prenatal Visits, Care Process, and Health Outcomes in Association With Exposure to COVID-19 Pandemic–Related Implementation of a Multimodal Prenatal Health Care Delivery Among 5305 Individuals Who

© 2023 Ferrera A et al. *JAMA Network Open.*

Lived in Rural Areas and Delivered a Singleton Live Birth or Stillbirth, Kaiser Permanente Northern California, 2018-2021

eTable 8. Interrupted Time Series Analysis for the Trends in Prenatal Visits, Care Process, and Health Outcomes in Association With Exposure to COVID-19 Pandemic–Related Implementation of a Multimodal Prenatal Health Care Delivery Among 39 114 Asian and Pacific Islander Individuals Who Delivered a Singleton Live Birth or Stillbirth, Kaiser Permanente Northern California, 2018-2021

eTable 9. Interrupted Time Series Analysis for the Trends in Prenatal Visits, Care Process, and Health Outcomes in Association With Exposure to COVID-19 Pandemic–Related Implementation of a Multimodal Prenatal Health Care Delivery Among 10 013 Black Individuals Who Delivered a Singleton Live Birth or Stillbirth, Kaiser Permanente Northern California, 2018-2021

eTable 10. Interrupted Time Series Analysis for the Trends in Prenatal Visits, Care Process, and Health Outcomes in Association With Exposure to COVID-19 Pandemic–Related Implementation of a Multimodal Prenatal Health Care Delivery Among 42 245 Hispanic Individuals Who Delivered a Singleton Live Birth or Stillbirth, Kaiser Permanente Northern California, 2018-2021

eTable 11. Interrupted Time Series Analysis for the Trends in Prenatal Visits, Care Process, and Health Outcomes in Association With Exposure to COVID-19 Pandemic–Related Implementation of a Multimodal Prenatal Health Care Delivery Among 52 129 White Individuals Who Who Delivered a Singleton Live Birth or Stillbirth, Kaiser Permanente Northern California, 2018-2021

eTable 12. Interrupted Time Series Analysis for the Trends in Prenatal Visits, Care Process, and Health Outcomes in Association With Exposure to COVID-19 Pandemic–Related Implementation of a Multimodal Prenatal Health Care Delivery Among 112 861 Individuals Who Had a Low-Risk Pregnancy Who Delivered a Singleton Live Birth or Stillbirth, Kaiser Permanente Northern California, 2018-2021

eTable 13. Interrupted Time Series Analysis for the Trends in Prenatal Visits, Care Process, and Health Outcomes in Association With Exposure to COVID-19 Pandemic–Related Implementation of a Multimodal Prenatal Health Care Delivery Among 8,603 Individuals Who Did Not Have a Low-Risk Pregnancy Who Delivered a Singleton Live Birth or Stillbirth, Kaiser Permanente Northern California, 2018-2021

eReferences

This supplemental material has been provided by the authors to give readers additional information about their work.

eMethods.

Data collection. The data were obtained from the electronic health records or administrative databases and clinical data were collected by healthcare providers.

Prenatal healthcare visits. We defined as prenatal healthcare visits those that were conducted during pregnancy (either in-person or via telemedicine) at or with healthcare providers from the departments of obstetrics and gynecology, maternal-fetal medicine/perinatology, family practice/internal medicine and reproductive and women's health.

Implementation of a multimodal prenatal healthcare delivery. As of March 13, 2020, the following changes in prenatal healthcare were implemented. For low-risk pregnancies, defined as age <40 years, or with specific moderate risk conditions (including gestational diabetes [GDM] on diet therapy only, chronic hypertension without medication management, prior cesarean delivery, and other conditions per provider discretion) the following four standard in-office prenatal healthcare visits were converted to telemedicine visits, either by video or by telephone according to patients' preference: 10-12 weeks (after completion of 1st trimester office physical and ultrasound examination), 22 weeks, 28 weeks, and 32 weeks. Prior to the telephone or video visits, patients were sent messaging describing expectations for the telemedicine visit. During the telemedicine visits, individuals were asked to check blood pressure, fundal height, and weight using at-home devices if available, and received standard screening, results review, and guidance per their stage of pregnancy. Depression screening, previously conducted in-office, was provided by virtual questionnaire. In-office visits retained on-schedule included 1st trimester intake, 16-18 weeks, 30 weeks, and all visits at 34 weeks and beyond. In-office visits continued to include essential prenatal care assessments such as fetal heart rate monitoring, routine ultrasound screening, and consolidated visits to the laboratory. Additional in-office visits could be scheduled or requested by patients and providers as deemed needed, as occurs during standard prenatal care.

Schedule was intended to be: Intake – in-office, 10-12w – telemedicine, 16w – in-office plus go to lab, 20w – in-office ultrasound, 22w – telemedicine, 26w – in-office plus go to lab, 28w – telemedicine, 30w – in-office, 32w – telemedicine, and any visit at 34w or later was in-office.

Urban/rural area. For the 2020 Census, an urban area will comprise a densely settled core of census blocks that meet minimum housing unit density and/or population density requirements. This includes adjacent territory containing non-residential urban land uses. To qualify as an urban area, the territory identified according to criteria must encompass at least 2,000 housing units or have a population of at least 5,000.[1]

Statistical analysis

Interrupted time series (ITS) design. Changes in rates of outcomes of interest in relation to changes in perinatal and postnatal health care delivery in response to the COVID-19 pandemic were assessed using a quasi-experimental interrupted time series (ITS) design.[2] ITS is a rigorous study design for evaluating the effect of population-level health interventions or natural experiments, with the aim of estimating causal effects using observational data when randomization is not possible. While this method is often applied to aggregate statistics measured at the population level (e.g. proportions, means), it can be applied using patient-level data, providing interpretation of effect estimates at the individual level, increased power and precision, and a more flexible framework for covariate adjustment.[3,4] We considered pregnancies as independent observations, with no adjustment/control for between-women correlation. Appropriate to ITS analyses with subject level data, we approached the analysis in a generalized linear model framework, accommodating the distribution types of our outcomes of interest.[4,5] Log binomial regression was used to examine each binary outcome of interest in relation to time (week of delivery) and other patient-level covariates, providing estimates of percent change in outcome probability per increment in time. Given the relatively high incidence of some outcomes of interest (e.g. cesarean delivery) this approach avoids potential misinterpretation of odds ratios obtained from the alternative logistic regression approach. Remaining outcomes of interest,

© 2023 Ferrera A et al. *JAMA Network Open.*

characterized as counts during a given pregnancy (e.g. number of office visits, number of blood pressure measurements) were analyzed using Poisson regression, providing estimates of percent change in means per increment in time.

Following standard ITS methodology, we used piecewise linear segmented regression for estimation of trends in pregnancy outcomes of interest before and during the pandemic. We specified a breakpoint on the date of initiation of the change in perinatal health care delivery (3/1/20). We reference the pre-pandemic time interval prior to 3/1/20 as T1. Pregnancies with delivery dates between 3/1/20 and 12/6/20 (interval T2) did not experience the change in health care delivery for the entire duration of the pregnancy, with the fraction of a pregnancy exposed to the change increasing from 0% to 100% with each additional day during this time interval. We specified a second breakpoint at the end of this first post-pandemic time interval (12/6/20; beginning of interval T3), the date at which all future deliveries were associated with pregnancies that experienced the health care changes for the entire duration of gestation. Change in outcome risk or mean count per each additional day in the post pandemic time interval, if present, would be expected to be gradual with each additional day during T2 and we therefore allowed for a change in slope during T2, compared to T1, with no immediate/abrupt change in level at the first breakpoint. At the second breakpoint, where the proportion of pregnancy exposed to the change in care reached 100% and remained at the level for all subsequent deliveries, we similarly allowed for change in slope (relative to the T2 trend) with no immediate change in level (e.g., levelling off with no further increase/decrease in risk, or a return to the pre-pandemic time trend). We present transformed regression coefficients, interpreted as relative percent change in risk or mean count per 4 -week change in time [(exp(b) – 1) * 4 week *100%] and associated 95% confidence intervals. Formal tests of significance of the differences in slopes between time intervals (T2 vs T1, T3 vs T2, T3 vs T1) were performed with a Wald test.

Minimum detectable between-interval differences. During the study planning phase, minimum detectable between-interval differences in means were calculated (T2 vs T1, T3 vs T2, T3 vs T1), given observed number of deliveries in each of the three-time intervals of interest (N_1 = 75,836; N_2 = 34,799; N_3 = 40,829). Relevant to analyses of binary outcomes, the minimum detectable differences (absolute) in proportions ranged from 0.23% to 1.0% across the expected range in baseline outcome proportions (1.5% to 27%) and contrasts of interest (power = .90, α=.05, two-sided test). The minimum detectable differences in means ranged from 0.021 to 0.024 standard deviation units across the three contrasts of interest.

Multiple imputation. We used multiple imputation[6,7] for variables with missing data such as such as age (0.01%), race-ethnicity (2.3%), NDI (0.15%), pre-pregnancy BMI (3.36%), smoking (0.89%), GDM (4.30%), Cesarean section (5.15%), Apgar score (4.74%), NICU admission (4.74%), and birthweight for gestational age categories (4.76%). An underlying assumption in the application of multiple imputation techniques is that data are missing at random (MAR), meaning that given all observed variables, the probability of a variable being missing is independent from the unobserved data. This assumption cannot be tested with the observed data, and generally must be assessed based on clinical knowledge.[8,9] We note, however, that the frequency of missingness on some variables is low (<5.2%), and therefore any departure from the MAR assumption for these missing variables should result in little or no bias in estimation. In addition, the observed distribution of categories of these variables among individuals with no missing data was consistent with expectation, suggesting missingness being unrelated to unobserved data (MAR). In addition, missingness of these variables was unrelated to the exposure to the pre-pandemic era and the two pandemic eras (Table 1 and Table 2).

ITS Analyses

ITS analyses were conducted after stratifying individuals by NDI quartiles, 4th quartile and first, second and third quartiles combined (eTables 2a and 2b), whether English was the preferred language (eTables 3a and 3b), living in urban or rural area (eTables 4a and 4b), race/ethnicity (eTable 5 for Asian/Pacific Islander, eTable 6 for Black, eTable 7 for Hisapnic, and eTable 8 for White), and whether they had a low-risk pregnancy or not (eTables 9a and 9b). Given the small number of individuals who were Multiracial or American Indian/Native Alaska we did not report the data for them given the imprecision of the estimates.

© 2023 Ferrera A et al. *JAMA Network Open.*

**eTable 1.** ***ICD-10*** **Diagnostic Codes**	
**Conditions**	**ICD-10 diagnostic codes**
COVID-19	U07.1
Pre-gestational chronic hypertension	I10x, I11x, I12x, I13x, I15x, O10x
Gestational hypertension	O13x, O16x
Pre-eclampsia/ Eclampsia	O14x, O15x
Proteinuria	O12.1x, O12.2x
Thrombocytopenia	D69.4x, D69.5x, D69.6x
Pulmonary edema	J81x
Venous Thromboembolism	I26.0x, I26.9x, I80.1x, I80.2x, I80.3x, I80.8x, I80.9x, I82.0x, I82.1x, I82.2x, I82.3x, I82.4x, I82.6x, I82.8x, I82.9x,<br>O08.2x, O22.3x, O22.5x, O87.1x, O88.2x, I81x<br>I82.A1x, I82.B1x, I82.C1x
Stillbirth	O36.4XX0, O36.4XX1, O36.4XX2, O36.4XX3, O36.4XX4, O36.4XX5, O36.4XX9, P95, Z37.1, Z37.4, Z37.7
Cesarean delivery	O82, O90.0, O75.82, P03.4
Cesarean delivery (PX)	10D00Z0, 10D00Z1, 10D00Z2
Transient tachypnea	P22.1
Pre-gestational diabetes	From registry
Gestational diabetes	From registry
Spontaneous delivery	O60.1x
PPROM	O42.x (excludes O42.00, O42.02, O42.10, O42.12, O42.90, O42.92)<br>P01.1
Induced labor (PX)	3E033VJ, 3E0P7GC, 0U7C7ZZ, 0U7C7DZ
Depression	O99.34, F43.23, F43.21, F34.1, F33.9, F33.8, F33.41, F33.40, F33.3, F33.2, F33.1, F33.0, F32.9, F32.8, F32.4, F32.3, F32.2, F32.1, F32.0, F32.5, F33.42
SMM	
Acute myocardial infarction	I21.xx, I22.x
Aneurysm	I71.xx, I79.0
Acute renal failure	N17.x, O90.4
Adult respiratory distress syndrome	J80, J95.1, J95.2, J95.3, J95.82x, J96.0x, J96.2x R09.2
Amniotic fluid embolism	O88.1x
Cardiac arrest/ventricular fibrillation	I46.x, I49.0x
Conversion of cardiac rhythm (PX)	5A2204Z, 5A12012
Disseminated intravascular coagulation	D65, D68.8, D68.9, O72.3
Eclampsia	O15.x
Heart failure/arrest during surgery or procedure	I97.12x, I97.13x, I97.710, I97.711
Puerperal cerebrovascular disorders	I60.xx- I68.xx, O22.51, O22.52, O22.53, I97.81x, I97.82x, O87.3
Pulmonary edema / Acute heart failure	J81.0, I50.1, I50.20, I50.21, I50.23, I50.30, I50.31, I50.33, I50.40, I50.41, I50.43, I50.9
Severe anesthesia complications	O74.0, O74.1, O74.2, O74.3, O89.0x, O89.1, O89.2

© 2023 Ferrera A et al. *JAMA Network Open.*

Sepsis	O85, O86.04, T80.211A, T81.4XXA, T81.44xx, R65.20, A40.x, A41.x, A32.7
Shock	O75.1, R57.x, R65.21, T78.2XXA, T88.2 XXA, T88.6 XXA, T81.10XA, T81.11XA, T81.19XA
Sickle cell disease with crisis	D57.0x, D57.21x, D57.41x, D57.81x
Air and thrombotic embolism	I26.x, O88.0x, O88.2x, O88.3x, O88.8x
Blood products transfusion (PX)	30233H1, 30233L1, 30233K1, 30233M1, 30233N1, 30233P1, 30233R1, 30233T1, 30233H0, 30233L0, 30233K0, 30233M0, 30233N0, 30233P0, 30233R0, 30233T0, 30230H1, 30230L1, 30230K1, 30230M1, 30230N1, 30230P1, 30230R1, 30230T1, 30230H0, 30230L0, 30230K0, 30230M0, 30230N0, 30230P0, 30230R0, 30230T0, 30240H1, 30240L1, 30240K1, 30240M1, 30240N1, 30240P1, 30240R1, 30240T1, 30240H0, 30240L0, 30240K0, 30240M0, 30240N0, 30240P0, 30240R0, 30240T0, 30243H1, 30243L1, 30243K1, 30243M1, 30243N1, 30243P1, 30243R1, 30243T1, 30243H0, 30243L0, 30243K0, 30243M0, 30243N0, 30243P0, 30243R0, 30243T0, 30250H1, 30250L1, 30250K1, 30250M1, 30250N1, 30250P1, 30250R1, 30250T1, 30250H0, 30250L0, 30250K0, 30250M0, 30250N0, 30250P0, 30250R0, 30250T0, 30253H1, 30253L1, 30253K1, 30253M1, 30253N1, 30253P1, 30253R1, 30253T1, 30253H0, 30253L0, 30253K0, 30253M0, 30253N0, 30253P0, 30253R0, 30253T0, 30260H1, 30260L1, 30260K1, 30260M1, 30260N1, 30260P1, 30260R1, 30260T1, 30260H0, 30260L0, 30260K0, 30260M0, 30260N0, 30260P0, 30260R0, 30260T0, 30263H1, 30263L1, 30263K1, 30263M1, 30263N1, 30263P1, 30263R1, 30263T1, 30263H0, 30263L0, 30263K0, 30263M0, 30263N0, 30263P0, 30263R0, 30263T0
Hysterectomy (PX)	0UT90ZZ, 0UT94ZZ, 0UT97ZZ, 0UT98ZZ, 0UT9FZZ
Temporary tracheostomy (PX)	0B110Z, 0B110F, 0B113, 0B114
Ventilation (PX)	5A1935Z, 5A1945Z, 5A1955Z

© 2023 Ferrera A et al. *JAMA Network Open.*

eFigure 1. Associations of Prenatal Depression Screening With a Multimodal Prenatal Healthcare Model During the COVID-19 Pandemic

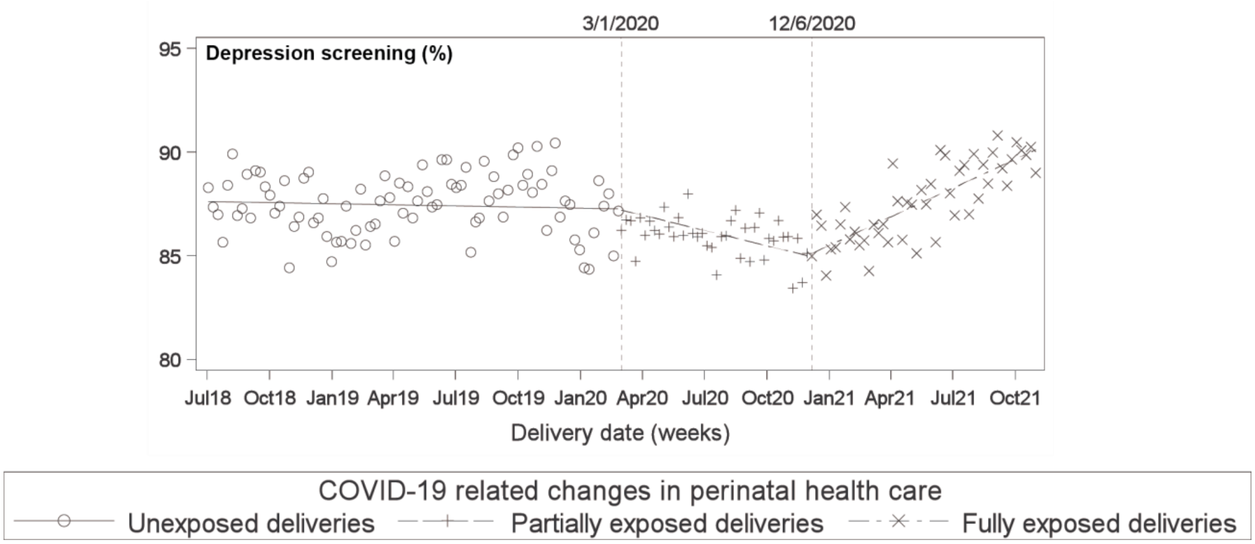

© 2023 Ferrera A et al. *JAMA Network Open.*

eFigure 2. Secondary Health Outcomes in Association With Exposure to COVID-19 Pandemic–Related Implementation of a Multimodal Prenatal Health Care Delivery Among 151 464 Individuals

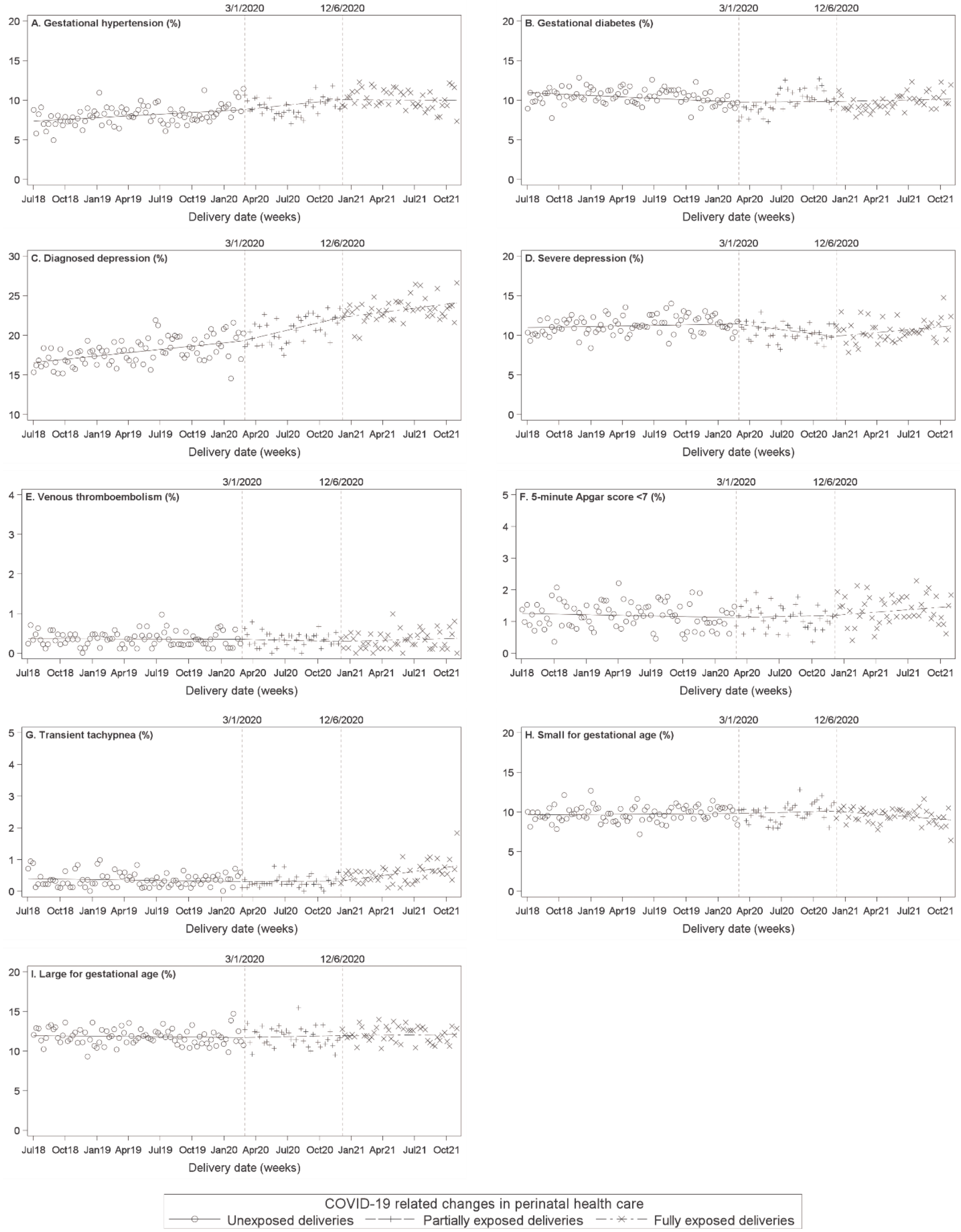

© 2023 Ferrera A et al. *JAMA Network Open.*

eTable 2. Interrupted time Series Analysis for the Trends in Prenatal Visits, Care Process and Health Outcomes in Association With Exposure to COVID-19 Pandemic–Related Implementation of a Multimodal Prenatal Health Care Delivery Among 36 167 Individuals Who Were in the **Highest Quartile of the Neighborhood Deprivation Index** and Delivered a Singleton Live Birth or Stillbirth, Kaiser Permanente Northern California, 2018-2021

	Percent Change (95% CI) per 4 weeks						P value [b]		
	**Unexposed** 7/1/18-2/29/20 T1		**Partially Exposed** 3/1/20-12/5/20 T2		**Fully Exposed** 12/6/20-10/31/21 T3				
	Unadjusted	Adjusted [a]	Unadjusted	Adjusted [a]	Unadjusted	Adjusted [a]	T2 vs. T1	T3 vs. T1	T3 vs. T2
**Prenatal care visits**									
Total prenatal in-office and virtual clinic visits per individual	-0.30 (-0.37 to -0.24)	-0.30 (-0.36 to -0.23)	0.45 (0.26 to 0.64)	0.44 (0.26 to 0.63)	-0.69 (-0.95 to -0.44)	-0.75 (-1.00 to -0.49)	<.001	<.001	<.001
In office prenatal clinic visits per individual	-0.30 (-0.37 to -0.23)	-0.29 (-0.37 to -0.22)	-1.31 (-1.51 to -1.12)	-1.33 (-1.53 to -1.13)	2.09 (1.80 to 2.38)	2.04 (1.75 to 2.33)	<.001	<.001	<.001
Telemedicine prenatal clinic visits (video or telephone) per individual	-0.00 (-0.19 to 0.18)	-0.02 (-0.21 to 0.16)	8.38 (7.88 to 8.90)	8.43 (7.92 to 8.94)	-11.7 (-12.2 to -11.2)	-11.8 (-12.3 to -11.3)	<.001	<.001	<.001
Video prenatal visits per individual	16.18 (13.48 to 19.15)	16.23 (13.52 to 19.21)	21.35 (17.23 to 25.48)	21.35 (17.22 to 25.48)	-33.6 (-34.7 to -32.4)	-33.6 (-34.8 to -32.5)	<.01	<.001	<.001
Telephone prenatal visits per individual	0.13 (-0.06 to 0.32)	0.11 (-0.08 to 0.30)	4.28 (3.76 to 4.80)	4.32 (3.81 to 4.84)	-8.30 (-8.90 to -7.70)	-8.44 (-9.04 to -7.84)	<.001	<.001	<.001
**Process care process**									
Blood pressure measurements per individual	-0.07 (-0.12 to -0.02)	-0.08 (-0.12 to -0.03)	-0.56 (-0.69 to -0.43)	-0.51 (-0.64 to -0.38)	1.01 (0.83 to 1.20)	0.85 (0.67 to 1.04)	<.001	<.001	<.001
Gestational diabetes screening[c]	0.01 (-0.06 to 0.08)	-0.00 (-0.07 to 0.07)	0.10 (-0.10 to 0.29)	0.13 (-0.06 to 0.32)	-0.01 (-0.28 to 0.26)	-0.07 (-0.33 to 0.18)	0.07	0.45	0.08
Depression screening [d]	0.04 (-0.04 to 0.12)	0.02 (-0.06 to 0.10)	-0.26 (-0.48 to -0.04)	-0.24 (-0.45 to -0.02)	0.63 (0.33 to 0.94)	0.62 (0.33 to 0.91)	<.01	<.001	<.001
**Primary prenatal maternal outcomes**									
Pre-eclampsia/Eclampsia	1.41 (0.65 to 2.19)	1.18 (0.43 to 1.94)	-1.28 (-3.24 to 0.72)	-0.77 (-2.70 to 1.20)	-0.47 (-3.11 to 2.26)	-0.84 (-3.42 to 1.81)	0.01	0.04	0.95
Severe maternal morbidity [e]	1.24 (0.26 to 2.25)	1.24 (0.27 to 2.22)	-2.38 (-4.93 to 0.24)	-2.51 (-5.00 to 0.04)	2.96 (-0.64 to 6.73)	2.81 (-0.73 to 6.48)	<.001	0.24	<.001
Cesarean section	0.40 (0.04 to 0.76)	0.27 (-0.07 to 0.62)	-0.24 (-1.21 to 0.73)	-0.17 (-1.09 to 0.76)	-0.24 (-1.55 to 1.09)	-0.39 (-1.64 to 0.87)	0.21	0.16	0.69
Preterm birth	0.55 (-0.09 to 1.20)	0.55 (-0.08 to 1.18)	0.14 (-1.57 to 1.88)	0.14 (-1.53 to 1.84)	-2.03 (-4.31 to 0.30)	-2.08 (-4.29 to 0.18)	0.53	<.01	0.03
Spontaneous preterm birth	0.16 (-0.73 to 1.07)	0.24 (-0.64 to 1.12)	-0.19 (-2.63 to 2.32)	-0.20 (-2.57 to 2.24)	-0.86 (-4.20 to 2.62)	-1.02 (-4.26 to 2.34)	0.64	0.31	0.58
Medically indicated preterm birth	0.99 (0.03 to 1.97)	0.91 (-0.03 to 1.87)	0.39 (-2.11 to 2.97)	0.39 (-2.06 to 2.91)	-3.17 (-6.41 to 0.19)	-3.10 (-6.26 to 0.16)	0.59	<.01	0.02
**Primary newborn outcome**									
Neonatal intensive care unit admission	0.27 (-0.33 to 0.88)	0.18 (-0.41 to 0.78)	-1.61 (-3.26 to 0.07)	-1.43 (-3.04 to 0.20)	1.95 (-0.41 to 4.38)	1.58 (-0.71 to 3.92)	<.01	0.1	<.01
**Secondary prenatal maternal outcomes**									

© 2023 Ferrera A et al. *JAMA Network Open.*

Gestational hypertension	0.61 (-0.07 to 1.31)	0.57 (-0.11 to 1.25)	0.28 (-1.54 to 2.15)	0.52 (-1.26 to 2.33)	-1.50 (-3.90 to 0.98)	-1.66 (-3.98 to 0.71)	0.94	0.01	0.04
Gestational diabetes	-0.79 (-1.37 to -0.21)	-0.94 (-1.48 to -0.39)	1.67 (0.02 to 3.35)	1.65 (0.09 to 3.24)	-1.48 (-3.67 to 0.78)	-1.65 (-3.73 to 0.47)	<.001	0.36	<.001
Diagnosed depression	0.51 (0.07 to 0.94)	0.49 (0.07 to 0.91)	1.37 (0.23 to 2.53)	1.43 (0.33 to 2.55)	-1.34 (-2.77 to 0.12)	-1.53 (-2.90 to -0.15)	0.03	<.001	<.001
Moderated to severe depression [f]	-0.09 (-0.62 to 0.45)	-0.10 (-0.63 to 0.42)	-1.19 (-2.68 to 0.33)	-1.09 (-2.55 to 0.38)	2.02 (-0.14 to 4.24)	1.93 (-0.17 to 4.08)	0.08	<.01	<.01
Venous thromboembolism	1.18 (-2.09 to 4.63)	0.75 (-2.45 to 4.06)	-2.32 (-10.7 to 6.90)	-1.57 (-9.88 to 7.51)	0.11 (-11.6 to 13.74)	-0.78 (-12.3 to 12.25)	0.49	0.74	0.88
**Secondary newborn outcomes**									
Transient Tachypnea	-2.30 (-5.75 to 1.30)	-2.51 (-5.86 to 0.96)	7.70 (-2.22 to 18.92)	7.81 (-2.01 to 18.63)	1.75 (-9.36 to 14.59)	1.57 (-9.48 to 13.97)	<.01	0.35	0.27
5-minute APGAR score <7	0.15 (-1.66 to 2.01)	-0.05 (-1.82 to 1.75)	1.00 (-3.90 to 6.17)	1.49 (-3.30 to 6.52)	-2.58 (-8.92 to 4.29)	-3.01 (-9.14 to 3.54)	0.41	0.22	0.12
Small for gestational age	-0.18 (-0.81 to 0.47)	-0.07 (-0.69 to 0.56)	0.85 (-0.92 to 2.65)	0.67 (-1.05 to 2.42)	-1.63 (-3.99 to 0.79)	-1.39 (-3.70 to 0.97)	0.27	0.13	0.05
Large for gestational age	0.20 (-0.35 to 0.76)	-0.04 (-0.58 to 0.49)	0.33 (-1.17 to 1.86)	0.63 (-0.82 to 2.11)	-1.71 (-3.71 to 0.35)	-1.75 (-3.68 to 0.23)	0.23	0.02	<.01

Individuals with delivery in T2 did not experience the multimodal prenatal healthcare delivery implemented during COVID-19 pandemic for the entire duration of the pregnancy, individuals with delivery in T3 experienced the multimodal prenatal healthcare delivery for the entire duration of the pregnancy.

Interrupted time-series methodology for the trends in prenatal outcomes before and during the implementation of COVID-19 pandemic-related changes in prenatal healthcare used piecewise linear segmented regression, allowing a change in slope associated with time at two inflection points: 3/1/20 (initiation of changes in prenatal healthcare) and 12/6/20 (date which pregnancies were fully exposed to the prenatal healthcare changes). Log binomial regression was used to examine each binary outcome in relation to date of delivery (week), providing point and interval estimates of relative percent change in outcome probability associated with change in time, scaled to a 4-week change. Poisson regression was used for point and interval estimation of relative percent change in means for outcomes characterized as counts in relation to a 4-week change in delivery date.

[a] Adjusted for age, pre-pregnancy body mass index, and race/ethnicity.

[b] Two side Wald test adjusted for age, pre-pregnancy body mass index, and race/ethnicity. [c] Screening by 50-g, 1-h oral glucose challenge test; [d] Screening by Patient Health Questionnaire.

[e] Severe Maternal morbidity includes at least one of the following morbidities occurring at any time during pregnancy: acute myocardial infarction, aneurysm, acute renal failure, adult respiratory distress syndrome, amniotic fluid embolism, cardiac arrest/ventricular fibrillation, conversion of cardiac rhythm, disseminated intravascular coagulation, eclampsia, heart failure/arrest during surgery or procedure, puerperal cerebrovascular disorders, pulmonary edema/acute heart failure, severe anesthesia complications, sepsis, shock, sickle cell disease with crisis, air and thrombotic embolism, blood products transfusion, hysterectomy, temporary tracheostomy, or ventilation.[10-12]

[f] Patient Health Questionnaire screening scores (moderate to severe≥10).[13]

© 2023 Ferrera A et al. *JAMA Network Open.*

eTable 3. Interrupted Time Series Analysis for the Trends in Prenatal Visits, Care Process, and Health Outcomes in Association With Exposure to COVID-19 Pandemic–Related Implementation of a Multimodal Prenatal Health Care Delivery Among 113 602 Individuals Who Were in **the First, Second or Third Quartile of the Neighborhood Deprivation Index** and Delivered a Singleton Live Birth or Stillbirth, Kaiser Permanente Northern California, 2018-2021

	Percent Change (95% CI) per 4 weeks						P value [b]		
	**Unexposed** 7/1/18-2/29/20 T1		**Partially Exposed** 3/1/20-12/5/20 T2		**Fully Exposed** 12/6/20-10/31/21 T3				
	Unadjusted	Adjusted [a]	Unadjusted	Adjusted [a]	Unadjusted	Adjusted [a]	T2 vs. T1	T3 vs. T1	T3 vs. T2
**Prenatal care visits**									
Total prenatal in-office and virtual clinic visits per individual	-0.26 (-0.29 to -0.22)	-0.27 (-0.31 to -0.23)	0.45 (0.35 to 0.55)	0.48 (0.38 to 0.59)	-0.38 (-0.53 to -0.24)	-0.45 (-0.59 to -0.31)	<.001	<.001	<.001
In office prenatal clinic visits per individual	-0.24 (-0.28 to -0.20)	-0.25 (-0.29 to -0.21)	-1.66 (-1.77 to -1.55)	-1.63 (-1.74 to -1.52)	2.96 (2.80 to 3.13)	2.90 (2.74 to 3.07)	<.001	<.001	<.001
Telemedicine prenatal clinic visits (video or telephone) per individual	-0.02 (-0.12 to 0.09)	-0.06 (-0.16 to 0.05)	10.08 (9.79 to 10.38)	10.16 (9.87 to 10.46)	-13.4 (-13.7 to -13.1)	-13.5 (-13.8 to -13.2)	<.001	<.001	<.001
Video prenatal visits per individual	16.28 (15.10 to 17.51)	16.26 (15.09 to 17.49)	18.49 (16.76 to 20.23)	18.59 (16.86 to 20.33)	-31.8 (-32.3 to -31.2)	-31.9 (-32.4 to -31.3)	<.01	<.001	<.001
Telephone prenatal visits per individual	0.09 (-0.02 to 0.20)	0.05 (-0.06 to 0.16)	4.47 (4.17 to 4.77)	4.54 (4.23 to 4.84)	-9.05 (-9.41 to -8.70)	-9.18 (-9.54 to -8.83)	<.001	<.001	<.001
**Process care process**									
Blood pressure measurements per individual	-0.07 (-0.10 to -0.05)	-0.09 (-0.11 to -0.06)	-0.77 (-0.84 to -0.69)	-0.73 (-0.81 to -0.66)	1.52 (1.41 to 1.63)	1.41 (1.30 to 1.51)	<.001	<.001	<.001
Gestational diabetes screening[c]	0.01 (-0.02 to 0.05)	0.02 (-0.01 to 0.05)	-0.05 (-0.14 to 0.03)	-0.06 (-0.15 to 0.02)	0.11 (-0.01 to 0.24)	0.09 (-0.03 to 0.21)	0.01	0.14	<.01
Depression screening [d]	-0.03 (-0.08 to 0.01)	-0.03 (-0.07 to 0.01)	-0.26 (-0.38 to -0.13)	-0.27 (-0.39 to -0.15)	0.82 (0.65 to 1.00)	0.84 (0.67 to 1.01)	<.001	<.001	<.001
**Primary prenatal maternal outcomes**									
Pre-eclampsia/Eclampsia	0.61 (0.18 to 1.04)	0.64 (0.20 to 1.07)	0.07 (-1.08 to 1.23)	-0.03 (-1.18 to 1.14)	-0.80 (-2.34 to 0.76)	-0.76 (-2.31 to 0.81)	0.14	0.02	0.3
Severe maternal morbidity [e]	-0.25 (-0.84 to 0.35)	-0.35 (-0.95 to 0.26)	1.31 (-0.34 to 2.99)	1.60 (-0.07 to 3.30)	0.48 (-1.70 to 2.72)	0.26 (-1.94 to 2.50)	<.01	0.47	0.18
Cesarean section	0.07 (-0.12 to 0.27)	-0.01 (-0.20 to 0.18)	-0.02 (-0.56 to 0.52)	0.03 (-0.50 to 0.56)	0.17 (-0.57 to 0.91)	0.07 (-0.66 to 0.81)	0.85	0.76	0.89
Preterm birth	0.19 (-0.20 to 0.59)	0.08 (-0.31 to 0.48)	-0.70 (-1.78 to 0.40)	-0.58 (-1.68 to 0.52)	0.81 (-0.72 to 2.37)	0.71 (-0.82 to 2.27)	0.11	0.27	0.06
Spontaneous preterm birth	-0.28 (-0.81 to 0.27)	-0.35 (-0.90 to 0.19)	-0.50 (-2.01 to 1.04)	-0.41 (-1.93 to 1.15)	1.03 (-1.12 to 3.25)	1.01 (-1.17 to 3.24)	0.93	0.09	0.14
Medically indicated preterm birth	0.77 (0.17 to 1.39)	0.62 (0.01 to 1.23)	-1.01 (-2.62 to 0.63)	-0.86 (-2.49 to 0.79)	0.59 (-1.65 to 2.88)	0.41 (-1.84 to 2.71)	0.02	0.81	0.21
**Primary newborn outcome**									
Neonatal intensive care unit admission	-0.33 (-0.69 to 0.03)	-0.39 (-0.75 to -0.03)	-0.68 (-1.70 to 0.35)	-0.67 (-1.69 to 0.37)	1.63 (0.16 to 3.12)	1.62 (0.14 to 3.13)	0.49	<.001	<.001
**Secondary prenatal maternal outcomes**									

© 2023 Ferrera A et al. *JAMA Network Open.*

Gestational hypertension	0.91 (0.52 to 1.29)	0.85 (0.47 to 1.23)	0.49 (-0.52 to 1.50)	0.49 (-0.52 to 1.51)	-1.08 (-2.38 to 0.23)	-1.17 (-2.47 to 0.15)	0.36	<.001	<.01
Gestational diabetes	-0.45 (-0.80 to -0.11)	-0.22 (-0.62 to 0.19)	0.33 (-0.65 to 1.31)	0.28 (-0.12 to 0.69)	0.55 (-0.82 to 1.93)	-0.08 (-0.50 to 0.35)	0.01	0.49	0.09
Diagnosed depression	0.77 (0.53 to 1.01)	0.77 (0.53 to 1.01)	0.54 (-0.10 to 1.18)	0.48 (-0.14 to 1.12)	-0.67 (-1.48 to 0.16)	-0.71 (-1.52 to 0.10)	0.25	<.001	<.01
Moderated to severe depression [f]	0.32 (-0.04 to 0.68)	0.28 (-0.09 to 0.64)	-1.93 (-2.92 to -0.93)	-1.89 (-2.88 to -0.89)	2.81 (1.35 to 4.29)	2.79 (1.32 to 4.27)	<.001	<.001	<.001
Venous thromboembolism	-0.56 (-2.44 to 1.37)	-0.59 (-2.49 to 1.36)	-1.92 (-7.21 to 3.69)	-1.85 (-7.19 to 3.80)	6.81 (-1.12 to 15.57)	6.49 (-1.55 to 15.18)	0.55	0.02	0.02
**Secondary newborn outcomes**									
Transient Tachypnea	-0.95 (-2.81 to 0.95)	-0.93 (-2.81 to 0.98)	0.93 (-4.27 to 6.45)	1.08 (-4.17 to 6.62)	7.20 (0.10 to 14.96)	6.75 (-0.41 to 14.42)	0.33	<.01	0.08
5-minute APGAR score <7	-0.77 (-1.83 to 0.31)	-0.76 (-1.83 to 0.32)	1.28 (-1.72 to 4.39)	1.08 (-1.95 to 4.21)	2.23 (-1.82 to 6.48)	2.52 (-1.60 to 6.81)	0.11	0.03	0.44
Small for gestational age	0.16 (-0.20 to 0.51)	0.13 (-0.22 to 0.49)	-0.01 (-0.99 to 0.97)	0.21 (-0.76 to 1.20)	-0.83 (-2.17 to 0.53)	-0.99 (-2.32 to 0.36)	0.83	0.03	0.04
Large for gestational age	-0.20 (-0.52 to 0.12)	-0.25 (-0.57 to 0.07)	0.30 (-0.59 to 1.21)	0.32 (-0.57 to 1.22)	0.37 (-0.86 to 1.62)	0.16 (-1.06 to 1.40)	0.09	0.36	0.78

Individuals with delivery in T2 did not experience the multimodal prenatal healthcare delivery implemented during COVID-19 pandemic for the entire duration of the pregnancy, individuals with delivery in T3 experienced the multimodal prenatal healthcare delivery for the entire duration of the pregnancy.

Interrupted time-series methodology for the trends in prenatal outcomes before and during the implementation of COVID-19 pandemic-related changes in prenatal healthcare used piecewise linear segmented regression, allowing a change in slope associated with time at two inflection points: 3/1/20 (initiation of changes in prenatal healthcare) and 12/6/20 (date which pregnancies were fully exposed to the prenatal healthcare changes). Log binomial regression was used to examine each binary outcome in relation to date of delivery (week), providing point and interval estimates of relative percent change in outcome probability associated with change in time, scaled to a 4-week change. Poisson regression was used for point and interval estimation of relative percent change in means for outcomes characterized as counts in relation to a 4-week change in delivery date.

[a] Adjusted for age, pre-pregnancy body mass index, and race/ethnicity.

[b] Two side Wald test adjusted for age, pre-pregnancy body mass index, and race/ethnicity. [c] Screening by 50-g, 1-h oral glucose challenge test; [d] Screening by Patient Health Questionnaire.

[e] Severe Maternal morbidity includes at least one of the following morbidities occurring at any time during pregnancy: acute myocardial infarction, aneurysm, acute renal failure, adult respiratory distress syndrome, amniotic fluid embolism, cardiac arrest/ventricular fibrillation, conversion of cardiac rhythm, disseminated intravascular coagulation, eclampsia, heart failure/arrest during surgery or procedure, puerperal cerebrovascular disorders, pulmonary edema/acute heart failure, severe anesthesia complications, sepsis, shock, sickle cell disease with crisis, air and thrombotic embolism, blood products transfusion, hysterectomy, temporary tracheostomy, or ventilation.[10-12]

[f] Patient Health Questionnaire screening scores (moderate to severe≥10).[13]

© 2023 Ferrera A et al. *JAMA Network Open.*

eTable 4. Interrupted Time Series Analysis for the Trends in Prenatal Visits, Care Process, and Health Outcomes in Association With Exposure to COVID-19 Pandemic–Related Implementation of a Multimodal Prenatal Health Care Delivery Among 141 683 Individuals **Who Preferred English Language** and Delivered a Singleton Live Birth or Stillbirth, Kaiser Permanente, Northern California, 2018-2021

	Percent Change (95% CI) per 4 weeks						P value [b]		
	**Unexposed T1** 7/1/18-2/29/20 deliveries		**Partially Exposed T2** 3/1/20-12/5/20 deliveries		**Fully Exposed T3** 12/6/20-10/31/21 deliveries				
	Unadjusted	Adjusted [a]	Unadjusted	Adjusted [a]	Unadjusted	Adjusted [a]	T2 vs. T1	T3 vs. T1	T3 vs. T2
**Prenatal care visits**									
Total prenatal in-office and telemedicine visits per individual	-0.26 (-0.29 to -0.23)	-0.27 (-0.31 to -0.24)	0.41 (0.31 to 0.50)	0.45 (0.35 to 0.54)	-0.40 (-0.53 to -0.27)	-0.48 (-0.61 to -0.35)	<.001	<.001	<.001
In-office prenatal visits per individual	-0.24 (-0.28 to -0.21)	-0.26 (-0.29 to -0.22)	-1.61 (-1.71 to -1.51)	-1.58 (-1.68 to -1.48)	2.79 (2.65 to 2.94)	2.72 (2.57 to 2.87)	<.001	<.001	<.001
Telemedicine prenatal visits (video or telephone) per individual	-0.01 (-0.11 to 0.08)	-0.05 (-0.15 to 0.05)	9.66 (9.39 to 9.92)	9.75 (9.48 to 10.01)	-12.9 (-13.2 to -12.7)	-13.1 (-13.3 to -12.8)	<.001	<.001	<.001
Video prenatal visits per individual	16.33 (15.22 to 17.48)	16.22 (15.11 to 17.37)	18.65 (17.02 to 20.27)	18.90 (17.27 to 20.53)	-31.9 (-32.4 to -31.4)	-32.1 (-32.6 to -31.6)	<.001	<.001	<.001
Telephone prenatal visits per individual	0.10 (0.01 to 0.20)	0.07 (-0.02 to 0.17)	4.29 (4.02 to 4.56)	4.34 (4.07 to 4.61)	-8.65 (-8.96 to -8.33)	-8.78 (-9.09 to -8.46)	<.001	<.001	<.001
**Prenatal care process**									
Blood pressure measurements per individual	-0.06 (-0.09 to -0.04)	-0.08 (-0.10 to -0.06)	-0.73 (-0.80 to -0.67)	-0.68 (-0.75 to -0.62)	1.40 (1.30 to 1.49)	1.27 (1.17 to 1.36)	<.001	<.001	<.001
Gestational diabetes screening [c]	0.01 (-0.02 to 0.04)	0.01 (-0.01 to 0.04)	-0.02 (-0.10 to 0.07)	-0.03 (-0.11 to 0.05)	0.07 (-0.04 to 0.19)	0.06 (-0.05 to 0.17)	0.15	0.28	0.07
Depression screening [d]	-0.01 (-0.05 to 0.03)	-0.01 (-0.05 to 0.03)	-0.26 (-0.36 to -0.15)	-0.27 (-0.37 to -0.16)	0.76 (0.61 to 0.91)	0.77 (0.62 to 0.92)	<.001	<.001	<.001
**Primary prenatal maternal outcomes**									
Pre-eclampsia/Eclampsia	0.79 (0.40 to 1.18)	0.75 (0.37 to 1.14)	-0.24 (-1.26 to 0.80)	-0.19 (-1.21 to 0.85)	-0.71 (-2.08 to 0.69)	-0.76 (-2.13 to 0.63)	0.02	<.01	0.36
Severe maternal morbidity [e]	0.13 (-0.40 to 0.66)	0.09 (-0.44 to 0.62)	0.25 (-1.19 to 1.71)	0.31 (-1.13 to 1.77)	1.26 (-0.68 to 3.25)	1.17 (-0.77 to 3.15)	0.7	0.14	0.33
Cesarean section	0.13 (-0.04 to 0.31)	0.05 (-0.12 to 0.22)	-0.02 (-0.51 to 0.47)	-0.01 (-0.49 to 0.46)	0.05 (-0.62 to 0.72)	-0.02 (-0.67 to 0.64)	0.73	0.78	0.99
Preterm birth	0.30 (-0.05 to 0.65)	0.24 (-0.11 to 0.58)	-0.52 (-1.47 to 0.44)	-0.43 (-1.38 to 0.52)	0.15 (-1.17 to 1.49)	-0.01 (-1.31 to 1.32)	0.07	0.62	0.46
Spontaneous preterm birth	-0.22 (-0.70 to 0.26)	-0.25 (-0.73 to 0.23)	-0.32 (-1.65 to 1.03)	-0.27 (-1.61 to 1.08)	0.56 (-1.32 to 2.48)	0.48 (-1.40 to 2.40)	0.96	0.3	0.37
Medically indicated preterm birth	0.93 (0.40 to 1.47)	0.82 (0.29 to 1.35)	-0.84 (-2.24 to 0.59)	-0.71 (-2.11 to 0.71)	-0.25 (-2.16 to 1.71)	-0.47 (-2.37 to 1.47)	<.01	0.07	0.78
**Primary newborn outcome**									

© 2023 Ferrera A et al. *JAMA Network Open.*

Neonatal intensive care unit admission	-0.13 (-0.45 to 0.19)	-0.18 (-0.50 to 0.13)	-1.06 (-1.96 to -0.16)	-1.02 (-1.91 to -0.12)	1.76 (0.47 to 3.07)	1.66 (0.37 to 2.96)	0.01	<.001	<.001
**Secondary prenatal maternal outcomes**									
Gestational hypertension	0.86 (0.52 to 1.20)	0.78 (0.44 to 1.13)	0.28 (-0.62 to 1.19)	0.38 (-0.52 to 1.29)	-0.91 (-2.08 to 0.28)	-1.05 (-2.22 to 0.13)	0.25	<.001	<.01
Gestational diabetes	-0.60 (-0.91 to -0.29)	-0.81 (-1.11 to -0.51)	0.85 (-0.04 to 1.75)	1.10 (0.24 to 1.97)	-0.13 (-1.35 to 1.11)	-0.60 (-1.80 to 0.61)	<.001	0.64	<.01
Diagnosed depression	0.75 (0.53 to 0.96)	0.74 (0.53 to 0.95)	0.67 (0.11 to 1.23)	0.64 (0.09 to 1.19)	-0.86 (-1.58 to -0.14)	-0.89 (-1.59 to -0.18)	0.65	<.001	<.001
Moderate to severe depression [f]	0.24 (-0.07 to 0.55)	0.21 (-0.10 to 0.52)	-1.79 (-2.65 to -0.93)	-1.74 (-2.59 to -0.89)	2.60 (1.35 to 3.88)	2.59 (1.35 to 3.85)	<.001	<.001	<.001
Venous thromboembolism	-0.12 (-1.81 to 1.61)	-0.23 (-1.92 to 1.49)	-1.60 (-6.26 to 3.30)	-1.50 (-6.17 to 3.40)	3.66 (-3.12 to 11.03)	3.47 (-3.35 to 10.77)	0.49	0.15	0.1
**Secondary newborn outcome**									
Newborn Transient Tachypnea	-1.02 (-2.73 to 0.74)	-1.03 (-2.75 to 0.71)	2.34 (-2.46 to 7.42)	2.31 (-2.51 to 7.37)	4.98 (-1.25 to 11.72)	4.94 (-1.34 to 11.62)	0.07	0.01	0.37
Newborn 5-minute APGAR score <7	-0.61 (-1.55 to 0.35)	-0.67 (-1.61 to 0.29)	1.68 (-0.97 to 4.40)	1.74 (-0.91 to 4.46)	0.29 (-3.19 to 3.93)	0.19 (-3.29 to 3.81)	0.02	0.52	0.34
Birthweight small for gestational age	0.06 (-0.26 to 0.38)	0.06 (-0.26 to 0.38)	0.17 (-0.71 to 1.06)	0.33 (-0.54 to 1.22)	-0.92 (-2.12 to 0.31)	-1.03 (-2.23 to 0.18)	0.42	0.02	0.01
Birthweight large for gestational age	-0.13 (-0.42 to 0.15)	-0.22 (-0.50 to 0.07)	0.50 (-0.30 to 1.30)	0.52 (-0.26 to 1.31)	-0.47 (-1.54 to 0.62)	-0.56 (-1.62 to 0.51)	0.01	0.39	0.02

Individuals with delivery in T2 did not experience the multimodal prenatal healthcare delivery implemented during COVID-19 pandemic for the entire duration of the pregnancy, individuals with delivery in T3 experienced the multimodal prenatal healthcare delivery for the entire duration of the pregnancy.

Interrupted time-series methodology for the trends in prenatal outcomes before and during the implementation of COVID-19 pandemic-related changes in prenatal healthcare used piecewise linear segmented regression, allowing a change in slope associated with time at two inflection points: 3/1/20 (initiation of changes in prenatal healthcare) and 12/6/20 (date which pregnancies were fully exposed to the prenatal healthcare changes). Log binomial regression was used to examine each binary outcome in relation to date of delivery (week), providing point and interval estimates of relative percent change in outcome probability associated with change in time, scaled to a 4-week change. Poisson regression was used for point and interval estimation of relative percent change in means for outcomes characterized as counts in relation to a 4-week change in delivery date.

[a] Adjusted for age, pre-pregnancy body mass index, neighborhood deprivation index and race/ethnicity.

[b] Two side Wald test adjusted for age, pre-pregnancy body mass index, neighborhood deprivation index and race/ethnicity. [c] Screening by 50-g, 1-h oral glucose challenge test; [d] Screening by Patient Health Questionnaire.

[e] Severe Maternal morbidity includes at least one of the following morbidities occurring at any time during pregnancy: acute myocardial infarction, aneurysm, acute renal failure, adult respiratory distress syndrome, amniotic fluid embolism, cardiac arrest/ventricular fibrillation, conversion of cardiac rhythm, disseminated intravascular coagulation, eclampsia, heart failure/arrest during surgery or procedure, puerperal cerebrovascular disorders, pulmonary edema/acute heart failure, severe anesthesia complications, sepsis, shock, sickle cell disease with crisis, air and thrombotic embolism, blood products transfusion, hysterectomy, temporary tracheostomy, or ventilation.[10-12]

[f] Patient Health Questionnaire screening scores (moderate to severe≥10).[13]

© 2023 Ferrera A et al. *JAMA Network Open.*

eTable 5. Interrupted Time Series Analysis for the Trends in Prenatal Visits, Care Process, and Health Outcomes in Association With Exposure To COVID-19 Pandemic–Related Implementation of a Multimodal Prenatal Health Care Delivery Among 9781 Individuals Who **Did Not Prefer English Language** and Delivered a Singleton Live Birth Or Stillbirth, Kaiser Permanente Northern California, 2018-2021

	Percent Change (95% CI) per 4 weeks						P value [b]		
	**Unexposed T1** 7/1/18-2/29/20 deliveries		**Partially Exposed T2** 3/1/20-12/5/20 deliveries		**Fully Exposed T3** 12/6/20-10/31/21 deliveries				
	Unadjusted	Adjusted [a]	Unadjusted	Adjusted [a]	Unadjusted	Adjusted [a]	T2 vs. T1	T3 vs. T1	T3 vs. T2
**Prenatal care visits**									
Total prenatal in-office and telemedicine visits per individual	-0.40 (-0.53 to -0.27)	-0.38 (-0.50 to -0.25)	1.02 (0.66 to 1.39)	0.96 (0.59 to 1.33)	-1.36 (-1.86 to -0.85)	-1.26 (-1.76 to -0.75)	<.001	<.001	<.001
In-office prenatal visits per individual	-0.39 (-0.53 to -0.26)	-0.37 (-0.51 to -0.23)	-1.06 (-1.45 to -0.67)	-1.12 (-1.51 to -0.72)	2.04 (1.45 to 2.63)	2.16 (1.57 to 2.75)	<.001	<.001	<.001
Telemedicine prenatal visits (video or telephone) per individual	-0.05 (-0.41 to 0.30)	-0.01 (-0.37 to 0.35)	9.90 (8.90 to 10.90)	9.74 (8.75 to 10.74)	-13.9 (-14.9 to -12.9)	-13.8 (-14.8 to -12.9)	<.001	<.001	<.001
Video prenatal visits per individual	15.85 (10.72 to 22.11)	15.69 (10.54 to 21.98)	23.65 (15.25 to 32.05)	24.12 (15.64 to 32.61)	-34.2 (-36.5 to -31.9)	-34.4 (-36.7 to -32.0)	0.02	<.001	<.001
Telephone prenatal visits per individual	0.08 (-0.28 to 0.45)	0.15 (-0.21 to 0.52)	6.13 (5.13 to 7.14)	5.92 (4.92 to 6.94)	-11.6 (-12.7 to -10.5)	-11.5 (-12.6 to -10.4)	<.001	<.001	<.001
**Prenatal care process**									
Blood pressure measurements per individual	-0.18 (-0.28 to -0.09)	-0.16 (-0.25 to -0.06)	-0.47 (-0.74 to -0.20)	-0.54 (-0.81 to -0.27)	1.19 (0.80 to 1.57)	1.23 (0.84 to 1.61)	<.001	<.001	<.001
Gestational diabetes screening [c]	0.05 (-0.08 to 0.18)	0.01 (-0.10 to 0.13)	-0.01 (-0.36 to 0.34)	0.02 (-0.30 to 0.34)	0.04 (-0.46 to 0.55)	0.03 (-0.43 to 0.50)	0.98	0.92	0.95
Depression screening [d]	-0.08 (-0.25 to 0.09)	-0.09 (-0.25 to 0.06)	-0.28 (-0.76 to 0.21)	-0.27 (-0.73 to 0.20)	0.87 (0.17 to 1.59)	0.81 (0.14 to 1.49)	0.33	<.001	<.001
**Primary prenatal maternal outcomes**									
Pre-eclampsia/Eclampsia	1.00 (-0.48 to 2.53)	0.87 (-0.62 to 2.38)	-0.36 (-4.25 to 3.70)	-0.17 (-4.06 to 3.88)	-1.43 (-6.67 to 4.15)	-1.48 (-6.72 to 4.04)	0.5	0.25	0.59
Severe maternal morbidity [e]	0.49 (-1.52 to 2.57)	0.45 (-1.57 to 2.51)	1.44 (-3.89 to 7.12)	1.44 (-3.90 to 7.09)	-1.26 (-8.08 to 6.16)	-1.21 (-8.06 to 6.14)	0.64	0.53	0.41
Cesarean section	0.33 (-0.32 to 1.00)	0.22 (-0.43 to 0.87)	-0.63 (-2.42 to 1.19)	-0.34 (-2.11 to 1.45)	0.26 (-2.24 to 2.84)	-0.16 (-2.61 to 2.34)	0.41	0.68	0.87
Preterm birth	0.19 (-1.12 to 1.52)	0.11 (-1.20 to 1.44)	0.46 (-3.12 to 4.18)	0.55 (-3.02 to 4.25)	-2.23 (-7.09 to 2.92)	-2.32 (-7.16 to 2.77)	0.75	0.19	0.2
Spontaneous preterm birth	0.78 (-1.04 to 2.66)	0.81 (-1.02 to 2.68)	-1.18 (-6.02 to 3.92)	-1.37 (-6.20 to 3.71)	-0.85 (-7.66 to 6.53)	-0.70 (-7.54 to 6.64)	0.26	0.57	0.83
Medically indicated preterm birth	-0.50 (-2.45 to 1.50)	-0.69 (-2.63 to 1.29)	2.44 (-3.03 to 8.25)	2.88 (-2.59 to 8.66)	-3.85 (-10.9 to 3.87)	-4.29 (-11.3 to 3.30)	0.09	0.19	0.03
**Primary newborn outcome**									

© 2023 Ferrera A et al. *JAMA Network Open.*

Neonatal intensive care unit admission	-0.69 (-1.99 to 0.64)	-0.78 (-2.09 to 0.53)	0.92 (-2.77 to 4.78)	1.10 (-2.60 to 4.94)	1.37 (-3.69 to 6.77)	1.04 (-4.03 to 6.36)	0.19	0.34	0.98
**Secondary prenatal maternal outcomes**									
Gestational hypertension	0.52 (-1.06 to 2.15)	0.26 (-1.32 to 1.87)	3.40 (-0.82 to 7.83)	3.89 (-0.34 to 8.30)	-6.49 (-11.5 to -1.12)	-6.96 (-12.0 to -1.68)	0.03	<.001	<.001
Gestational diabetes	0.00 (-0.92 to 0.94)	-0.17 (-1.07 to 0.74)	-0.85 (-3.41 to 1.78)	-0.70 (-3.18 to 1.84)	1.81 (-1.87 to 5.66)	1.35 (-2.17 to 5.00)	0.58	0.25	0.19
Diagnosed depression	-0.38 (-1.70 to 0.97)	-0.34 (-1.65 to 0.99)	2.26 (-1.41 to 6.09)	2.29 (-1.36 to 6.08)	-1.55 (-6.30 to 3.49)	-1.83 (-6.55 to 3.13)	0.06	0.41	0.06
Moderate to severe depression [f]	-0.21 (-1.34 to 0.95)	-0.17 (-1.31 to 0.98)	-0.62 (-3.82 to 2.69)	-0.71 (-3.90 to 2.60)	1.57 (-3.04 to 6.45)	1.63 (-3.00 to 6.48)	0.67	0.3	0.26
Venous thromboembolism	-0.57 (-7.05 to 6.51)	-0.55 (-7.01 to 6.36)	-4.23 (-21.6 to 16.90)	-4.08 (-21.2 to 16.79)	18.35 (-8.78 to 57.82)	17.55 (-10.3 to 53.98)	0.63	0.1	0.09
**Secondary newborn outcome**									
Newborn Transient Tachypnea	-4.08 (-9.67 to 1.75)	-4.00 (-9.51 to 1.85)	5.59 (-11.1 to 26.18)	5.38 (-11.5 to 25.43)	13.73 (-8.11 to 43.06)	13.48 (-8.91 to 41.38)	0.16	0.04	0.46
Newborn 5-minute APGAR score <7	1.42 (-2.57 to 5.69)	1.22 (-2.80 to 5.40)	-9.55 (-19.9 to 1.88)	-9.44 (-19.6 to 2.05)	19.64 (1.04 to 43.21)	19.59 (0.51 to 42.29)	0.01	<.01	<.001
Birthweight small for gestational age	0.33 (-0.89 to 1.59)	0.28 (-0.95 to 1.52)	0.47 (-2.87 to 3.93)	0.54 (-2.78 to 3.98)	-2.70 (-7.18 to 2.03)	-2.53 (-7.01 to 2.16)	0.84	0.11	0.14
Birthweight large for gestational age	0.38 (-0.77 to 1.55)	0.31 (-0.82 to 1.46)	-2.06 (-5.18 to 1.18)	-1.69 (-4.79 to 1.51)	4.06 (-0.53 to 8.92)	3.19 (-1.33 to 7.93)	0.1	0.09	0.01

Individuals with delivery in T3 experienced the multimodal prenatal healthcare delivery for the entire duration of the pregnancy.
Interrupted time-series methodology for the trends in prenatal outcomes before and during the implementation of COVID-19 pandemic-related changes in prenatal healthcare used piecewise linear segmented regression, allowing a change in slope associated with time at two inflection points: 3/1/20 (initiation of changes in prenatal healthcare) and 12/6/20 (date which pregnancies were fully exposed to the prenatal healthcare changes). Log binomial regression was used to examine each binary outcome in relation to date of delivery (week), providing point and interval estimates of relative percent change in outcome probability associated with change in time, scaled to a 4-week change. Poisson regression was used for point and interval estimation of relative percent change in means for outcomes characterized as counts in relation to a 4-week change in delivery date.

[a] Adjusted for age, pre-pregnancy body mass index, neighborhood deprivation index and race/ethnicity.

[b] Two side Wald test adjusted for age, pre-pregnancy body mass index, neighborhood deprivation index and race/ethnicity. [c] Screening by 50-g, 1-h oral glucose challenge test; [d] Screening by Patient Health Questionnaire.

[e] Severe Maternal morbidity includes at least one of the following morbidities occurring at any time during pregnancy: acute myocardial infarction, aneurysm, acute renal failure, adult respiratory distress syndrome, amniotic fluid embolism, cardiac arrest/ventricular fibrillation, conversion of cardiac rhythm, disseminated intravascular coagulation, eclampsia, heart failure/arrest during surgery or procedure, puerperal cerebrovascular disorders, pulmonary edema/acute heart failure, severe anesthesia complications, sepsis, shock, sickle cell disease with crisis, air and thrombotic embolism, blood products transfusion, hysterectomy, temporary tracheostomy, or ventilation.[10-12]

[f] Patient Health Questionnaire screening scores (moderate to severe≥10).[13]

© 2023 Ferrera A et al. *JAMA Network Open.*

eTable 6. Interrupted Time Series Analysis for the Trends in Prenatal Visits, Care Process, and Health Outcomes in Association With Exposure to COVID-19 Pandemic–Related Implementation of a Multimodal Prenatal Health Care Delivery Among 145 940 Individuals Who **Lived in Urban Areas** and Delivered a Singleton Live Birth or Stillbirth, Kaiser Permanente Northern California, 2018-2021

	Percent Change (95% CI) per 4 weeks						P value [b]		
	**Unexposed T1** 7/1/18-2/29/20 deliveries		**Partially Exposed T2** 3/1/20-12/5/20 deliveries		**Fully Exposed T3** 12/6/20-10/31/21 deliveries				
	Unadjusted	Adjusted [a]	Unadjusted	Adjusted [a]	Unadjusted	Adjusted [a]	T2 vs. T1	T3 vs. T1	T3 vs. T2
**Prenatal care visits**									
Total prenatal in-office and telemedicine visits per individual	-0.28 (-0.31 to -0.24)	-0.29 (-0.32 to -0.26)	0.47 (0.38 to 0.56)	0.50 (0.40 to 0.59)	-0.47 (-0.59 to -0.34)	-0.53 (-0.66 to -0.40)	<.001	<.001	<.001
In-office prenatal visits per individual	-0.26 (-0.29 to -0.22)	-0.27 (-0.30 to -0.23)	-1.58 (-1.68 to -1.48)	-1.56 (-1.66 to -1.46)	2.77 (2.63 to 2.92)	2.72 (2.57 to 2.87)	<.001	<.001	<.001
Telemedicine prenatal visits per individual	-0.05 (-0.14 to 0.04)	-0.08 (-0.18 to 0.01)	9.80 (9.54 to 10.06)	9.86 (9.60 to 10.12)	-13.1 (-13.3 to -12.8)	-13.2 (-13.4 to -12.9)	<.001	<.001	<.001
Video prenatal visits per individual	16.33 (15.23 to 17.47)	16.23 (15.13 to 17.37)	18.76 (17.15 to 20.38)	19.02 (17.40 to 20.63)	-32.0 (-32.5 to -31.5)	-32.1 (-32.6 to -31.6)	<.001	<.001	<.001
Telephone prenatal visits per individual	0.06 (-0.03 to 0.16)	0.04 (-0.05 to 0.14)	4.51 (4.25 to 4.78)	4.54 (4.27 to 4.80)	-8.92 (-9.23 to -8.61)	-9.02 (-9.33 to -8.71)	<.001	<.001	<.001
**Prenatal care process**									
Blood pressure measurements per individual	-0.08 (-0.10 to -0.05)	-0.09 (-0.11 to -0.06)	-0.68 (-0.75 to -0.62)	-0.65 (-0.72 to -0.59)	1.33 (1.24 to 1.43)	1.22 (1.13 to 1.32)	<.001	<.001	<.001
Gestational diabetes screening [c]	0.01 (-0.02 to 0.04)	0.01 (-0.02 to 0.04)	-0.01 (-0.09 to 0.07)	-0.02 (-0.10 to 0.06)	0.06 (-0.05 to 0.18)	0.05 (-0.06 to 0.16)	0.32	0.37	0.17
Depression screening [d]	-0.02 (-0.06 to 0.01)	-0.02 (-0.06 to 0.02)	-0.25 (-0.36 to -0.14)	-0.26 (-0.36 to -0.15)	0.77 (0.61 to 0.92)	0.78 (0.63 to 0.92)	<.001	<.001	<.001
**Primary prenatal maternal outcomes**									
Pre-eclampsia/Eclampsia	0.78 (0.40 to 1.16)	0.74 (0.36 to 1.12)	-0.24 (-1.25 to 0.78)	-0.19 (-1.20 to 0.83)	-0.78 (-2.13 to 0.60)	-0.83 (-2.18 to 0.55)	0.02	<.01	0.3
Severe maternal morbidity [e]	0.15 (-0.37 to 0.68)	0.12 (-0.40 to 0.64)	0.28 (-1.13 to 1.72)	0.34 (-1.07 to 1.78)	1.18 (-0.73 to 3.13)	1.10 (-0.80 to 3.05)	0.68	0.17	0.38
Cesarean section	0.14 (-0.03 to 0.32)	0.05 (-0.12 to 0.22)	-0.01 (-0.49 to 0.47)	0.02 (-0.45 to 0.49)	0.00 (-0.65 to 0.66)	-0.09 (-0.73 to 0.56)	0.85	0.57	0.72
Preterm birth	0.34 (-0.00 to 0.68)	0.28 (-0.06 to 0.62)	-0.51 (-1.44 to 0.43)	-0.43 (-1.36 to 0.51)	-0.02 (-1.31 to 1.29)	-0.16 (-1.44 to 1.14)	0.05	0.37	0.64
Spontaneous preterm birth	-0.10 (-0.57 to 0.37)	-0.13 (-0.60 to 0.34)	-0.45 (-1.75 to 0.88)	-0.41 (-1.72 to 0.91)	0.47 (-1.37 to 2.36)	0.42 (-1.42 to 2.30)	0.58	0.43	0.31
Medically indicated preterm birth	0.88 (0.36 to 1.41)	0.77 (0.25 to 1.29)	-0.66 (-2.04 to 0.75)	-0.53 (-1.91 to 0.87)	-0.52 (-2.40 to 1.40)	-0.74 (-2.61 to 1.17)	0.02	0.03	0.81
**Primary newborn outcome**									
Neonatal intensive care unit admission	-0.17 (-0.49 to 0.14)	-0.22 (-0.54 to 0.09)	-0.83 (-1.71 to 0.06)	-0.79 (-1.67 to 0.10)	1.54 (0.27 to 2.83)	1.44 (0.18 to 2.73)	0.1	<.001	<.001

© 2023 Ferrera A et al. *JAMA Network Open.*

**Secondary prenatal maternal outcomes**									
Gestational hypertension	0.81 (0.47 to 1.16)	0.73 (0.39 to 1.07)	0.50 (-0.40 to 1.41)	0.61 (-0.29 to 1.51)	-1.26 (-2.43 to -0.08)	-1.41 (-2.57 to -0.24)	0.71	<.001	<.001
Gestational diabetes	-0.57 (-0.86 to -0.27)	-0.78 (-1.06 to -0.49)	0.70 (-0.15 to 1.56)	0.97 (0.14 to 1.81)	0.00 (-1.17 to 1.20)	-0.46 (-1.61 to 0.71)	<.001	0.46	<.01
Diagnosed depression	0.70 (0.49 to 0.92)	0.70 (0.49 to 0.91)	0.75 (0.18 to 1.32)	0.71 (0.15 to 1.27)	-0.87 (-1.60 to -0.14)	-0.92 (-1.63 to -0.20)	0.97	<.001	<.001
Moderate to severe depression [f]	0.23 (-0.08 to 0.53)	0.20 (-0.10 to 0.50)	-1.76 (-2.60 to -0.91)	-1.73 (-2.56 to -0.89)	2.59 (1.36 to 3.84)	2.60 (1.37 to 3.84)	<.001	<.001	<.001
Venous thromboembolism	0.07 (-1.61 to 1.79)	-0.03 (-1.71 to 1.68)	-2.30 (-6.90 to 2.52)	-2.21 (-6.82 to 2.62)	4.81 (-2.00 to 12.23)	4.62 (-2.24 to 11.95)	0.23	0.07	0.02
**Secondary newborn outcome**									
Newborn Transient Tachypnea	-1.27 (-2.93 to 0.42)	-1.28 (-2.94 to 0.41)	2.78 (-1.93 to 7.75)	2.74 (-1.98 to 7.69)	5.27 (-0.82 to 11.84)	5.23 (-0.90 to 11.74)	0.03	<.01	0.38
Newborn 5-minute APGAR score <7	-0.57 (-1.50 to 0.37)	-0.63 (-1.56 to 0.31)	1.44 (-1.18 to 4.13)	1.49 (-1.13 to 4.18)	0.79 (-2.68 to 4.41)	0.69 (-2.78 to 4.29)	0.04	0.31	0.62
Birthweight small for gestational age	0.07 (-0.24 to 0.39)	0.06 (-0.25 to 0.37)	0.21 (-0.66 to 1.08)	0.38 (-0.48 to 1.24)	-1.04 (-2.22 to 0.16)	-1.13 (-2.30 to 0.05)	0.34	<.01	<.01
Birthweight large for gestational age	-0.11 (-0.39 to 0.18)	-0.18 (-0.46 to 0.10)	0.32 (-0.47 to 1.11)	0.34 (-0.44 to 1.12)	-0.15 (-1.22 to 0.94)	-0.25 (-1.31 to 0.82)	0.08	0.86	0.22

Individuals with delivery in T3 experienced the multimodal prenatal healthcare delivery for the entire duration of the pregnancy.

Interrupted time-series methodology for the trends in prenatal outcomes before and during the implementation of COVID-19 pandemic-related changes in prenatal healthcare used piecewise linear segmented regression, allowing a change in slope associated with time at two inflection points: 3/1/20 (initiation of changes in prenatal healthcare) and 12/6/20 (date which pregnancies were fully exposed to the prenatal healthcare changes). Log binomial regression was used to examine each binary outcome in relation to date of delivery (week), providing point and interval estimates of relative percent change in outcome probability associated with change in time, scaled to a 4-week change. Poisson regression was used for point and interval estimation of relative percent change in means for outcomes characterized as counts in relation to a 4-week change in delivery date.

[a] Adjusted for age, pre-pregnancy body mass index, neighborhood deprivation index and race/ethnicity.

[b] Two side Wald test adjusted for age, pre-pregnancy body mass index, neighborhood deprivation index and race/ethnicity. [c] Screening by 50-g, 1-h oral glucose challenge test; [d] Screening by Patient Health Questionnaire.

[e] Severe Maternal morbidity includes at least one of the following morbidities occurring at any time during pregnancy: acute myocardial infarction, aneurysm, acute renal failure, adult respiratory distress syndrome, amniotic fluid embolism, cardiac arrest/ventricular fibrillation, conversion of cardiac rhythm, disseminated intravascular coagulation, eclampsia, heart failure/arrest during surgery or procedure, puerperal cerebrovascular disorders, pulmonary edema/acute heart failure, severe anesthesia complications, sepsis, shock, sickle cell disease with crisis, air and thrombotic embolism, blood products transfusion, hysterectomy, temporary tracheostomy, or ventilation.[10-12]

[f] Patient Health Questionnaire screening scores (moderate to severe≥10).[13]

© 2023 Ferrera A et al. *JAMA Network Open.*

eTable 7. Interrupted Time Series Analysis for the Trends in Prenatal Visits, Care Process, and Health Outcomes in Association With Exposure to COVID-19 Pandemic–Related Implementation of a Multimodal Prenatal Health Care Delivery Among 5305 Individuals Who **Lived in Rural Areas** and Delivered a Singleton Live Birth or Stillbirth, Kaiser Permanente Northern California, 2018-2021

	Percent Change (95% CI) per 4 weeks						P value [b]		
	**Unexposed T1** 7/1/18-2/29/20 deliveries		**Partially Exposed T2** 3/1/20-12/5/20 deliveries		**Fully Exposed T3** 12/6/20-10/31/21 deliveries				
	Unadjusted	Adjusted [a]	Unadjusted	Adjusted [a]	Unadjusted	Adjusted [a]	T2 vs. T1	T3 vs. T1	T3 vs. T2
**Prenatal care visits**									
Total prenatal in-office and telemedicine visits per individual	0.03 (-0.15 to 0.21)	-0.03 (-0.22 to 0.15)	-0.17 (-0.66 to 0.33)	-0.02 (-0.51 to 0.48)	-0.17 (-0.84 to 0.51)	-0.37 (-1.04 to 0.31)	0.92	0.19	0.25
In-office prenatal visits per individual	-0.07 (-0.26 to 0.12)	-0.13 (-0.32 to 0.06)	-1.50 (-2.03 to -0.97)	-1.35 (-1.88 to -0.81)	2.25 (1.48 to 3.02)	2.04 (1.27 to 2.81)	<.001	<.001	<.001
Telemedicine prenatal visits per individual	1.12 (0.57 to 1.67)	0.98 (0.43 to 1.53)	6.03 (4.64 to 7.44)	6.31 (4.91 to 7.74)	-10.8 (-12.2 to -9.35)	-11.2 (-12.6 to -9.73)	<.001	<.001	<.001
Video prenatal visits per individual	13.91 (7.24 to 22.59)	13.56 (6.92 to 22.20)	26.39 (14.48 to 38.49)	26.95 (14.95 to 39.19)	-34.8 (-38.1 to -31.5)	-35.0 (-38.3 to -31.6)	<.01	<.001	<.001
Telephone prenatal visits per individual	1.33 (0.77 to 1.88)	1.18 (0.62 to 1.74)	1.69 (0.29 to 3.12)	2.01 (0.59 to 3.45)	-7.38 (-9.07 to -5.65)	-7.87 (-9.56 to -6.14)	0.13	<.001	<.001
**Prenatal care process**									
Blood pressure measurements per individual	0.05 (-0.08 to 0.18)	-0.05 (-0.18 to 0.08)	-1.49 (-1.84 to -1.14)	-1.18 (-1.54 to -0.83)	2.97 (2.46 to 3.48)	2.44 (1.93 to 2.95)	<.001	<.001	<.001
Gestational diabetes screening [c]	0.07 (-0.12 to 0.26)	0.10 (-0.08 to 0.29)	-0.23 (-0.76 to 0.30)	-0.35 (-0.87 to 0.17)	0.56 (-0.19 to 1.33)	0.62 (-0.14 to 1.39)	0.02	0.07	<.01
Depression screening [d]	0.16 (-0.04 to 0.36)	0.18 (-0.02 to 0.37)	-0.59 (-1.14 to -0.03)	-0.68 (-1.22 to -0.13)	1.19 (0.42 to 1.97)	1.24 (0.46 to 2.02)	<.001	<.001	<.001
**Primary prenatal maternal outcomes**									
Pre-eclampsia/Eclampsia	1.52 (-0.69 to 3.83)	1.49 (-0.74 to 3.77)	-0.73 (-6.19 to 5.10)	-0.61 (-6.08 to 5.17)	0.90 (-6.13 to 8.59)	0.65 (-6.37 to 8.20)	0.34	0.76	0.7
Severe maternal morbidity [e]	1.03 (-1.93 to 4.15)	0.97 (-2.01 to 4.05)	0.00 (-7.48 to 8.17)	0.20 (-7.30 to 8.30)	-0.98 (-10.5 to 9.82)	-1.22 (-10.8 to 9.39)	0.8	0.57	0.76
Cesarean section	0.07 (-0.88 to 1.04)	0.02 (-0.92 to 0.97)	-0.67 (-3.23 to 1.95)	-0.55 (-3.06 to 2.02)	0.81 (-2.71 to 4.49)	0.27 (-3.20 to 3.87)	0.56	0.85	0.6
Preterm birth	-1.07 (-2.96 to 0.88)	-1.03 (-2.92 to 0.89)	0.91 (-4.43 to 6.59)	1.13 (-4.19 to 6.75)	1.52 (-5.75 to 9.50)	0.99 (-6.24 to 8.79)	0.3	0.46	0.97
Spontaneous preterm birth	-1.53 (-4.23 to 1.27)	-1.41 (-4.11 to 1.37)	0.89 (-6.85 to 9.32)	1.03 (-6.70 to 9.41)	2.10 (-8.58 to 14.35)	1.75 (-8.95 to 13.71)	0.42	0.45	0.89
Medically indicated preterm birth	-0.60 (-3.34 to 2.25)	-0.66 (-3.39 to 2.16)	0.85 (-6.63 to 9.00)	1.05 (-6.41 to 9.10)	1.03 (-8.90 to 12.32)	0.56 (-9.36 to 11.56)	0.56	0.75	0.92
**Primary newborn outcome**									

© 2023 Ferrera A et al. *JAMA Network Open.*

Neonatal intensive care unit admission	-0.05 (-1.70 to 1.64)	-0.07 (-1.71 to 1.59)	-3.99 (-8.50 to 0.73)	-3.95 (-8.42 to 0.73)	6.94 (-0.12 to 14.67)	6.83 (-0.24 to 14.42)	0.03	<.01	<.001
**Secondary prenatal maternal outcomes**									
Gestational hypertension	1.49 (-0.29 to 3.34)	1.41 (-0.38 to 3.23)	-1.71 (-6.13 to 2.94)	-1.34 (-5.76 to 3.29)	2.43 (-3.49 to 8.80)	1.49 (-4.36 to 7.70)	0.12	0.97	0.3
Gestational diabetes	0.86 (-1.19 to 2.99)	0.88 (-1.16 to 2.97)	-0.77 (-6.02 to 4.80)	-1.18 (-6.20 to 4.11)	0.32 (-6.60 to 7.88)	0.11 (-6.46 to 7.14)	0.31	0.76	0.68
Diagnosed depression	0.81 (-0.30 to 1.94)	0.78 (-0.33 to 1.90)	0.65 (-2.18 to 3.57)	0.87 (-1.95 to 3.78)	-0.39 (-3.93 to 3.33)	-0.99 (-4.51 to 2.67)	0.93	0.2	0.26
Moderate to severe depression [f]	-0.04 (-1.84 to 1.83)	0.05 (-1.76 to 1.90)	-0.65 (-5.52 to 4.49)	-0.60 (-5.43 to 4.49)	0.10 (-6.61 to 7.42)	-0.07 (-6.75 to 7.08)	0.73	0.96	0.86
Venous thromboembolism	-3.78 (-11.8 to 4.87)	-4.05 (-11.9 to 4.48)	10.27 (-12.8 to 41.55)	11.23 (-12.3 to 41.15)	-4.81 (-29.6 to 30.75)	-6.31 (-30.8 to 26.91)	0.11	0.83	0.22
**Secondary newborn outcome**									
Newborn Transient Tachypnea	-1.00 (-10.8 to 10.20)	-0.70 (-10.6 to 10.26)	-5.28 (-30.5 to 28.91)	-5.72 (-30.5 to 27.92)	23.66 (-15.6 to 94.62)	23.65 (-17.9 to 86.14)	0.66	0.15	0.14
Newborn 5-minute APGAR score <7	1.79 (-3.60 to 7.75)	1.83 (-3.65 to 7.63)	-9.60 (-23.1 to 5.82)	-9.49 (-22.8 to 6.06)	14.36 (-8.77 to 45.87)	13.85 (-9.79 to 43.68)	0.05	0.2	0.02
Birthweight small for gestational age	0.37 (-1.64 to 2.45)	0.43 (-1.58 to 2.49)	-0.73 (-6.03 to 4.89)	-0.75 (-6.02 to 4.81)	-0.08 (-7.25 to 7.77)	0.31 (-6.90 to 8.08)	0.57	0.96	0.75
Birthweight large for gestational age	0.00 (-1.41 to 1.45)	-0.21 (-1.61 to 1.21)	0.95 (-2.82 to 4.88)	1.42 (-2.31 to 5.30)	-1.91 (-6.78 to 3.28)	-3.00 (-7.76 to 2.00)	0.26	0.13	0.05

Individuals with delivery in T3 experienced the multimodal prenatal healthcare delivery for the entire duration of the pregnancy.

Interrupted time-series methodology for the trends in prenatal outcomes before and during the implementation of COVID-19 pandemic-related changes in prenatal healthcare used piecewise linear segmented regression, allowing a change in slope associated with time at two inflection points: 3/1/20 (initiation of changes in prenatal healthcare) and 12/6/20 (date which pregnancies were fully exposed to the prenatal healthcare changes). Log binomial regression was used to examine each binary outcome in relation to date of delivery (week), providing point and interval estimates of relative percent change in outcome probability associated with change in time, scaled to a 4-week change. Poisson regression was used for point and interval estimation of relative percent change in means for outcomes characterized as counts in relation to a 4-week change in delivery date.

[a] Adjusted for age, pre-pregnancy body mass index, neighborhood deprivation index and race/ethnicity.

[b] Two side Wald test adjusted for age, pre-pregnancy body mass index, neighborhood deprivation index and race/ethnicity. [c] Screening by 50-g, 1-h oral glucose challenge test; [d] Screening by Patient Health Questionnaire.

[e] Severe Maternal morbidity includes at least one of the following morbidities occurring at any time during pregnancy: acute myocardial infarction, aneurysm, acute renal failure, adult respiratory distress syndrome, amniotic fluid embolism, cardiac arrest/ventricular fibrillation, conversion of cardiac rhythm, disseminated intravascular coagulation, eclampsia, heart failure/arrest during surgery or procedure, puerperal cerebrovascular disorders, pulmonary edema/acute heart failure, severe anesthesia complications, sepsis, shock, sickle cell disease with crisis, air and thrombotic embolism, blood products transfusion, hysterectomy, temporary tracheostomy, or ventilation.[10-12]

[f] Patient Health Questionnaire screening scores (moderate to severe≥10).[13]

© 2023 Ferrera A et al. *JAMA Network Open.*

**eTable 8**. Interrupted Time Series Analysis for the Trends in Prenatal Visits, Care Process, and Health Outcomes in Association With Exposure to COVID-19 Pandemic–Related Implementation of a Multimodal Prenatal Health Care Delivery Among 39 114 **Asian and Pacific Islander** Individuals Who Delivered a Singleton Live Birth or Stillbirth, Kaiser Permanente Northern California, 2018-2021									
	Percent Change (95% CI) per 4 weeks						P value [b]		
	**Unexposed** 7/1/18-2/29/20 T1		**Partially Exposed** 3/1/20-12/5/20 T2		**Fully Exposed** 12/6/20-10/31/21 T3				
	Unadjusted	Adjusted [a]	Unadjusted	Adjusted [a]	Unadjusted	Adjusted [a]	T2 vs. T1	T3 vs. T1	T3 vs. T2
**Prenatal care visits**									
Total prenatal in-office and virtual clinic visits per individual	-0.21 (-0.27 to -0.14)	-0.23 (-0.29 to -0.17)	0.35 (0.17 to 0.52)	0.38 (0.21 to 0.55)	-0.33 (-0.57 to -0.09)	-0.38 (-0.62 to -0.14)	<.001	0.1	<.001
In office prenatal clinic visits per individual	-0.17 (-0.24 to -0.10)	-0.19 (-0.26 to -0.12)	-1.89 (-2.08 to -1.70)	-1.86 (-2.05 to -1.67)	3.12 (2.83 to 3.40)	3.08 (2.80 to 3.37)	<.001	<.001	<.001
Telemedicine prenatal clinic visits (video or telephone) per individual	-0.10 (-0.28 to 0.07)	-0.14 (-0.31 to 0.03)	9.96 (9.48 to 10.44)	10.02 (9.54 to 10.51)	-12.9 (-13.3 to -12.4)	-13.0 (-13.4 to -12.5)	<.001	<.001	<.001
Video prenatal visits per individual	14.46 (12.74 to 16.29)	14.35 (12.63 to 16.17)	20.08 (17.37 to 22.81)	20.35 (17.63 to 23.09)	-30.7 (-31.6 to -29.8)	-30.8 (-31.7 to -29.9)	<.001	<.001	<.001
Telephone prenatal visits per individual	-0.00 (-0.18 to 0.17)	-0.02 (-0.20 to 0.15)	4.44 (3.94 to 4.93)	4.44 (3.95 to 4.94)	-9.14 (-9.72 to -8.56)	-9.23 (-9.81 to -8.65)	<.001	<.001	<.001
**Prenatal care process**									
Blood pressure measurements per individual	-0.11 (-0.15 to -0.06)	-0.12 (-0.16 to -0.07)	-0.65 (-0.77 to -0.52)	-0.64 (-0.77 to -0.51)	1.29 (1.11 to 1.47)	1.21 (1.02 to 1.39)	<.001	<.001	<.001
Gestational diabetes screening[c]	0.04 (-0.01 to 0.09)	0.04 (-0.01 to 0.09)	-0.04 (-0.18 to 0.09)	-0.06 (-0.19 to 0.08)	0.05 (-0.15 to 0.24)	0.06 (-0.12 to 0.24)	0.07	0.77	0.17
Depression screening [d]	-0.05 (-0.12 to 0.02)	-0.05 (-0.12 to 0.02)	-0.09 (-0.29 to 0.12)	-0.09 (-0.29 to 0.11)	0.51 (0.23 to 0.80)	0.51 (0.23 to 0.80)	0.6	<.001	<.001
**Primary maternal outcomes**									
Pre-eclampsia/Eclampsia	0.20 (-0.53 to 0.94)	0.18 (-0.55 to 0.92)	0.43 (-1.56 to 2.47)	0.41 (-1.58 to 2.45)	-0.55 (-3.24 to 2.23)	-0.59 (-3.28 to 2.18)	0.77	0.45	0.41
Severe maternal morbidity [e]	-0.66 (-1.61 to 0.31)	-0.66 (-1.62 to 0.30)	3.23 (0.54 to 5.99)	3.22 (0.53 to 5.97)	-2.24 (-5.60 to 1.27)	-2.30 (-5.67 to 1.19)	<.001	0.21	<.001
Cesarean section	-0.07 (-0.40 to 0.26)	-0.14 (-0.46 to 0.18)	0.37 (-0.53 to 1.29)	0.41 (-0.48 to 1.31)	-0.50 (-1.74 to 0.76)	-0.66 (-1.88 to 0.58)	0.11	0.26	0.05
Preterm birth	0.29 (-0.36 to 0.95)	0.28 (-0.37 to 0.94)	-0.24 (-2.01 to 1.56)	-0.28 (-2.04 to 1.52)	0.02 (-2.41 to 2.54)	-0.16 (-2.59 to 2.33)	0.41	0.63	0.91
Spontaneous preterm birth	-0.12 (-0.99 to 0.76)	-0.10 (-0.97 to 0.78)	-0.55 (-2.96 to 1.93)	-0.60 (-3.02 to 1.87)	0.77 (-2.66 to 4.35)	0.73 (-2.70 to 4.29)	0.59	0.52	0.38
Medically indicated preterm birth	0.86 (-0.17 to 1.90)	0.79 (-0.24 to 1.83)	0.00 (-2.70 to 2.78)	0.04 (-2.65 to 2.81)	-0.82 (-4.40 to 2.92)	-1.17 (-4.73 to 2.53)	0.48	0.15	0.46
**Primary newborn outcomes**									
Neonatal intensive care unit admission	-0.78 (-1.41 to -0.15)	-0.78 (-1.40 to -0.15)	1.32 (-0.47 to 3.14)	1.23 (-0.56 to 3.04)	-0.53 (-2.97 to 1.98)	-0.67 (-3.10 to 1.81)	<.01	0.91	0.08
**Secondary maternal outcomes**									

© 2023 Ferrera A et al. *JAMA Network Open.*

Gestational hypertension	1.45 (0.73 to 2.18)	1.40 (0.68 to 2.12)	-0.49 (-2.32 to 1.38)	-0.49 (-2.31 to 1.37)	-0.48 (-2.88 to 1.99)	-0.64 (-3.02 to 1.81)	<.01	0.03	0.89
Gestational diabetes	-0.76 (-1.19 to -0.32)	-0.86 (-1.29 to -0.44)	0.98 (-0.28 to 2.25)	0.98 (-0.25 to 2.22)	0.04 (-1.70 to 1.82)	-0.22 (-1.92 to 1.50)	<.001	0.31	0.12
Diagnosed depression	1.15 (0.50 to 1.80)	1.12 (0.48 to 1.77)	-0.18 (-1.83 to 1.50)	-0.22 (-1.87 to 1.46)	1.31 (-0.84 to 3.53)	1.29 (-0.86 to 3.49)	0.04	0.84	0.13
Moderate to severe depression [f]	0.11 (-0.52 to 0.74)	0.17 (-0.45 to 0.80)	-2.03 (-3.76 to -0.26)	-2.14 (-3.87 to -0.38)	3.44 (0.86 to 6.11)	3.58 (0.99 to 6.24)	<.001	<.001	<.001
Venous thromboembolism	-1.18 (-5.36 to 3.24)	-1.22 (-5.41 to 3.16)	0.75 (-10.9 to 14.07)	0.77 (-10.9 to 13.97)	6.29 (-9.63 to 26.00)	6.05 (-10.1 to 25.13)	0.67	0.25	0.49
**Primary newborn outcomes**									
Transient Tachypnea	-1.33 (-4.74 to 2.22)	-1.23 (-4.65 to 2.30)	0.22 (-9.32 to 10.86)	-0.04 (-9.57 to 10.49)	10.50 (-2.96 to 26.54)	10.71 (-3.02 to 26.38)	0.75	0.02	0.09
5-minute APGAR score <7	0.83 (-1.11 to 2.82)	0.86 (-1.08 to 2.85)	-5.51 (-10.6 to -0.16)	-5.65 (-10.7 to -0.29)	12.76 (4.53 to 21.88)	12.82 (4.48 to 21.82)	<.01	<.001	<.001
Small for gestational age	-0.28 (-0.77 to 0.22)	-0.27 (-0.76 to 0.23)	1.58 (0.21 to 2.97)	1.64 (0.27 to 3.03)	-2.42 (-4.23 to -0.58)	-2.38 (-4.19 to -0.54)	<.001	<.01	<.001
Large for gestational age	0.13 (-0.58 to 0.84)	0.07 (-0.63 to 0.78)	-1.33 (-3.27 to 0.65)	-1.34 (-3.27 to 0.62)	2.45 (-0.35 to 5.36)	2.16 (-0.62 to 5.02)	0.06	0.04	<.01

Individuals with delivery in T3 experienced the multimodal prenatal healthcare delivery for the entire duration of the pregnancy.

Interrupted time-series methodology for the trends in prenatal outcomes before and during the implementation of COVID-19 pandemic-related changes in prenatal healthcare used piecewise linear segmented regression, allowing a change in slope associated with time at two inflection points: 3/1/20 (initiation of changes in prenatal healthcare) and 12/6/20 (date which pregnancies were fully exposed to the prenatal healthcare changes). Log binomial regression was used to examine each binary outcome in relation to date of delivery (week), providing point and interval estimates of relative percent change in outcome probability associated with change in time, scaled to a 4-week change. Poisson regression was used for point and interval estimation of relative percent change in means for outcomes characterized as counts in relation to a 4-week change in delivery date.

[a] Adjusted for age, pre-pregnancy body mass index and neighborhood deprivation index.

[b] Two side Wald test adjusted for age, pre-pregnancy body mass index and neighborhood deprivation index. [c] Screening by 50-g, 1-h oral glucose challenge test; [d] Screening by Patient Health Questionnaire.

[e] Severe Maternal morbidity includes at least one of the following morbidities occurring at any time during pregnancy: acute myocardial infarction, aneurysm, acute renal failure, adult respiratory distress syndrome, amniotic fluid embolism, cardiac arrest/ventricular fibrillation, conversion of cardiac rhythm, disseminated intravascular coagulation, eclampsia, heart failure/arrest during surgery or procedure, puerperal cerebrovascular disorders, pulmonary edema/acute heart failure, severe anesthesia complications, sepsis, shock, sickle cell disease with crisis, air and thrombotic embolism, blood products transfusion, hysterectomy, temporary tracheostomy, or ventilation.[10-12]

[f] Patient Health Questionnaire screening scores (moderate to severe≥10).[13]

© 2023 Ferrera A et al. *JAMA Network Open.*

eTable 9. Interrupted Time Series Analysis for the Trends in Prenatal Visits, Care Process, and Health Outcomes in Association With Exposure to COVID-19 Pandemic–Related Implementation of a Multimodal Prenatal Health Care Delivery Among 10 013 **Black** Individuals Who Delivered a Singleton Live Birth or Stillbirth, Kaiser Permanente Northern California, 2018-2021

	Percent Change (95% CI) per 4 weeks						P value [b]		
	**Unexposed** 7/1/18-2/29/20 T1		**Partially Exposed** 3/1/20-12/5/20 T2		**Fully Exposed** 12/6/20-10/31/21 T3				
	Unadjusted	Adjusted [a]	Unadjusted	Adjusted [a]	Unadjusted	Adjusted [a]	T2 vs. T1	T3 vs. T1	T3 vs. T2
**Process care visits**									
Total prenatal in-office and virtual clinic visits per individual	-0.33 (-0.46 to -0.21)	-0.34 (-0.47 to -0.22)	0.46 (0.11 to 0.81)	0.52 (0.17 to 0.87)	-0.39 (-0.86 to 0.09)	-0.49 (-0.97 to -0.02)	<.001	0.39	<.001
In office prenatal clinic visits per individual	-0.27 (-0.41 to -0.14)	-0.28 (-0.41 to -0.15)	-1.67 (-2.05 to -1.30)	-1.61 (-1.99 to -1.24)	2.90 (2.35 to 3.46)	2.79 (2.24 to 3.35)	<.001	<.001	<.001
Telemedicine prenatal clinic visits (video or telephone) per individual	-0.44 (-0.78 to -0.10)	-0.43 (-0.78 to -0.09)	10.15 (9.20 to 11.12)	10.15 (9.20 to 11.12)	-13.0 (-13.9 to -12.0)	-13.0 (-13.9 to -12.1)	<.001	<.001	<.001
Video prenatal visits per individual	15.57 (11.67 to 20.06)	15.45 (11.56 to 19.94)	18.50 (12.39 to 24.67)	18.77 (12.64 to 24.97)	-32.2 (-34.1 to -30.2)	-32.3 (-34.3 to -30.3)	0.21	<.001	<.001
Telephone prenatal visits per individual	-0.38 (-0.73 to -0.04)	-0.36 (-0.71 to -0.01)	5.85 (4.88 to 6.83)	5.83 (4.86 to 6.81)	-9.06 (-10.2 to -7.95)	-9.09 (-10.2 to -7.99)	<.001	<.001	<.001
**Prenatal care process**									
Blood pressure measurements per individual	-0.12 (-0.20 to -0.03)	-0.12 (-0.21 to -0.04)	-0.73 (-0.97 to -0.50)	-0.71 (-0.95 to -0.48)	1.91 (1.57 to 2.25)	1.84 (1.51 to 2.18)	<.001	<.001	<.001
Gestational diabetes screening[c]	0.03 (-0.11 to 0.18)	0.03 (-0.11 to 0.17)	0.03 (-0.36 to 0.41)	0.00 (-0.37 to 0.37)	0.11 (-0.41 to 0.63)	0.11 (-0.39 to 0.62)	0.84	0.67	0.62
Depression screening [d]	0.12 (-0.06 to 0.29)	0.12 (-0.06 to 0.29)	-0.35 (-0.83 to 0.14)	-0.36 (-0.83 to 0.12)	0.72 (0.05 to 1.39)	0.73 (0.07 to 1.40)	<.01	0.01	<.001
**Primary maternal outcomes**									
Pre-eclampsia/Eclampsia	1.57 (0.25 to 2.92)	1.55 (0.22 to 2.89)	-1.72 (-5.04 to 1.73)	-1.65 (-4.98 to 1.79)	0.15 (-4.38 to 4.95)	0.10 (-4.44 to 4.86)	0.02	0.41	0.4
Severe maternal morbidity [e]	2.30 (0.60 to 4.05)	2.30 (0.59 to 4.04)	-6.42 (-10.6 to -2.06)	-6.50 (-10.7 to -2.15)	8.99 (2.40 to 16.14)	8.96 (2.31 to 16.03)	<.001	<.01	<.001
Cesarean section	0.47 (-0.13 to 1.08)	0.38 (-0.21 to 0.97)	-0.34 (-1.95 to 1.30)	-0.29 (-1.85 to 1.30)	-0.22 (-2.40 to 2.02)	-0.23 (-2.37 to 1.95)	0.27	0.45	0.95
Preterm birth	0.45 (-0.65 to 1.56)	0.45 (-0.64 to 1.56)	-0.14 (-3.06 to 2.87)	-0.20 (-3.11 to 2.81)	-0.53 (-4.45 to 3.59)	-0.59 (-4.52 to 3.50)	0.57	0.49	0.83
Spontaneous preterm birth	0.35 (-1.21 to 1.96)	0.40 (-1.17 to 2.00)	0.44 (-3.75 to 4.83)	0.37 (-3.82 to 4.75)	-1.93 (-7.48 to 4.02)	-1.87 (-7.44 to 4.03)	0.98	0.29	0.39
Medically indicated preterm birth	0.54 (-1.06 to 2.19)	0.50 (-1.10 to 2.14)	-0.74 (-5.00 to 3.73)	-0.77 (-5.02 to 3.68)	0.92 (-4.90 to 7.19)	0.72 (-5.12 to 6.91)	0.45	0.92	0.58
**Primary newborn outcomes**									
Neonatal intensive care unit admission	-0.29 (-1.30 to 0.74)	-0.34 (-1.34 to 0.68)	-1.16 (-3.99 to 1.75)	-1.10 (-3.93 to 1.80)	2.79 (-1.29 to 7.08)	2.68 (-1.42 to 6.94)	0.49	0.05	0.04
**Secondary maternal outcomes**									

© 2023 Ferrera A et al. *JAMA Network Open.*

Gestational hypertension	1.80 (0.52 to 3.10)	1.74 (0.46 to 3.04)	-1.33 (-4.51 to 1.97)	-1.25 (-4.43 to 2.04)	-0.01 (-4.21 to 4.41)	-0.06 (-4.27 to 4.34)	0.02	0.27	0.54
Gestational diabetes	-0.90 (-2.36 to 0.58)	-1.27 (-2.69 to 0.17)	2.28 (-1.88 to 6.63)	2.89 (-1.23 to 7.19)	-2.85 (-8.29 to 2.98)	-3.43 (-8.81 to 2.27)	<.01	0.3	0.01
Diagnosed depression	0.16 (-0.55 to 0.87)	0.18 (-0.52 to 0.89)	1.62 (-0.27 to 3.56)	1.55 (-0.34 to 3.47)	-1.68 (-4.07 to 0.79)	-1.63 (-4.02 to 0.82)	0.06	0.05	<.01
Moderate to severe depression [f]	-0.08 (-0.93 to 0.77)	-0.07 (-0.92 to 0.78)	-0.82 (-3.16 to 1.59)	-0.77 (-3.10 to 1.62)	0.77 (-2.57 to 4.27)	0.76 (-2.59 to 4.21)	0.44	0.51	0.31
Venous thromboembolism	1.82 (-3.26 to 7.41)	1.61 (-3.55 to 7.05)	-0.90 (-13.6 to 13.75)	-0.58 (-13.3 to 14.02)	-7.67 (-24.3 to 12.68)	-8.16 (-24.6 to 11.90)	0.68	0.17	0.36
**Secondary newborn outcomes**									
Transient Tachypnea	-3.71 (-9.75 to 2.67)	-3.79 (-9.72 to 2.53)	8.63 (-9.19 to 30.98)	8.58 (-9.45 to 30.21)	2.25 (-18.1 to 29.34)	2.25 (-18.6 to 28.39)	0.08	0.48	0.57
5-minute APGAR score <7	-2.36 (-4.91 to 0.27)	-2.38 (-4.93 to 0.24)	5.77 (-1.83 to 14.05)	5.80 (-1.84 to 14.03)	-2.13 (-11.3 to 8.20)	-2.32 (-11.5 to 7.88)	<.01	0.99	0.08
Small for gestational age	0.48 (-0.55 to 1.53)	0.61 (-0.43 to 1.65)	-0.43 (-3.19 to 2.43)	-0.58 (-3.34 to 2.25)	-0.88 (-4.65 to 3.08)	-0.78 (-4.55 to 3.13)	0.27	0.34	0.91
Large for gestational age	0.47 (-0.71 to 1.68)	0.28 (-0.89 to 1.48)	-0.01 (-3.19 to 3.28)	0.29 (-2.87 to 3.56)	-1.88 (-6.14 to 2.62)	-2.13 (-6.39 to 2.32)	>.99	0.14	0.22

Individuals with delivery in T3 experienced the multimodal prenatal healthcare delivery for the entire duration of the pregnancy.
Interrupted time-series methodology for the trends in prenatal outcomes before and during the implementation of COVID-19 pandemic-related changes in prenatal healthcare used piecewise linear segmented regression, allowing a change in slope associated with time at two inflection points: 3/1/20 (initiation of changes in prenatal healthcare) and 12/6/20 (date which pregnancies were fully exposed to the prenatal healthcare changes). Log binomial regression was used to examine each binary outcome in relation to date of delivery (week), providing point and interval estimates of relative percent change in outcome probability associated with change in time, scaled to a 4-week change. Poisson regression was used for point and interval estimation of relative percent change in means for outcomes characterized as counts in relation to a 4-week change in delivery date.

[a] Adjusted for age, pre-pregnancy body mass index and neighborhood deprivation index.

[b] Two side Wald test adjusted for age, pre-pregnancy body mass index and neighborhood deprivation index. [c] Screening by 50-g, 1-h oral glucose challenge test; [d] Screening by Patient Health Questionnaire.

[e] Severe Maternal morbidity includes at least one of the following morbidities occurring at any time during pregnancy: acute myocardial infarction, aneurysm, acute renal failure, adult respiratory distress syndrome, amniotic fluid embolism, cardiac arrest/ventricular fibrillation, conversion of cardiac rhythm, disseminated intravascular coagulation, eclampsia, heart failure/arrest during surgery or procedure, puerperal cerebrovascular disorders, pulmonary edema/acute heart failure, severe anesthesia complications, sepsis, shock, sickle cell disease with crisis, air and thrombotic embolism, blood products transfusion, hysterectomy, temporary tracheostomy, or ventilation.[10-12]

[f] Patient Health Questionnaire screening scores (moderate to severe≥10).[13]

© 2023 Ferrera A et al. *JAMA Network Open.*

eTable 10. Interrupted Time Series Analysis for the Trends in Prenatal Visits, Care Process, and Health Outcomes in Association With Exposure to COVID-19 Pandemic–Related Implementation of a Multimodal Prenatal Health Care Delivery Among 42 245 **Hispanic** Individuals Who Delivered a Singleton Live Birth or Stillbirth, Kaiser Permanente Northern California, 2018-2021

	Percent Change (95% CI) per 4 weeks						P value [b]		
	**Unexposed** 7/1/18-2/29/20 T1		**Partially Exposed** 3/1/20-12/5/20 T2		**Fully Exposed** 12/6/20-10/31/21 T3				
	Unadjusted	Adjusted [a]	Unadjusted	Adjusted [a]	Unadjusted	Adjusted [a]	T2 vs. T1	T3 vs. T1	T3 vs. T2
**Prenatal care process**									
Total prenatal in-office and virtual clinic visits per individual	-0.30 (-0.36 to -0.24)	-0.29 (-0.35 to -0.23)	0.50 (0.33 to 0.67)	0.49 (0.32 to 0.66)	-0.63 (-0.86 to -0.40)	-0.69 (-0.92 to -0.45)	<.001	<.001	<.001
In office prenatal clinic visits per individual	-0.27 (-0.34 to -0.20)	-0.26 (-0.32 to -0.19)	-1.43 (-1.62 to -1.25)	-1.44 (-1.62 to -1.26)	2.39 (2.12 to 2.66)	2.34 (2.07 to 2.61)	<.001	<.001	<.001
Telemedicine prenatal clinic visits (video or telephone) per individual	-0.19 (-0.37 to -0.02)	-0.19 (-0.36 to -0.01)	9.44 (8.96 to 9.93)	9.43 (8.95 to 9.91)	-12.6 (-13.1 to -12.2)	-12.7 (-13.2 to -12.3)	<.001	<.001	<.001
Video prenatal visits per individual	18.08 (15.43 to 21.00)	18.05 (15.39 to 20.97)	18.45 (14.66 to 22.23)	18.58 (14.78 to 22.37)	-32.8 (-33.9 to -31.7)	-32.9 (-34.0 to -31.9)	0.75	<.001	<.001
Telephone prenatal visits per individual	-0.07 (-0.25 to 0.10)	-0.07 (-0.25 to 0.11)	5.28 (4.79 to 5.77)	5.26 (4.77 to 5.75)	-9.44 (-9.98 to -8.89)	-9.51 (-10.1 to -8.96)	<.001	<.001	<.001
**Prenatal care visits**									
Blood pressure measurements per individual	-0.08 (-0.13 to -0.04)	-0.08 (-0.13 to -0.04)	-0.68 (-0.80 to -0.56)	-0.65 (-0.77 to -0.53)	1.32 (1.15 to 1.49)	1.21 (1.04 to 1.39)	<.001	<.001	<.001
Gestational diabetes screening[c]	-0.01 (-0.07 to 0.05)	-0.01 (-0.07 to 0.05)	0.10 (-0.06 to 0.27)	0.08 (-0.08 to 0.24)	-0.09 (-0.31 to 0.13)	-0.08 (-0.29 to 0.14)	0.15	0.38	0.1
Depression screening [d]	-0.02 (-0.08 to 0.05)	-0.01 (-0.08 to 0.06)	-0.20 (-0.39 to -0.01)	-0.22 (-0.41 to -0.03)	0.73 (0.47 to 0.99)	0.75 (0.49 to 1.00)	<.01	<.001	<.001
**Primary maternal outcomes**									
Pre-eclampsia/Eclampsia	0.56 (-0.12 to 1.26)	0.51 (-0.18 to 1.20)	0.44 (-1.37 to 2.29)	0.57 (-1.24 to 2.42)	-1.03 (-3.38 to 1.39)	-1.13 (-3.47 to 1.28)	0.93	0.07	0.12
Severe maternal morbidity [e]	0.62 (-0.37 to 1.62)	0.58 (-0.40 to 1.58)	0.11 (-2.47 to 2.77)	0.19 (-2.40 to 2.85)	1.34 (-2.03 to 4.85)	1.28 (-2.10 to 4.77)	0.7	0.59	0.49
Cesarean section	0.11 (-0.23 to 0.44)	0.06 (-0.27 to 0.39)	0.21 (-0.70 to 1.13)	0.12 (-0.77 to 1.02)	-0.36 (-1.58 to 0.87)	-0.34 (-1.54 to 0.86)	0.85	0.37	0.39
Preterm birth	0.19 (-0.43 to 0.82)	0.15 (-0.46 to 0.78)	-0.32 (-2.00 to 1.40)	-0.24 (-1.92 to 1.48)	-0.17 (-2.46 to 2.19)	-0.29 (-2.58 to 2.06)	0.55	0.61	0.96
Spontaneous preterm birth	-0.21 (-1.07 to 0.67)	-0.22 (-1.09 to 0.65)	-0.17 (-2.56 to 2.28)	-0.14 (-2.53 to 2.31)	0.62 (-2.67 to 4.05)	0.56 (-2.74 to 3.97)	0.92	0.53	0.64
Medically indicated preterm birth	0.65 (-0.28 to 1.59)	0.60 (-0.33 to 1.53)	-0.52 (-2.98 to 2.01)	-0.43 (-2.89 to 2.09)	-0.97 (-4.28 to 2.48)	-1.11 (-4.42 to 2.31)	0.29	0.18	0.65
**Primary newborn outcomes**									
Neonatal intensive care unit admission	-0.11 (-0.71 to 0.49)	-0.15 (-0.75 to 0.45)	-1.35 (-3.00 to 0.33)	-1.28 (-2.93 to 0.39)	2.73 (0.36 to 5.17)	2.60 (0.24 to 5.02)	0.08	<.01	<.001
**Secondary maternal outcomes**									

© 2023 Ferrera A et al. *JAMA Network Open.*

Gestational hypertension	0.08 (-0.60 to 0.76)	-0.04 (-0.71 to 0.63)	2.19 (0.36 to 4.05)	2.38 (0.56 to 4.24)	-2.87 (-5.13 to -0.55)	-3.09 (-5.34 to -0.79)	<.001	<.001	<.001
Gestational diabetes	-0.43 (-1.00 to 0.14)	-0.57 (-1.13 to -0.02)	0.85 (-0.74 to 2.47)	0.91 (-0.64 to 2.49)	-0.58 (-2.72 to 1.61)	-0.87 (-2.94 to 1.25)	0.01	0.71	0.06
Diagnosed depression	0.81 (0.41 to 1.22)	0.78 (0.38 to 1.18)	0.68 (-0.36 to 1.74)	0.73 (-0.31 to 1.79)	-1.28 (-2.60 to 0.06)	-1.37 (-2.68 to -0.03)	0.91	<.001	<.001
Moderate to severe depression [f]	0.05 (-0.47 to 0.57)	0.05 (-0.47 to 0.57)	-1.33 (-2.76 to 0.13)	-1.30 (-2.74 to 0.15)	2.08 (0.02 to 4.19)	2.08 (0.02 to 4.18)	0.02	<.01	<.001
Venous thromboembolism	-1.21 (-4.14 to 1.84)	-1.26 (-4.20 to 1.76)	-0.28 (-8.63 to 8.88)	-0.25 (-8.61 to 8.88)	4.05 (-7.77 to 17.83)	3.88 (-8.06 to 17.37)	0.76	0.26	0.45
**Secondary newborn outcomes**									
Transient Tachypnea	-1.07 (-3.94 to 1.92)	-1.13 (-4.01 to 1.84)	-0.50 (-8.51 to 8.27)	-0.37 (-8.41 to 8.36)	9.14 (-2.21 to 22.26)	8.93 (-2.55 to 21.77)	0.81	0.02	0.08
5-minute APGAR score <7	0.38 (-1.43 to 2.25)	0.32 (-1.50 to 2.17)	-1.59 (-6.42 to 3.51)	-1.50 (-6.34 to 3.59)	2.54 (-4.24 to 9.91)	2.39 (-4.41 to 9.69)	0.34	0.43	0.21
Small for gestational age	0.59 (-0.06 to 1.26)	0.67 (0.01 to 1.33)	-1.29 (-3.04 to 0.50)	-1.36 (-3.11 to 0.42)	0.57 (-1.86 to 3.08)	0.70 (-1.74 to 3.20)	<.01	0.97	0.06
Large for gestational age	-0.25 (-0.75 to 0.25)	-0.34 (-0.84 to 0.16)	1.23 (-0.15 to 2.63)	1.26 (-0.11 to 2.65)	-1.66 (-3.47 to 0.18)	-1.75 (-3.53 to 0.08)	<.01	0.04	<.001

Individuals with delivery in T3 experienced the multimodal prenatal healthcare delivery for the entire duration of the pregnancy.
Interrupted time-series methodology for the trends in prenatal outcomes before and during the implementation of COVID-19 pandemic-related changes in prenatal healthcare used piecewise linear segmented regression, allowing a change in slope associated with time at two inflection points: 3/1/20 (initiation of changes in prenatal healthcare) and 12/6/20 (date which pregnancies were fully exposed to the prenatal healthcare changes). Log binomial regression was used to examine each binary outcome in relation to date of delivery (week), providing point and interval estimates of relative percent change in outcome probability associated with change in time, scaled to a 4-week change. Poisson regression was used for point and interval estimation of relative percent change in means for outcomes characterized as counts in relation to a 4-week change in delivery date.
[a] Adjusted for age, pre-pregnancy body mass index and neighborhood deprivation index.
[b] Two side Wald test adjusted for age, pre-pregnancy body mass index and neighborhood deprivation index. [c] Screening by 50-g, 1-h oral glucose challenge test; [d] Screening by Patient Health Questionnaire.
[e] Severe Maternal morbidity includes at least one of the following morbidities occurring at any time during pregnancy: acute myocardial infarction, aneurysm, acute renal failure, adult respiratory distress syndrome, amniotic fluid embolism, cardiac arrest/ventricular fibrillation, conversion of cardiac rhythm, disseminated intravascular coagulation, eclampsia, heart failure/arrest during surgery or procedure, puerperal cerebrovascular disorders, pulmonary edema/acute heart failure, severe anesthesia complications, sepsis, shock, sickle cell disease with crisis, air and thrombotic embolism, blood products transfusion, hysterectomy, temporary tracheostomy, or ventilation.[10-12]
[f] Patient Health Questionnaire screening scores (moderate to severe≥10).[13]

© 2023 Ferrera A et al. *JAMA Network Open.*

eTable 11. Interrupted Time Series Analysis for the Trends in Prenatal Visits, Care Process, and Health Outcomes in Association With Exposure to COVID-19 Pandemic–Related Implementation of a Multimodal Prenatal Health Care Delivery Among 52 129 **White** Individuals Who Who Delivered a Singleton Live Birth or Stillbirth, Kaiser Permanente Northern California, 2018-2021

	Percent Change (95% CI) per 4 weeks						P value [b]		
	**Unexposed** 7/1/18-2/29/20 T1		**Partially Exposed** 3/1/20-12/5/20 T2		**Fully Exposed** 12/6/20-10/31/21 T3				
	Unadjusted	Adjusted [a]	Unadjusted	Adjusted [a]	Unadjusted	Adjusted [a]	T2 vs. T1	T3 vs. T1	T3 vs. T2
**Prenatal care process**									
Total prenatal in-office and virtual clinic visits per individual	-0.26 (-0.32 to -0.21)	-0.29 (-0.34 to -0.23)	0.45 (0.29 to 0.60)	0.52 (0.36 to 0.67)	-0.37 (-0.59 to -0.15)	-0.47 (-0.69 to -0.25)	<.001	0.03	<.001
In office prenatal clinic visits per individual	-0.28 (-0.34 to -0.22)	-0.30 (-0.36 to -0.24)	-1.47 (-1.64 to -1.31)	-1.41 (-1.58 to -1.24)	2.81 (2.56 to 3.06)	2.71 (2.46 to 2.96)	<.001	<.001	<.001
Telemedicine prenatal clinic visits (video or telephone) per individual	0.26 (0.09 to 0.43)	0.20 (0.03 to 0.36)	9.62 (9.17 to 10.08)	9.77 (9.32 to 10.23)	-13.5 (-14.0 to -13.1)	-13.7 (-14.2 to -13.3)	<.001	<.001	<.001
Video prenatal visits per individual	17.25 (15.39 to 19.22)	17.04 (15.19 to 19.01)	18.18 (15.50 to 20.88)	18.48 (15.79 to 21.18)	-33.0 (-33.9 to -32.2)	-33.2 (-34.0 to -32.3)	0.22	<.001	<.001
Telephone prenatal visits per individual	0.41 (0.24 to 0.58)	0.36 (0.19 to 0.53)	3.40 (2.94 to 3.87)	3.54 (3.08 to 4.01)	-8.15 (-8.71 to -7.59)	-8.39 (-8.95 to -7.83)	<.001	<.001	<.001
**Prenatal care visits**									
Blood pressure measurements per individual	-0.03 (-0.07 to 0.01)	-0.06 (-0.10 to -0.02)	-0.80 (-0.91 to -0.69)	-0.69 (-0.80 to -0.58)	1.40 (1.24 to 1.56)	1.21 (1.05 to 1.37)	<.001	<.001	<.001
Gestational diabetes screening[c]	0.01 (-0.04 to 0.05)	0.01 (-0.04 to 0.06)	-0.06 (-0.19 to 0.08)	-0.07 (-0.20 to 0.06)	0.14 (-0.04 to 0.33)	0.13 (-0.05 to 0.31)	0.12	0.08	0.01
Depression screening [d]	-0.02 (-0.08 to 0.04)	-0.01 (-0.07 to 0.05)	-0.38 (-0.56 to -0.20)	-0.39 (-0.57 to -0.21)	0.95 (0.70 to 1.21)	0.97 (0.71 to 1.22)	<.001	<.001	<.001
**Primary maternal outcomes**									
Pre-eclampsia/Eclampsia	1.09 (0.42 to 1.76)	1.02 (0.35 to 1.68)	-0.54 (-2.29 to 1.24)	-0.44 (-2.19 to 1.34)	-1.58 (-3.93 to 0.84)	-1.68 (-4.04 to 0.73)	0.03	<.01	0.24
Severe maternal morbidity [e]	-0.37 (-1.29 to 0.56)	-0.42 (-1.34 to 0.51)	1.20 (-1.39 to 3.88)	1.34 (-1.27 to 4.01)	-0.40 (-3.88 to 3.23)	-0.60 (-4.08 to 3.01)	0.08	0.89	0.23
Cesarean section	0.21 (-0.08 to 0.51)	0.06 (-0.23 to 0.35)	-0.43 (-1.24 to 0.39)	-0.28 (-1.07 to 0.53)	0.79 (-0.35 to 1.94)	0.60 (-0.51 to 1.72)	0.28	0.19	0.08
Preterm birth	0.42 (-0.20 to 1.04)	0.36 (-0.26 to 0.98)	-1.12 (-2.82 to 0.61)	-1.01 (-2.71 to 0.72)	0.24 (-2.19 to 2.73)	0.08 (-2.34 to 2.56)	0.04	0.76	0.31
Spontaneous preterm birth	-0.12 (-0.97 to 0.73)	-0.13 (-0.98 to 0.72)	-1.23 (-3.62 to 1.21)	-1.17 (-3.55 to 1.28)	1.22 (-2.27 to 4.87)	1.11 (-2.39 to 4.73)	0.27	0.34	0.14
Medically indicated preterm birth	1.08 (0.14 to 2.04)	0.94 (0.00 to 1.90)	-1.13 (-3.62 to 1.43)	-0.95 (-3.44 to 1.61)	-0.72 (-4.16 to 2.86)	-0.95 (-4.38 to 2.60)	0.05	0.15	>.99
**Primary newborn outcomes**									
Neonatal intensive care unit admission	0.17 (-0.37 to 0.70)	0.10 (-0.43 to 0.63)	-1.77 (-3.25 to -0.26)	-1.69 (-3.17 to -0.18)	1.63 (-0.57 to 3.88)	1.53 (-0.66 to 3.76)	<.01	0.08	<.001

© 2023 Ferrera A et al. *JAMA Network Open.*

**Secondary maternal outcomes**									
Gestational hypertension	0.80 (0.28 to 1.32)	0.67 (0.16 to 1.19)	0.31 (-1.07 to 1.70)	0.47 (-0.90 to 1.86)	-0.71 (-2.52 to 1.13)	-0.91 (-2.70 to 0.92)	0.7	0.02	0.09
Gestational diabetes	-0.21 (-0.85 to 0.45)	-0.50 (-1.14 to 0.14)	-0.32 (-2.14 to 1.55)	0.09 (-1.71 to 1.93)	1.32 (-1.27 to 3.98)	0.61 (-1.92 to 3.19)	0.39	0.24	0.65
Diagnosed depression	0.80 (0.50 to 1.10)	0.75 (0.45 to 1.05)	0.60 (-0.19 to 1.40)	0.69 (-0.10 to 1.48)	-0.98 (-1.99 to 0.04)	-1.08 (-2.09 to -0.07)	0.84	<.001	<.001
Moderate to severe depression [f]	0.48 (-0.10 to 1.05)	0.42 (-0.15 to 0.99)	-2.18 (-3.75 to -0.58)	-1.95 (-3.52 to -0.35)	2.84 (0.51 to 5.25)	2.55 (0.24 to 4.92)	<.001	0.01	<.001
Venous thromboembolism	0.22 (-2.30 to 2.83)	0.05 (-2.48 to 2.64)	-4.74 (-11.7 to 2.68)	-4.51 (-11.4 to 2.94)	9.73 (-1.38 to 22.50)	9.37 (-1.86 to 21.87)	0.1	0.03	<.01
**Secondary newborn outcomes**									
Transient Tachypnea	-1.62 (-4.40 to 1.26)	-1.65 (-4.44 to 1.22)	6.28 (-1.66 to 15.02)	6.24 (-1.75 to 14.89)	0.90 (-8.29 to 11.23)	0.91 (-8.36 to 11.12)	0.01	0.48	0.25
5-minute APGAR score <7	-1.31 (-2.88 to 0.29)	-1.41 (-2.98 to 0.19)	5.23 (0.67 to 10.04)	5.31 (0.73 to 10.10)	-3.76 (-9.21 to 2.09)	-3.77 (-9.25 to 2.04)	<.001	0.27	<.001
Small for gestational age	0.28 (-0.33 to 0.91)	0.33 (-0.28 to 0.96)	-1.26 (-2.97 to 0.48)	-1.30 (-3.01 to 0.43)	0.69 (-1.77 to 3.22)	0.77 (-1.70 to 3.29)	0.01	0.64	0.06
Large for gestational age	-0.11 (-0.52 to 0.31)	-0.24 (-0.65 to 0.18)	0.32 (-0.84 to 1.49)	0.49 (-0.67 to 1.65)	0.09 (-1.50 to 1.72)	-0.11 (-1.69 to 1.50)	0.1	0.83	0.4

Individuals with delivery in T3 experienced the multimodal prenatal healthcare delivery for the entire duration of the pregnancy.

Interrupted time-series methodology for the trends in prenatal outcomes before and during the implementation of COVID-19 pandemic-related changes in prenatal healthcare used piecewise linear segmented regression, allowing a change in slope associated with time at two inflection points: 3/1/20 (initiation of changes in prenatal healthcare) and 12/6/20 (date which pregnancies were fully exposed to the prenatal healthcare changes). Log binomial regression was used to examine each binary outcome in relation to date of delivery (week), providing point and interval estimates of relative percent change in outcome probability associated with change in time, scaled to a 4-week change. Poisson regression was used for point and interval estimation of relative percent change in means for outcomes characterized as counts in relation to a 4-week change in delivery date.

[a] Adjusted for age, pre-pregnancy body mass index and neighborhood deprivation index.

[b] Two side Wald test adjusted for age, pre-pregnancy body mass index and neighborhood deprivation index.

[c] Screening by 50-g, 1-h oral glucose challenge test; [d] Screening by Patient Health Questionnaire.

[e] Severe Maternal morbidity includes at least one of the following morbidities occurring at any time during pregnancy: acute myocardial infarction, aneurysm, acute renal failure, adult respiratory distress syndrome, amniotic fluid embolism, cardiac arrest/ventricular fibrillation, conversion of cardiac rhythm, disseminated intravascular coagulation, eclampsia, heart failure/arrest during surgery or procedure, puerperal cerebrovascular disorders, pulmonary edema/acute heart failure, severe anesthesia complications, sepsis, shock, sickle cell disease with crisis, air and thrombotic embolism, blood products transfusion, hysterectomy, temporary tracheostomy, or ventilation.[10-12]

[f] Patient Health Questionnaire screening scores (moderate to severe≥10).[13]

© 2023 Ferrera A et al. *JAMA Network Open.*

eTable 12. Interrupted Time Series Analysis for the Trends in Prenatal Visits, Care Process, and Health Outcomes in Association With Exposure to COVID-19 Pandemic–Related Implementation of a Multimodal Prenatal Health Care Delivery Among 112 861 Individuals Who Had a **Low-Risk Pregnancy** Who Delivered a Singleton Live Birth or Stillbirth, Kaiser Permanente Northern California, 2018-2021

	Percent Change (95% CI) per 4 weeks						P value [b]		
	**Unexposed T1** 7/1/18-2/29/20 deliveries		**Partially Exposed T2** 3/1/20-12/5/20 deliveries		**Fully Exposed T3** 12/6/20-10/31/21 deliveries				
	Unadjusted	Adjusted [a]	Unadjusted	Adjusted [a]	Unadjusted	Adjusted [a]	T2 vs. T1	T3 vs. T1	T3 vs. T2
**Prenatal care visits**									
Total prenatal in-office and telemedicine visits per individual	-0.25 (-0.29 to -0.21)	-0.25 (-0.29 to -0.22)	0.47 (0.36 to 0.58)	0.50 (0.39 to 0.61)	-0.46 (-0.61 to -0.31)	-0.51 (-0.66 to -0.37)	<.001	<.001	<.001
In-office prenatal visits per individual	-0.23 (-0.27 to -0.19)	-0.23 (-0.27 to -0.19)	-1.57 (-1.68 to -1.45)	-1.54 (-1.65 to -1.43)	2.72 (2.55 to 2.89)	2.67 (2.50 to 2.84)	<.001	<.001	<.001
Telemedicine prenatal visits per individual	0.01 (-0.10 to 0.13)	-0.01 (-0.13 to 0.10)	10.39 (10.08 to 10.71)	10.46 (10.14 to 10.77)	-13.6 (-13.9 to -13.3)	-13.7 (-14.0 to -13.4)	<.001	<.001	<.001
Video prenatal visits per individual	15.19 (13.98 to 16.45)	15.05 (13.85 to 16.31)	20.49 (18.64 to 22.34)	20.81 (18.96 to 22.66)	-32.4 (-32.9 to -31.8)	-32.5 (-33.1 to -31.9)	<.001	<.001	<.001
Telephone prenatal visits per individual	0.14 (0.02 to 0.26)	0.13 (0.01 to 0.25)	4.73 (4.41 to 5.05)	4.75 (4.43 to 5.08)	-9.18 (-9.55 to -8.81)	-9.27 (-9.64 to -8.90)	<.001	<.001	<.001
**Prenatal care process**									
Blood pressure measurements per individual	-0.07 (-0.10 to -0.04)	-0.07 (-0.10 to -0.04)	-0.68 (-0.76 to -0.60)	-0.64 (-0.72 to -0.56)	1.36 (1.25 to 1.47)	1.24 (1.13 to 1.35)	<.001	<.001	<.001
Gestational diabetes screening [c]	0.03 (-0.01 to 0.06)	0.03 (-0.01 to 0.06)	-0.02 (-0.12 to 0.07)	-0.04 (-0.13 to 0.05)	0.09 (-0.05 to 0.23)	0.06 (-0.07 to 0.19)	0.06	0.47	0.08
Depression screening [d]	-0.00 (-0.05 to 0.04)	-0.00 (-0.05 to 0.04)	-0.31 (-0.43 to -0.18)	-0.31 (-0.44 to -0.19)	0.81 (0.64 to 0.99)	0.82 (0.65 to 0.99)	<.001	<.001	<.001
**Primary prenatal maternal outcomes**									
Pre-eclampsia/Eclampsia	0.65 (0.24 to 1.07)	0.59 (0.18 to 1.01)	0.11 (-1.00 to 1.23)	0.16 (-0.95 to 1.28)	-0.77 (-2.25 to 0.73)	-0.83 (-2.30 to 0.67)	0.31	0.01	0.14
Severe maternal morbidity [e]	0.12 (-0.51 to 0.75)	0.10 (-0.52 to 0.74)	0.85 (-0.85 to 2.59)	0.89 (-0.82 to 2.63)	-0.15 (-2.41 to 2.17)	-0.20 (-2.46 to 2.12)	0.23	0.72	0.29
Cesarean section	0.20 (-0.07 to 0.47)	0.12 (-0.15 to 0.39)	0.34 (-0.39 to 1.08)	0.41 (-0.32 to 1.15)	-0.38 (-1.37 to 0.62)	-0.45 (-1.43 to 0.55)	0.3	0.13	0.05
Preterm birth	-0.06 (-0.48 to 0.36)	-0.10 (-0.52 to 0.32)	-0.26 (-1.42 to 0.91)	-0.22 (-1.38 to 0.95)	0.48 (-1.14 to 2.13)	0.42 (-1.20 to 2.06)	0.79	0.4	0.38
Spontaneous preterm birth	-0.41 (-0.95 to 0.13)	-0.43 (-0.97 to 0.11)	-0.36 (-1.88 to 1.19)	-0.31 (-1.83 to 1.23)	1.04 (-1.13 to 3.26)	0.99 (-1.18 to 3.20)	0.84	0.08	0.18
Medically indicated preterm birth	0.50 (-0.19 to 1.20)	0.44 (-0.25 to 1.14)	-0.23 (-2.08 to 1.67)	-0.18 (-2.04 to 1.71)	-0.27 (-2.80 to 2.33)	-0.35 (-2.87 to 2.24)	0.39	0.41	0.88
**Primary newborn outcome**									
Neonatal intensive care unit admission	-0.36 (-0.74 to 0.03)	-0.39 (-0.78 to -0.00)	-1.04 (-2.13 to 0.06)	-1.01 (-2.10 to 0.09)	2.36 (0.77 to 3.98)	2.30 (0.70 to 3.91)	0.14	<.001	<.001

© 2023 Ferrera A et al. *JAMA Network Open.*

**Secondary prenatal maternal outcomes**									
Gestational hypertension	0.70 (0.32 to 1.08)	0.59 (0.21 to 0.96)	0.97 (-0.03 to 1.97)	1.10 (0.11 to 2.10)	-1.69 (-2.96 to -0.40)	-1.85 (-3.11 to -0.57)	0.18	<.001	<.001
Gestational diabetes	0.19 (-0.32 to 0.70)	-0.00 (-0.49 to 0.48)	0.56 (-0.82 to 1.96)	0.73 (-0.62 to 2.10)	-1.61 (-3.46 to 0.29)	-1.83 (-3.68 to 0.05)	0.16	<.01	<.01
Diagnosed depression	0.68 (0.42 to 0.93)	0.69 (0.44 to 0.94)	0.81 (0.15 to 1.48)	0.78 (0.13 to 1.43)	-0.82 (-1.66 to 0.04)	-0.88 (-1.71 to -0.04)	0.74	<.001	<.001
Moderate to severe depression [f]	0.24 (-0.11 to 0.59)	0.21 (-0.14 to 0.56)	-1.62 (-2.58 to -0.65)	-1.55 (-2.50 to -0.59)	2.42 (1.02 to 3.85)	2.37 (0.98 to 3.78)	<.001	<.001	<.001
Venous thromboembolism	-0.23 (-2.18 to 1.77)	-0.33 (-2.28 to 1.66)	-4.39 (-9.78 to 1.30)	-4.33 (-9.71 to 1.38)	11.62 (2.89 to 21.34)	11.48 (2.66 to 21.07)	0.06	<.001	<.001
**Secondary newborn outcome**									
Newborn Transient Tachypnea	-1.27 (-3.18 to 0.69)	-1.29 (-3.20 to 0.66)	2.33 (-3.07 to 8.08)	2.29 (-3.13 to 8.01)	5.77 (-1.33 to 13.53)	5.78 (-1.38 to 13.46)	0.09	<.01	0.29
Newborn 5-minute APGAR score <7	-0.55 (-1.66 to 0.58)	-0.60 (-1.72 to 0.53)	1.05 (-2.06 to 4.27)	1.13 (-1.99 to 4.34)	1.76 (-2.42 to 6.16)	1.64 (-2.55 to 6.02)	0.15	0.16	0.79
Birthweight small for gestational age	0.02 (-0.33 to 0.37)	-0.00 (-0.35 to 0.35)	0.15 (-0.81 to 1.12)	0.33 (-0.62 to 1.29)	-0.79 (-2.10 to 0.55)	-0.89 (-2.19 to 0.43)	0.37	0.07	0.04
Birthweight large for gestational age	-0.15 (-0.49 to 0.20)	-0.21 (-0.55 to 0.13)	0.11 (-0.85 to 1.08)	0.11 (-0.84 to 1.07)	0.71 (-0.61 to 2.05)	0.62 (-0.69 to 1.95)	0.38	0.09	0.38

individuals with delivery in T3 experienced the multimodal prenatal healthcare delivery for the entire duration of the pregnancy.

Interrupted time-series methodology for the trends in prenatal outcomes before and during the implementation of COVID-19 pandemic-related changes in prenatal healthcare used piecewise linear segmented regression, allowing a change in slope associated with time at two inflection points: 3/1/20 (initiation of changes in prenatal healthcare) and 12/6/20 (date which pregnancies were fully exposed to the prenatal healthcare changes). Log binomial regression was used to examine each binary outcome in relation to date of delivery (week), providing point and interval estimates of relative percent change in outcome probability associated with change in time, scaled to a 4-week change. Poisson regression was used for point and interval estimation of relative percent change in means for outcomes characterized as counts in relation to a 4-week change in delivery date.

[a] Adjusted for age, pre-pregnancy body mass index, neighborhood deprivation index and race/ethnicity.

[b] Two side Wald test adjusted for age, pre-pregnancy body mass index, neighborhood deprivation index and race/ethnicity. [c] Screening by 50-g, 1-h oral glucose challenge test; [d] Screening by Patient Health Questionnaire.

[e] Severe Maternal morbidity includes at least one of the following morbidities occurring at any time during pregnancy: acute myocardial infarction, aneurysm, acute renal failure, adult respiratory distress syndrome, amniotic fluid embolism, cardiac arrest/ventricular fibrillation, conversion of cardiac rhythm, disseminated intravascular coagulation, eclampsia, heart failure/arrest during surgery or procedure, puerperal cerebrovascular disorders, pulmonary edema/acute heart failure, severe anesthesia complications, sepsis, shock, sickle cell disease with crisis, air and thrombotic embolism, blood products transfusion, hysterectomy, temporary tracheostomy, or ventilation.[10-12]

[f] Patient Health Questionnaire screening scores (moderate to severe≥10).[13]

© 2023 Ferrera A et al. *JAMA Network Open.*

eTable 13. Interrupted Time Series Analysis for the Trends in Prenatal Visits, Care Process, and Health Outcomes in Association With Exposure to COVID-19 Pandemic–Related Implementation of a Multimodal Prenatal Health Care Delivery Among 8603 Individuals Who **Did Not Have a Low-Risk Pregnancy** Who Delivered a Singleton Live Birth or Stillbirth, Kaiser Permanente Northern California, 2018-2021

	Percent Change (95% CI) per 4 weeks						P value [c]		
	**Unexposed T1** 7/1/18-2/29/20 deliveries		**Partially Exposed T2** 3/1/20-12/5/20 deliveries		**Fully Exposed T3** 12/6/20-10/31/21 deliveries				
	Unadjusted	Adjusted [a]	Unadjusted	Adjusted [a]	Unadjusted	Adjusted [a]	T2 vs. T1	T3 vs. T1	T3 vs. T2
**Prenatal care visits**									
Total prenatal in-office and telemedicine visits per individual	-0.29 (-0.35 to -0.23)	-0.30 (-0.36 to -0.24)	0.38 (0.21 to 0.55)	0.39 (0.22 to 0.56)	-0.57 (-0.81 to -0.34)	-0.59 (-0.83 to -0.36)	<.001	<.001	<.001
In-office prenatal visits per individual	-0.30 (-0.37 to -0.24)	-0.31 (-0.37 to -0.24)	-1.62 (-1.81 to -1.44)	-1.62 (-1.80 to -1.43)	2.75 (2.47 to 3.02)	2.74 (2.46 to 3.01)	<.001	<.001	<.001
Telemedicine prenatal visits per individual	-0.00 (-0.16 to 0.15)	-0.02 (-0.18 to 0.14)	8.17 (7.73 to 8.61)	8.18 (7.75 to 8.62)	-11.9 (-12.3 to -11.4)	-11.9 (-12.4 to -11.5)	<.001	<.001	<.001
Video prenatal visits per individual	19.82 (17.42 to 22.42)	19.80 (17.40 to 22.40)	14.26 (11.05 to 17.46)	14.34 (11.13 to 17.54)	-31.2 (-32.2 to -30.3)	-31.4 (-32.3 to -30.4)	<.001	<.001	<.001
Telephone prenatal visits per individual	0.09 (-0.07 to 0.25)	0.08 (-0.08 to 0.24)	3.82 (3.38 to 4.27)	3.81 (3.36 to 4.25)	-8.56 (-9.09 to -8.02)	-8.55 (-9.08 to -8.02)	<.001	<.001	<.001
**Prenatal care process**									
Blood pressure measurements per individual	-0.04 (-0.08 to 0.00)	-0.06 (-0.10 to -0.01)	-0.78 (-0.90 to -0.66)	-0.78 (-0.90 to -0.66)	1.23 (1.06 to 1.40)	1.21 (1.04 to 1.38)	<.001	<.001	<.001
Gestational diabetes screening [b]	-0.02 (-0.06 to 0.03)	-0.01 (-0.06 to 0.03)	0.01 (-0.13 to 0.14)	-0.01 (-0.14 to 0.12)	0.01 (-0.17 to 0.20)	0.05 (-0.13 to 0.23)	0.88	0.33	0.48
Depression screening [d]	-0.06 (-0.13 to 0.01)	-0.05 (-0.12 to 0.02)	-0.11 (-0.31 to 0.10)	-0.12 (-0.32 to 0.08)	0.63 (0.36 to 0.91)	0.64 (0.37 to 0.91)	0.34	<.001	<.001
**Primary prenatal maternal outcomes**									
Pre-eclampsia/Eclampsia	1.36 (0.52 to 2.21)	1.29 (0.44 to 2.14)	-1.69 (-3.88 to 0.55)	-1.53 (-3.73 to 0.71)	-0.17 (-3.17 to 2.95)	-0.31 (-3.32 to 2.78)	<.01	0.16	0.37
Severe maternal morbidity [e]	0.26 (-0.61 to 1.14)	0.24 (-0.64 to 1.12)	-0.70 (-3.08 to 1.74)	-0.69 (-3.07 to 1.74)	3.18 (-0.10 to 6.60)	3.21 (-0.08 to 6.61)	0.31	0.02	<.01
Cesarean section	0.20 (0.02 to 0.39)	0.19 (0.01 to 0.38)	-0.43 (-0.94 to 0.08)	-0.45 (-0.96 to 0.05)	-0.18 (-0.88 to 0.54)	-0.11 (-0.81 to 0.60)	<.001	0.26	0.27
Preterm birth	0.98 (0.42 to 1.55)	0.93 (0.37 to 1.49)	-0.79 (-2.28 to 0.72)	-0.71 (-2.19 to 0.79)	-1.24 (-3.25 to 0.82)	-1.32 (-3.31 to 0.72)	<.01	<.01	0.51
Spontaneous preterm birth	0.52 (-0.37 to 1.43)	0.50 (-0.39 to 1.40)	-0.46 (-2.87 to 2.02)	-0.42 (-2.84 to 2.05)	-1.08 (-4.36 to 2.33)	-1.11 (-4.39 to 2.29)	0.33	0.2	0.65
Medically indicated preterm birth	1.32 (0.56 to 2.08)	1.24 (0.48 to 2.00)	-1.05 (-3.02 to 0.97)	-0.95 (-2.92 to 1.05)	-1.34 (-3.99 to 1.40)	-1.43 (-4.07 to 1.29)	<.01	<.01	0.7
**Primary newborn outcome**									
Neonatal intensive care unit admission	0.24 (-0.28 to 0.75)	0.20 (-0.31 to 0.72)	-0.81 (-2.23 to 0.64)	-0.84 (-2.25 to 0.60)	0.34 (-1.64 to 2.38)	0.38 (-1.60 to 2.40)	0.06	0.82	0.17

© 2023 Ferrera A et al. *JAMA Network Open.*

**Secondary prenatal maternal outcomes**									
Gestational hypertension	1.35 (0.63 to 2.08)	1.25 (0.52 to 1.98)	-1.61 (-3.49 to 0.30)	-1.42 (-3.30 to 0.49)	1.32 (-1.24 to 3.96)	1.04 (-1.51 to 3.66)	<.001	0.83	0.03
Gestational diabetes	-0.84 (-1.18 to -0.50)	-0.89 (-1.21 to -0.57)	0.66 (-0.33 to 1.67)	0.73 (-0.22 to 1.69)	0.37 (-1.02 to 1.78)	0.16 (-1.17 to 1.50)	<.001	0.03	0.33
Diagnosed depression	0.81 (0.43 to 1.20)	0.77 (0.39 to 1.15)	0.52 (-0.50 to 1.55)	0.48 (-0.52 to 1.49)	-0.99 (-2.28 to 0.34)	-0.98 (-2.25 to 0.31)	0.45	<.001	0.01
Moderate to severe depression [f]	0.14 (-0.44 to 0.72)	0.13 (-0.44 to 0.70)	-2.02 (-3.63 to -0.37)	-2.11 (-3.71 to -0.48)	2.87 (0.49 to 5.32)	3.05 (0.68 to 5.48)	<.001	<.001	<.001
Venous thromboembolism	0.12 (-2.87 to 3.25)	-0.03 (-3.04 to 3.06)	3.32 (-4.81 to 12.26)	3.51 (-4.67 to 12.38)	-8.23 (-18.0 to 2.77)	-8.35 (-18.1 to 2.53)	0.27	0.04	0.02
**Secondary newborn outcome**									
Newborn Transient Tachypnea	-1.26 (-4.40 to 2.00)	-1.24 (-4.38 to 2.00)	3.35 (-5.43 to 13.09)	3.26 (-5.55 to 12.90)	5.07 (-5.90 to 17.72)	5.07 (-6.04 to 17.49)	0.19	0.14	0.74
Newborn 5-minute APGAR score <7	-0.37 (-1.98 to 1.27)	-0.43 (-2.04 to 1.21)	1.31 (-3.19 to 6.03)	1.30 (-3.19 to 6.01)	-0.50 (-6.38 to 5.84)	-0.44 (-6.35 to 5.85)	0.32	>.99	0.53
Birthweight small for gestational age	0.25 (-0.42 to 0.93)	0.26 (-0.41 to 0.94)	0.34 (-1.52 to 2.23)	0.45 (-1.39 to 2.33)	-1.63 (-4.11 to 0.93)	-1.74 (-4.20 to 0.79)	0.79	0.03	0.05
Birthweight large for gestational age	0.02 (-0.44 to 0.49)	-0.07 (-0.52 to 0.39)	0.80 (-0.48 to 2.10)	0.86 (-0.40 to 2.14)	-2.19 (-3.89 to -0.46)	-2.19 (-3.86 to -0.48)	0.06	<.001	<.001

individuals with delivery in T3 experienced the multimodal prenatal healthcare delivery for the entire duration of the pregnancy.

Interrupted time-series methodology for the trends in prenatal outcomes before and during the implementation of COVID-19 pandemic-related changes in prenatal healthcare used piecewise linear segmented regression, allowing a change in slope associated with time at two inflection points: 3/1/20 (initiation of changes in prenatal healthcare) and 12/6/20 (date which pregnancies were fully exposed to the prenatal healthcare changes). Log binomial regression was used to examine each binary outcome in relation to date of delivery (week), providing point and interval estimates of relative percent change in outcome probability associated with change in time, scaled to a 4-week change. Poisson regression was used for point and interval estimation of relative percent change in means for outcomes characterized as counts in relation to a 4-week change in delivery date.

[a] Adjusted for age, pre-pregnancy body mass index, neighborhood deprivation index and race/ethnicity.

[b] Two side Wald test adjusted for age, pre-pregnancy body mass index, neighborhood deprivation index and race/ethnicity. [c] Screening by 50-g, 1-h oral glucose challenge test; [d] Screening by Patient Health Questionnaire.

[e] Severe Maternal morbidity includes at least one of the following morbidities occurring at any time during pregnancy: acute myocardial infarction, aneurysm, acute renal failure, adult respiratory distress syndrome, amniotic fluid embolism, cardiac arrest/ventricular fibrillation, conversion of cardiac rhythm, disseminated intravascular coagulation, eclampsia, heart failure/arrest during surgery or procedure, puerperal cerebrovascular disorders, pulmonary edema/acute heart failure, severe anesthesia complications, sepsis, shock, sickle cell disease with crisis, air and thrombotic embolism, blood products transfusion, hysterectomy, temporary tracheostomy, or ventilation.[10-12]

[f] Patient Health Questionnaire screening scores (moderate to severe≥10).[13]

© 2023 Ferrera A et al. *JAMA Network Open.*

eReferences

1. Urban and Rural. United States Census Bureau. https://www.census.gov/programs-surveys/geography/guidance/geo-areas/urban-rural.html
2. Kontopantelis E, Doran T, Springate DA, Buchan I, Reeves D. Regression based quasi-experimental approach when randomisation is not an option: interrupted time series analysis. *BMJ : British Medical Journal*. 2015;350:h2750. doi:10.1136/bmj.h2750
3. Penfold RB, Zhang F. Use of interrupted time series analysis in evaluating health care quality improvements. *Acad Pediatr*. Nov-Dec 2013;13(6 Suppl):S38-44. doi:10.1016/j.acap.2013.08.002
4. Mascha EJ, Sessler DI. Segmented Regression and Difference-in-Difference Methods: Assessing the Impact of Systemic Changes in Health Care. *Anesth Analg*. Aug 2019;129(2):618-633. doi:10.1213/ane.0000000000004153
5. McCullagh P. *Generalized linear models*. Routledge; 2019.
6. Van Buuren S, Oudshoorn K. *Flexible multivariate imputation by MICE*. 1999.
7. Rubin DB. Inference and Missing Data. *Biometrika*. 1976;(63):581-592. NOT IN FILE.
8. Sterne JAC, White IR, Carlin JB, et al. Multiple imputation for missing data in epidemiological and clinical research: potential and pitfalls. *BMJ*. 2009;338:b2393. doi:10.1136/bmj.b2393
9. Austin PC, White IR, Lee DS, van Buuren S. Missing Data in Clinical Research: A Tutorial on Multiple Imputation. *Can J Cardiol*. Sep 2021;37(9):1322-1331. doi:10.1016/j.cjca.2020.11.010
10. Kilpatrick SK, Ecker JL. Severe maternal morbidity: screening and review. *Am J Obstet Gynecol*. Sep 2016;215(3):B17-22. doi:10.1016/j.ajog.2016.07.050
11. Centers for Disease Control and Prevention. How Does CDC Identify Severe Maternal Morbidity? https://www.cdc.gov/reproductivehealth/maternalinfanthealth/smm/severe-morbidity-ICD.htm
12. Callaghan WM, Creanga AA, Kuklina EV. Severe maternal morbidity among delivery and postpartum hospitalizations in the United States. *Obstet Gynecol*. Nov 2012;120(5):1029-36. doi:10.1097/aog.0b013e31826d60c5
13. Kroenke K, Spitzer RL, Williams JB. The PHQ-9: validity of a brief depression severity measure. *JGenInternMed*. 2001;16(9):606-613. NOT IN FILE.

© 2023 Ferrera A et al. *JAMA Network Open.*
