# Supplementary material for: Prenatal Health Care Outcomes Before and During the COVID-19 Pandemic Among Pregnant Individuals and Their Newborns in an Integrated US Health System
Source: JAMA Netw Open. 2023 Jul 18;6(7):e2324011. doi: 10.1001/jamanetworkopen.2023.24011 (PMC10354684; doi:10.1001/jamanetworkopen.2023.24011)
Supplement: Supplement 2. — Data Sharing Statement [file jamanetwopen-e2324011-s002.pdf]

Data Sharing Statement

Ferrara. Prenatal Health Care Outcomes Before and During the COVID-19 Pandemic Among Pregnant Individuals and Their Newborns in an Integrated US Health System. *JAMA Netw Open*. Published July 18, 2023. doi:10.1001/jamanetworkopen.2023.24011

Data
Data available: Yes
Data types: Deidentified participant data
How to access data: request for data must be sent to assiamira.ferrara@kp.org
When available: With publication

Supporting Documents
Document types: None

Additional Information
Who can access the data: researchers whose proposed use of the data has been approved by their IRB and our IRB
Types of analyses: Only for replication of our findings
Mechanisms of data availability: after IRB approval of a proposal and with a signed data access agreement
